# Multifaceted Deactivation Dynamics of Fe(II) *N*-Heterocyclic Carbene Photosensitizers

**DOI:** 10.1021/acs.jpca.3c06983

**Published:** 2023-11-24

**Authors:** Linnea Lindh, Torbjörn Pascher, Samuel Persson, Yogesh Goriya, Kenneth Wärnmark, Jens Uhlig, Pavel Chábera, Petter Persson, Arkady Yartsev

**Affiliations:** † Division of Chemical Physics, Department of Chemistry, Lund University, Box 124, SE-22100 Lund, Sweden; ‡ Division of Computational Chemistry, Department of Chemistry, Lund University, Box 124, SE-22100 Lund, Sweden; § Center for Analysis and Synthesis (CAS), Department of Chemistry, Lund University, Box 124, SE-22100 Lund, Sweden

## Abstract

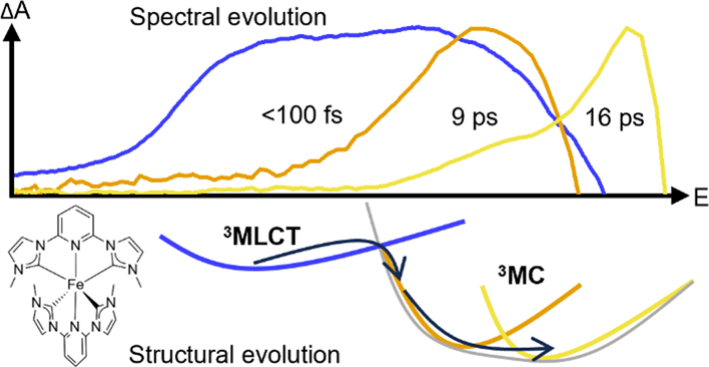

Excited state dynamics of three iron(II) carbene complexes that serve as prototype Earth-abundant photosensitizers were investigated by ultrafast optical spectroscopy. Significant differences in the dynamics between the investigated complexes down to femtosecond time scales are used to characterize fundamental differences in the depopulation of triplet metal-to-ligand charge-transfer (^3^MLCT) excited states in the presence of energetically accessible triplet metal-centered (^3^MC) states. Novel insights into the full deactivation cascades of the investigated complexes include evidence of the need to revise the deactivation model for a prominent iron carbene prototype complex, a refined understanding of complex ^3^MC dynamics, and a quantitative discrimination between activated and barrierless deactivation steps along the ^3^MLCT → ^3^MC → ^1^GS path. Overall, the study provides an improved understanding of photophysical limitations and opportunities for the use of iron(II)-based photosensitizers in photochemical applications.

## Introduction

The development of Earth-abundant light-harvesting complexes has garnered growing interest for solar energy conversion and photocatalysis applications in recent years.^1−5^ Iron-based complexes have received particular attention as an obvious candidate to replace the widely used Ru(II) congener complexes.^6−11^ Excitation into the metal-to-ligand charge-transfer (MLCT) states of traditional prototype Fe(II) complexes typically results in rapid deactivation either back to the ground state (GS), or resulting in the population of a long-lived quintet metal-centered (^5^MC) state.^12−18^ Complexes of the latter type may find use as light-induced excited state spin trapping (LIESST) compounds,^19^ but unfortunately are not very promising for photocatalytic and solar cell applications.^18^ This has spurred an intense interest in investigating the ultrafast excited state dynamics of photoexcited Fe(II) complexes as a key step to ultimately overcoming the photochemical limitations in these complexes, with particular focus on the role of triplet metal-centered (^3^MC) states. Such states serve as important but often short-lived intermediate states that can be accessible from the ^3^MLCT states in Fe(II) complexes. This is because the ^3^MC and ^3^MLCT states are unfavorably close in energy due to the intrinsically weak ligand field in first-row transition metal complexes.^20^

**Scheme 1:**
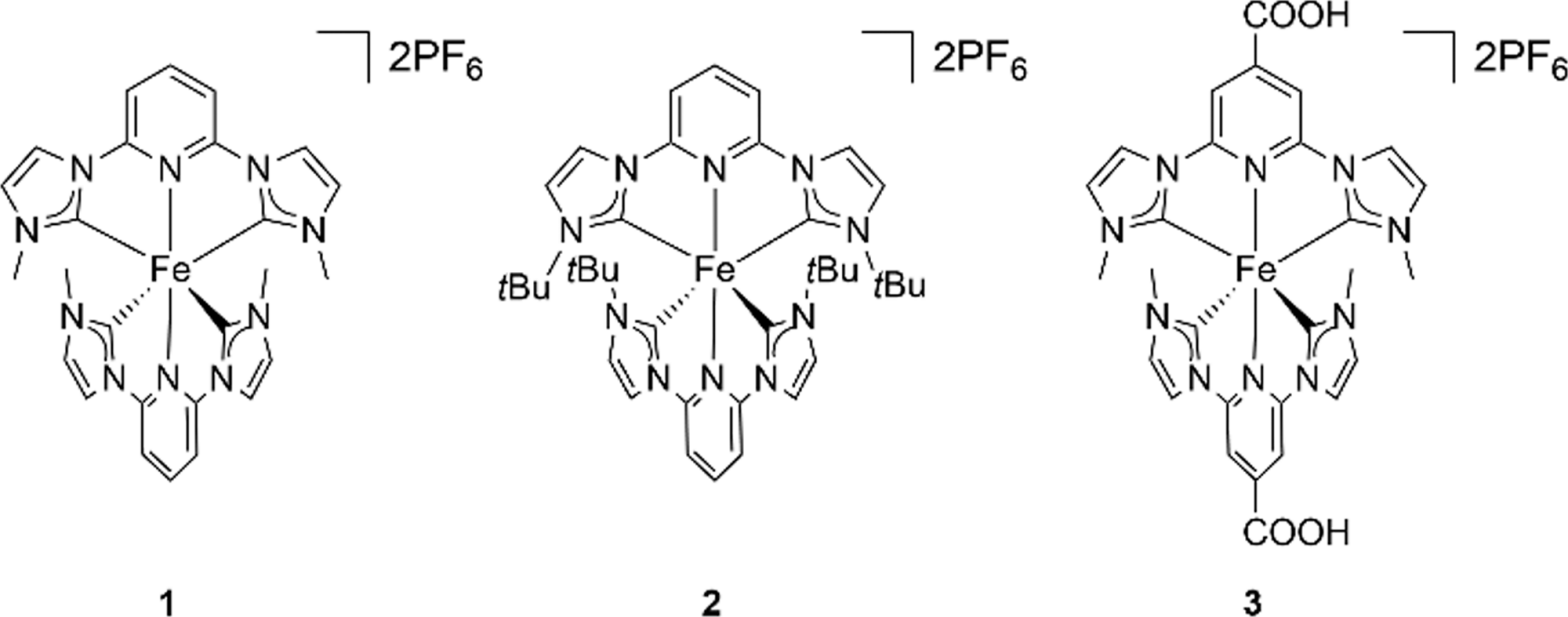
Iron Carbene Complexes **1**–**3** Discussed in This Communication

Strongly sigma-donating *N*-heterocyclic carbene (NHC) ligands were introduced as a new and promising strategy to prolong the MLCT lifetime of Fe(II) complexes,^6,7,21,22^ and early examples of this type of complexes included the three Fe(II) *N*-heterocyclic complexes: **1** [bis(1,1’-(pyridine-2,6-diyl)bis(3-methylimidazol-2-ylidene))iron(II)] bis(hexafluorophosphate),^22^**2** [bis(1,1’-(pyridine-2,6-diyl)bis(3-*tert*-butylimidazol-2-ylidene))iron(II)] bis(hexafluorophosphate),^22^ and **3** [bis(1,1’-(4-carboxypyridine-2,6-diyl)bis(3-methylimidazol-2-ylidene))iron(II)] bis(hexafluorophosphate)^23,24^ shown in Scheme 1. Complex **1** was the first Fe(II) carbene complex that showed promising photophysical performance with an at the time unprecedented excited state lifetime of 9 ps attributed to a ^3^MLCT state.^22^ Side groups attached to one of the nitrogens of the imidazole moieties had a strong influence on the photophysics, where the steric hindrance of the bulky *tert*-butyl side groups in complex **2** reverts the photophysics back to a long-lived (230 ps) ^5^MC state. In complex **2**, the ^3^MLCT state decays on subpicosecond time scale characterized by the loss of visible ESA and explained by the unfavorable excited state ordering similar to a LIESST compound.^22,25^ On the other hand, the electron-withdrawing carboxylic acid anchor groups in complex **3**, attached to the para-position of each of the pyridine moieties, stabilize the MLCT states which results in redshifted absorption and a doubling of the ^3^MLCT excited state lifetime compared to complex **1**.^23,24^ Since these pioneering studies, several iron carbene complexes have been developed with significantly further improved excited state lifetimes of, e.g., ∼0.5 ns,^26^ and elucidating the photophysical significance of factors such as the number of carbenes^9,27^ and stereochemistry.^28,29^ Recent efforts to increase the ligand field strength around the metal center have even enabled the development of photosensitizers with Fe(III)/d^5^ electronic structure rather than the more conventional Fe(II)/d^6^ motif.^30,31^ Recent successes to extend the excited ^3^MLCT state lifetimes of Fe(II) and related first-row d^6^ complexes also include extensions to use other ligand motifs—e.g., the Fe(II)-complex with diarylamido ligand by Braun et al.^32,33^—as well as alternative metal ions—e.g. Cr^0^.^34^

**Figure 1:**
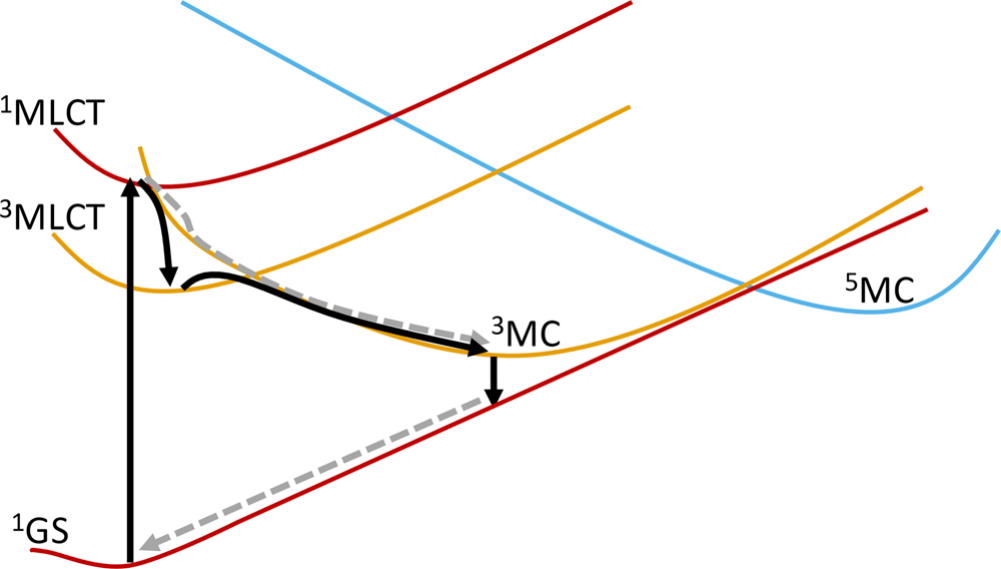
Schematic deactivation models for Fe carbene photosensitizers. The original model for Fe carbene photophysics involves initial relaxation into a long-lived ^3^MLCT state that decays back to the ^1^GS via a short-lived ^3^MC state (solid black arrows). A more elaborate three-state branching model recently proposed for complex 1 includes ultrafast branching of a significant part of the initially excited population to the ^3^MC state in addition to the original ^3^MLCT channel as well as slow GS cooling (solid black and dashed gray arrows).

The common understanding of the optical signatures of iron carbene complexes is still largely based on the early findings from the prototype Fe(II) d^6^ complexes **1**–**3**.^6,21−24^ The original model for complex **1** entailed an ultrafast intersystem crossing (ISC) from singlet to triplet MLCT, followed by a decay of the ^3^MLCT state via the ^3^MC state back to GS. Since the lifetime of the ^3^MLCT state was concluded to be much longer (at least 4 times) than the ^3^MC state, the observed GS recovery was dominated by 9 ps assigned to the ^3^MLCT lifetime. This model is depicted in Figure 1 by the solid black arrows. Later, a refined model was introduced in a recent ultrafast X-ray study by Kunnus et al. to include ultrafast branching from hot MLCT states with 60% of the population going to the relaxed ^3^MLCT state and 40% directly to the ^3^MC state.^35^ This followed a hot branching model introduced for a related Fe(II) carbene complex.^36^ In the work by Kunnus et al., the ^3^MLCT and ^3^MC excited state lifetimes of complex **1** were given as 9 and 1.5 ps, respectively.^35^ Furthermore, an additional slow component of 16 ps was found and attributed to GS cooling.^35^ This model, here referred to as the three-state branching model, is illustrated in Figure 1 by the combined decay channels indicated by the solid black and dashed gray arrows. Similarly, for complex **3** the original model was refined by Hainer et al. using vibrational coherence spectroscopy to include ultrafast branching of a minor part of the population going to the ^3^MC state instead of the relaxed ^3^MLCT state.^37^ These recent findings, developing the photophysical models for several prototype iron carbene complexes, highlight the need to revisit the ultrafast dynamics by transient absorption (TA) spectroscopy in order to enable a systematic comparison between closely related complexes with apparently very different photophysics.

In this work, we present results from ultrafast optical TA spectroscopy for the set of prototype complexes **1**–**3**. We particularly relied on optimized experimental conditions, including minimized excitation scatter via polarization filtering and minimized artifact via ultrathin cuvettes (detailed in the SI.1 and Methods section). This provides new and reliable information about the deactivation dynamics of these complexes in a broad spectral range, down to an instrument response function (IRF) limited resolution of 90 fs. First, we present results for complexes **2** and **3** as useful benchmark systems for more and less efficient deactivation of the initially populated charge-transfer states, respectively; before turning to the more complex dynamics of complex **1** where we also complemented the regular TA measurements of complex **1** with anisotropy, UV, and low-temperature TA measurements to further characterize the particularly intriguing behavior of this complex. Below, the results for the three investigated complexes are first presented one by one, before turning to a comparative analysis of the TA behavior of these complexes compared to other recent measurements as well as a broader consideration of how our new results add to the general understanding of the ultrafast dynamics of photoactive Fe(II) complexes.

## Methods

Complexes **1**, **2**, and **3** were synthesized according to literature procedures.^22,23^

TA spectroscopy was performed using an in-house built setup. The basis of this setup is a Solstice (Spectra Physics) laser amplifier system that produces ∼60 fs pulses at a central wavelength of 796 nm at a 4 kHz repetition rate. The amplifier output is divided into two parts that each pump a collinear optical parametric amplifier (TOPAS-C, Light Conversion). One of the TOPAS generates the pump beam (wavelength set to roughly the absorption maximum of each sample), while the other one generates a 1350 nm or a 400 nm beam that is focused onto a 5 mm CaF_2_ crystal to generate a supercontinuum probe beam (380–1100 nm or 280–380 nm). The delay between the pump and probe beams is introduced by a computer-controlled delay stage (Aerotech) placed in the probe beam’s path. After supercontinuum generation, the probe pulses are split into two parts: the former being focused to ∼100 μm spot size and overlapping with the pump pulse in the sample volume and the latter serving as a reference. After passing the sample, the probe beam is collimated again and relayed onto the entrance aperture of a prism spectrograph. The reference beam is directly relayed on the spectrograph. Both beams are then dispersed onto a double photodiode array, each holding 512 elements (Pascher Instruments). The intensity of excitation pulses was set to 1 mW corresponding to ∼10^14^ photons per pulse per cm^2^ and the spot-size ratio of pump and probe pulse was ∼1:30 in area. Mutual polarization between pump and probe beams was set to either the magic angle (54.7°) or 90° by placing a Berek compensator in the pump beam. In the case of perpendicular polarization between the beams, a Glan–Thompson polarizer was left after the sample to filter out excitation light scatter. Time-resolution of the setup after dispersion correction is estimated to be 90 fs.

Sample solutions were filled in either 1 mm optical path length cuvettes (Hellma—Optical Special Glass) or thin cuvettes of 100 μm optical path length (and thin windows of 200 μm where the beam transverses, Hellma—Optical Special Glass). Measurements were performed either at room temperature or at a controlled temperature by utilizing a bath cryostat (Oxford Instruments Optistat DN). The solvents used were for complex **1** and **2** dry acetonitrile (Sigma-Aldrich) except for temperature-dependent measurements of **1** where butyronitrile (Sigma-Aldrich) was used. For complex **3**, a buffer solution consisting of 0.1 M *tert*-butyl ammonium methanesulfonate and 0.1 M methanesulfonic acid in dry acetonitrile (Sigma-Aldrich) was used, to keep the complex in the desired protonated state. To check for the stability of each sample, steady-state absorption spectra were measured before and after TA experiments.

Before analysis, the measured data were corrected for group velocity dispersion (GVD—“chirp”) using the software KiMoPack^38^ or the software from Pascher Instruments. Based on the data quality, data were cut for excitation scatter (wavelength scale) and artifacts (time scale). Data were fitted by global analysis, where the signals at all wavelengths are fitted with the same sum of exponential decay components without assuming any model. For that, we used the software KiMoPack^75^ or the software from Pascher Instruments. Decay-associated spectra (DAS) are obtained from the wavelength dependent pre-exponential factor for each decay component given by the global analysis. Furthermore, target analysis was applied to the TA data of **1** by fitting different photophysical models in the software KiMoPack.^38^ Species-associated spectra (SAS) are obtained from the wavelength dependent pre-exponential factor for each species fitted in the model.

Single kinetic fits and GS cooling modeling was performed on the chirp-corrected data in the software Origin 2016. The single kinetic fitting consisted of a model of a sum of exponentials with amplitude and lifetime, together with a constant. The GS cooling fitting consisted of a model where two gaussians of the same area are summed; one with a negative amplitude and one with a positive amplitude that has a broader width than the negative one.

Anisotropy spectra and kinetics were calculated in Origin 2016 based on the chirp- and background-corrected data sets of complex **1**. The anisotropy signal was calculated by subtracting the perpendicular data set from the magic angle data set and then dividing the difference with the magic angle data set. The validity of the anisotropy signal was evaluated based on relative timing of the signals and the noise level (see SI.7).

## Results

### TA of Complex **3**

**Figure 2:**
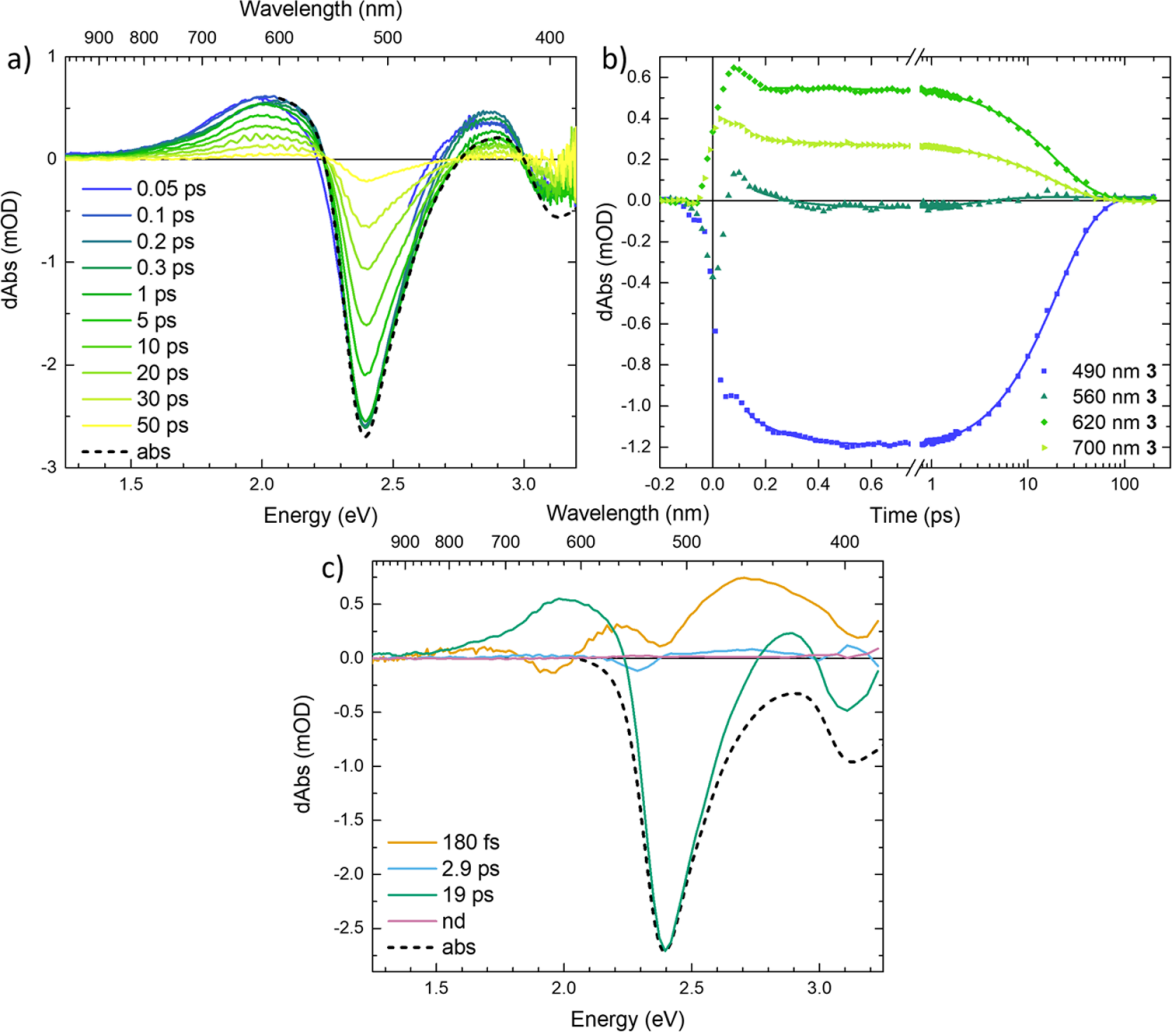
Transient absorption data of complex **3**, excitation wavelength 515 nm (2.4 eV). (a) Differential spectra at selected delay times, corrected for background and chirp. A region around 515 nm (2.4 eV) has been cut from the earliest two spectra to remove contributions from the Kerr effect (see SI.1 for details). (b) Transient absorption kinetics at selected wavelengths, corrected for background and chirp. Fits are plotted as solid lines. (c) Decay-associated spectra resulting from a global fit of the data. In this fit, a nondecaying (nd) component had to be included, probably to compensate for some setup-related artifact as it does not show any ground state bleach feature. In (a) and (c), the inverted linear absorption spectra (abs) are included for comparison.

The TA data measured for complex **3** are shown in Figure 2. We observe a pulse-limited buildup of the TA spectra consisting of an excited state absorption (ESA) feature partially overlapping with the ground state bleach (GSB) that later decays synchronously in the entire studied spectral region (Figure 2a). The positive part of the TA spectra displays a maximum centered at ∼620 nm (2.0 eV) that does not shift with time. There are, however, indications of spectral narrowing during the first 100s of femtoseconds which can be seen in a partial fast decay of the ESA >750 nm (<1.7 eV) and <600 nm (>2.1 eV). Similar dynamics were observed as a rise in the GSB kinetic with a negative sign at 490 nm (2.5 eV) due to a decay of the competing ESA in this spectral region (Figure 2b). The kinetic at 560 nm (2.2 eV), where at early times the dominant contribution changes from being GSB to ESA, displays no substantial dynamics after 300 fs. This point is thus close to an isosbestic point and indicates that we observe one species returning to GS. Thus, we conclude that apart from the very early TA dynamics, which is most probably related to vibrational relaxation, complex **3** undergoes conversion from the first observed excited state to the GS.

The TA data of complex **3** requires two main components of 180 fs and 19 ps to be fitted well by global analysis (see Figure 2c), although adding a minor (∼3 ps) component improved the quality of the fit somewhat. The 19 ps feature represents the strongest DAS component (Figure 2c) and resembles the characteristic TA spectra of complex **3**. Since complex **3** has the most stabilized ^3^MLCT state,^39^ we assign the 19 ps component ESA to this state. The DAS of the 180 fs component is also prominent and displays two dips: ∼650 nm (1.9 eV) on the red side of the ESA band and ∼520 nm (2.4 eV) on the blue side of the ESA band. This DAS indicates a spectral narrowing of the ESA band as the peak of the ESA grows and the sides decay. A very similar spectral dynamics were assigned previously to the narrowing of the ESA feature ∼620 nm (2.0 eV) as well.^37^ Single kinetics from the GSB region are well fitted by one decay component (see Figure SI.10 and Table SI.2). The GS recovery time from the ^3^MC state was estimated using a single-exponential fit to be less than 20 ps, which is only marginally longer than the estimated ^3^MLCT lifetime of around 18 ps. This suggests that the lifetime of the ^3^MC state has to be short compared to the rate-determining ^3^MLCT lifetime, in agreement with previous results from vibrational coherence spectroscopy.^37^ The component fitted with a lifetime of 2.9 ps could correspond to the decay of a ^3^MC state marginally populated by ultrafast branching discussed by Hainer et al.^37^ but is too weak to be safely assigned to any process. Overall, our results for complex **3** reaffirm the previously assigned model stating that a clear majority population resides in the ^3^MLCT state as the main long-lived excited state component (see Table 1).^23,24,37^

**Table 1:** Decay Processes for Complexes **1**–**3** According to Transient Absorption Results Fitted by Global Analysis

complex	^3^MLCT relaxation	^3^MLCT relaxation	^3^MC evolution	^3^MC decay	^5^MC decay
**1**		<100 fs	760 fs	9.2 ps, 16 ps	
**2**		160 fs		160 fs	320 ps
**3**	180 fs	19 ps		<2 ps*	

*From ref (37).

### TA of Complex **2**

**Figure 3:**
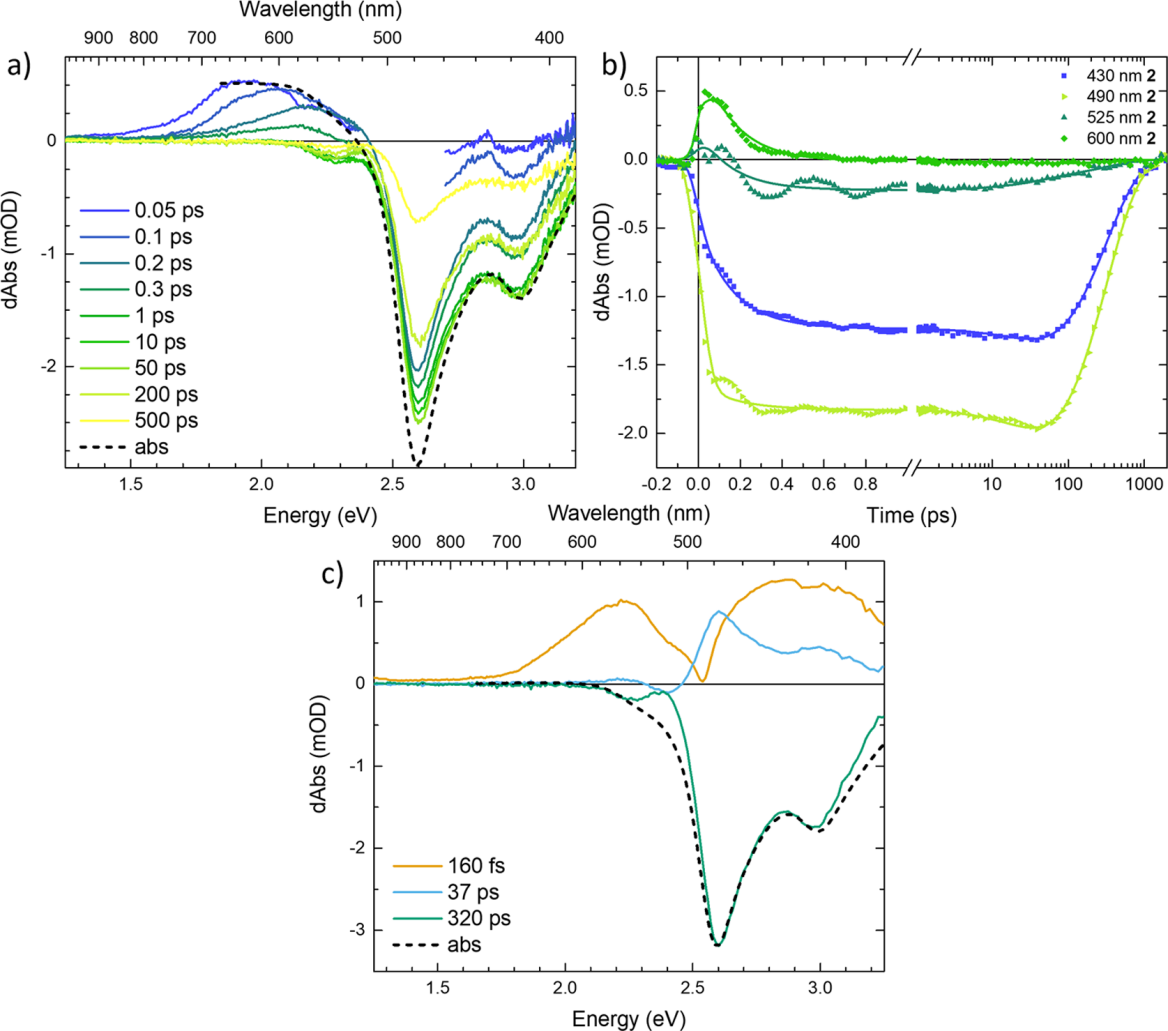
Transient absorption data of complex **2**, excitation wavelength 495 nm (2.5 eV). (a) Differential spectra at selected delay times, corrected for background and chirp. A region around 495 nm (2.5 eV) has been cut from the earliest two spectra to remove contributions from the Kerr effect (see SI.1 for details). (b) Transient absorption kinetics at selected wavelengths, corrected for background and chirp. Fits are plotted as solid lines. (c) Decay-associated spectra resulting from a global fit of the data. In (a) and (c), the inverted linear absorption spectra (abs) are included for comparison.

Next, we discuss complex **2** where the excited state dynamics were previously assigned to the decay cascade leaving the ^3^MLCT via ^3^MC states, finally ending up in a long-lived 200–300 ps ^5^MC state.^22,25^ Our new TA data of complex **2** are shown in Figure 3. At early (∼100 fs) delay times, we see a broad TA signal due to ESA covering the wavelength range of roughly 530–800 nm (1.6–2.3 eV) and turning negative due to GSB further toward blue. The ESA decays rapidly, also seen in the kinetic at 600 nm (2.1 eV) and as a rise in the negative kinetic at 430 nm (2.9 eV). It is worth noting that we see no indications of a decay of the GSB signal on a corresponding ultrafast time scale (the negative kinetics in Figure 3b). The GSB signal only recovers on the >100 ps time scale, while there is no visible ESA signal after 1 ps delay time. The only indication of an ESA feature is ∼520 nm (2.4 eV), where the TA spectrum is still negative on the picosecond time scale, but the spectral shape does not fit the measured steady-state absorption spectrum. In the UV range (280–380 nm (3.3–4.4 eV), see SI.2), the TA spectra show a strong ESA signal ∼320 nm (3.9 eV) that also survives to the >100 ps time scale (see Figure SI.5b).

The TA data of complex **2** require at least three components (160 fs, 37 and 320 ps) to be fitted well by global analysis.

The early ESA decay is captured by the 160 fs lifetime component, which was previously ascribed to the process of excited population leaving ^3^MLCT and ^3^MC states.^22^ The DAS corresponding to this process (Figure 3c) displays two broad ESA features separated by a dip. This dip is redshifted from the GSB peak, and we assign its appearance to a vibrational relaxation process that occurs on a similar time scale to the decay of the visible ESA (for details, see discussion on complex **1** below). The DAS of 320 ps mainly has the shape of GSB, however the same component in the UV TA data displays ESA (see Figure SI.7b). The lifetime is similar, though slightly longer, than the previously published ^5^MC lifetime ∼260 ps.^22,25^ The third fitted decay of 37 ps has a spectral shape that closely resembles GS absorption, so it is natural to associate this component with GSB rise. Although typically, after photoexcitation one would not expect any rise in the entire GSB region that is fully resolved in these experiments, for the specific setting of our experiment with the polarization of the probe perpendicular to that of the pump, the signal rises due to randomization of the transition dipole moments. This can be associated with reorientation dynamics^40,41^ and relaxation within the ^5^MC state. In the UV region, an 8 ps process is identified with DAS characteristic of narrowing of the ∼320 nm (3.9 eV) ESA spectrum (see Figure SI.7b) with negative amplitude on the sides of the ESA band and positive amplitude at the center of the band. We therefore assign these intermediate time processes to a delayed population and cooling of the ^5^MC state, similar to what was previously discussed.^22^ Overall, the observed dynamics of complex **2** agrees with previous findings (see Table 1).^22,25^

### TA of Complex **1**

**Figure 4:**
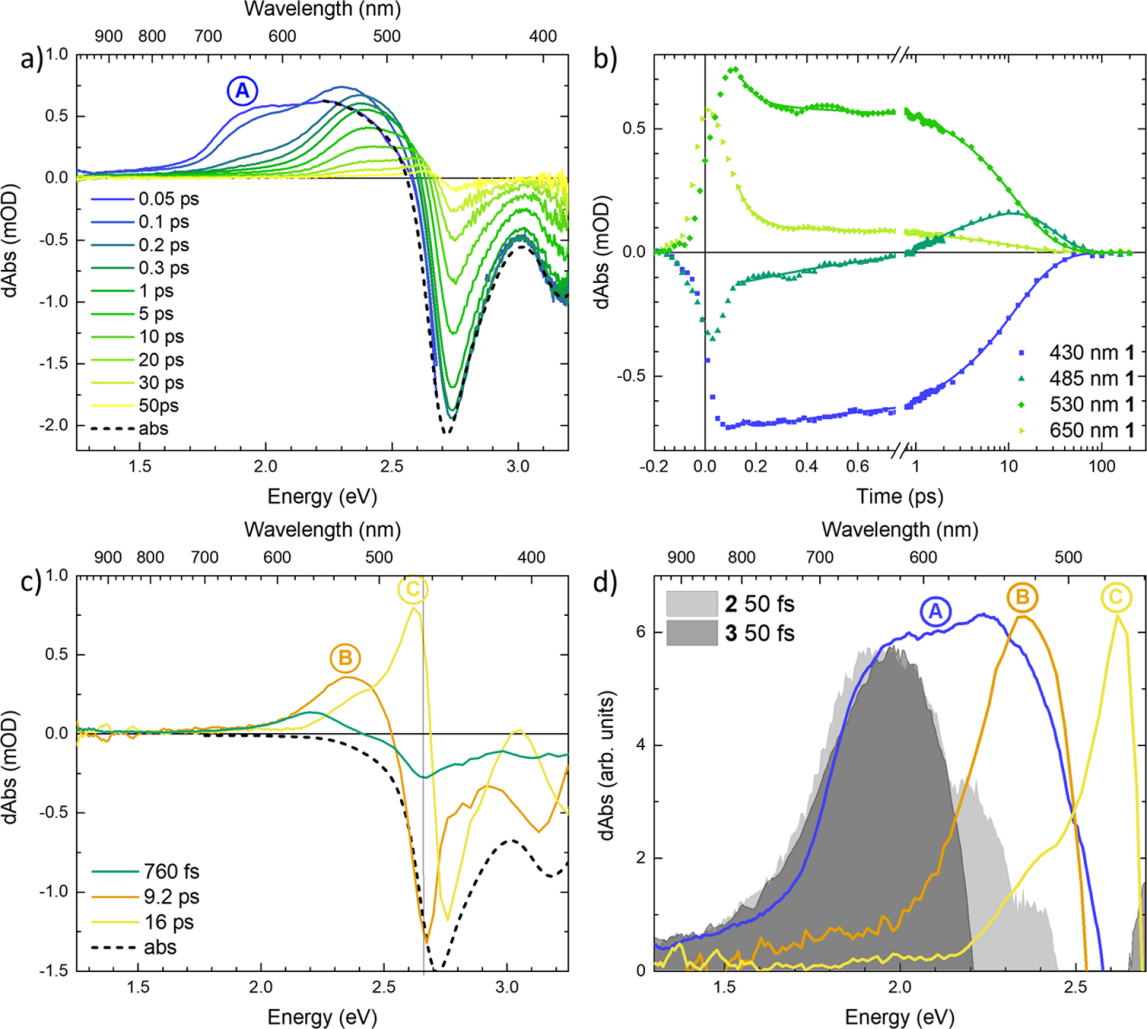
Transient absorption data of complex **1**, excitation wavelength 470 nm (2.6 eV). (a) Differential spectra at selected delay times, corrected for background and chirp. A region around 470 nm (2.6 eV) has been cut from the earliest two spectra to remove contributions from the Kerr effect (see SI.1 for details). (b) Transient absorption kinetics at selected wavelengths, corrected for background and chirp. Fits are plotted as solid lines. (c) Decay-associated spectra from global fitting. (d) Distinct spectral features A (from 50 fs differential spectrum, panel a), B and C (from 9.2 and 16 ps decay-associated spectra, panel c) in complex **1**. Feature A is also compared to the corresponding early excited state absorption spectra in complexes 2 and 3. The spectra are scaled to facilitate comparison. In (a) and (c), the inverted linear absorption spectra (abs) are included for comparison.

Finally, we turn our discussion to the TA of complex **1**. The TA signal in our new measurements is dominated by a positive ESA feature for wavelengths longer than ∼485 nm (2.6 eV) and a negative GSB region on the shorter wavelength side, as shown in Figure 4. The earliest (≤100 fs) TA spectra of complex **1** shown in Figure 4a show a broad ESA feature (labeled A) that extends toward ∼750 nm (1.7 eV). ESA feature A decays rapidly until the ESA region is instead dominated by another feature ∼530 nm (2.3 eV) (B, Figure 4c,d) already on a time scale close to the IRF. The ultrafast component of A dominates the kinetic at 650 nm (1.9 eV) and can also be seen to contribute to some extent in the kinetic at the 485 nm (2.6 eV) (i.e., in the ESA/GSB crossover region) shown in Figure 4b. However, an ultrafast decay process with a similar lifetime is not observed everywhere throughout the GSB spectral region as confirmed by the kinetic at 430 nm (2.9 eV) (see also Figure SI.9 and Table SI.1). The sub-100 fs dynamics can therefore be attributed to excited state evolution that involves the decay of the spectral feature A without corresponding GSB recovery. Furthermore, the ultrafast decay of the negative 485 nm (2.6 eV) signal has to be assigned to an increase of a new ESA that is not induced by the excitation but occurs simultaneously with the decay of the ESA feature A and corresponds to a buildup of the population in a new excited state. The reliability of this assignment is supported by the observation that the TA signals are much larger than the solvent response in the pump–probe pulse overlap region, implying that the solute response dominates the recorded dynamics (see SI.1 for more details).

Significant further TA evolution is seen on the >100 fs time scale. On the red side, the residual ESA >600 nm (<2.1 eV) continues to decay further on a few picosecond time scales as seen in the 650 nm (1.9 eV) kinetic in Figure 4b. The TA peak position associated with the ESA feature B blueshifts by 0.1 eV from 540 nm (2.3 eV) at 0.1 ps to 520 nm (2.4 eV) at 1 ps (see Figure SI.11) and decays on the picosecond time scale as seen in the 530 nm (2.3 eV) kinetic in Figure 4b. The blueshift of the ESA is most probably responsible for the change in the net TA signal at 485 nm (2.6 eV) in Figure 4b from negative at ∼0.1 ps to positive at ∼1 ps. This change of the TA signal sign suggests a buildup of strong ESA as GSB has a substantial contribution in this spectral region judging by the GS absorption spectrum. The blueshifted portion of the ESA close to 485 nm (2.6 eV) decays clearly slower than the main portion of ESA B centered at 530 nm (2.3 eV), which is obvious from the comparison of the last part of the respective kinetics in Figure 4b, suggesting that not all ESA decays with the same decay component. As a result of this delay, at ∼30 ps delay time one can observe a third characteristic ESA feature (C) peaking ∼485 nm (2.6 eV). The ESA C feature is, however, largely covered by GSB due to the substantial overlap in wavelength. It is important to note the TA signal at 485 nm (2.6 eV) keeps rising to 10 ps. The origin of this rise could be either partial recovery of the GS absorption or conversion of ESA B into ESA C. The picosecond dynamics of complex **1** were further corroborated by TA measurements in the UV range [280–380 nm (3.3–4.4 eV); see Figure SI.5a]. At 1 ps delay time, we observe a broad [310–360 nm (3.4–4.0 eV)] nearly triangular TA spectrum due to a sharp increase of the negative GSB contribution. At 20 ps delay time, the TA maximum position blueshifts and the spectral shape differs from early delay times. These changes are most probably associated with a faster decay of the red side of the ESA compared to its blue side (see Figure SI.6a).

The rich TA data of complex **1** requires at least four components to be fitted well by global analysis (see Table 1), including one decay component shorter than the IRF. No DAS for the <IRF component is shown, as the amplitude of any component on such short time scale could be substantially influenced by the IRF and may have no fundamental photophysical significance. The component must, however, inevitably include the decay of ESA A clearly seen in the red part of the early spectra and associated kinetics, and thus appear very similar to the earliest recorded TA spectra denoted ESA A; see Figure 4a,d. The two other characteristic ESA features B and C [with peaks at ∼530 nm (2.3 eV) and 485 nm (2.6 eV), respectively] identified above by inspection of the TA data were both found by the global analysis (see Figure 4c) and assigned lifetimes of 9.2 ps (B) and 16 ps (C), respectively. Two distinct ESA features with similar lifetimes were also found in the UV data, (see Figure SI.7a). Finally, a fourth component with a lifetime of 760 fs is needed for the fit. The DAS of this component agrees with a decay of the red and a rise of the blue sides of ESA B. Thus, we associate the 760 fs component with the blueshift and narrowing of the ESA due to vibrational relaxation. Single kinetic fitting of the GSB region largely agrees with the results found by global analysis, resulting in three similar >IRF components as in Figure 4c (see SI.3).

### Time-Resolved Anisotropy of Complex **1**

**Figure 5:**
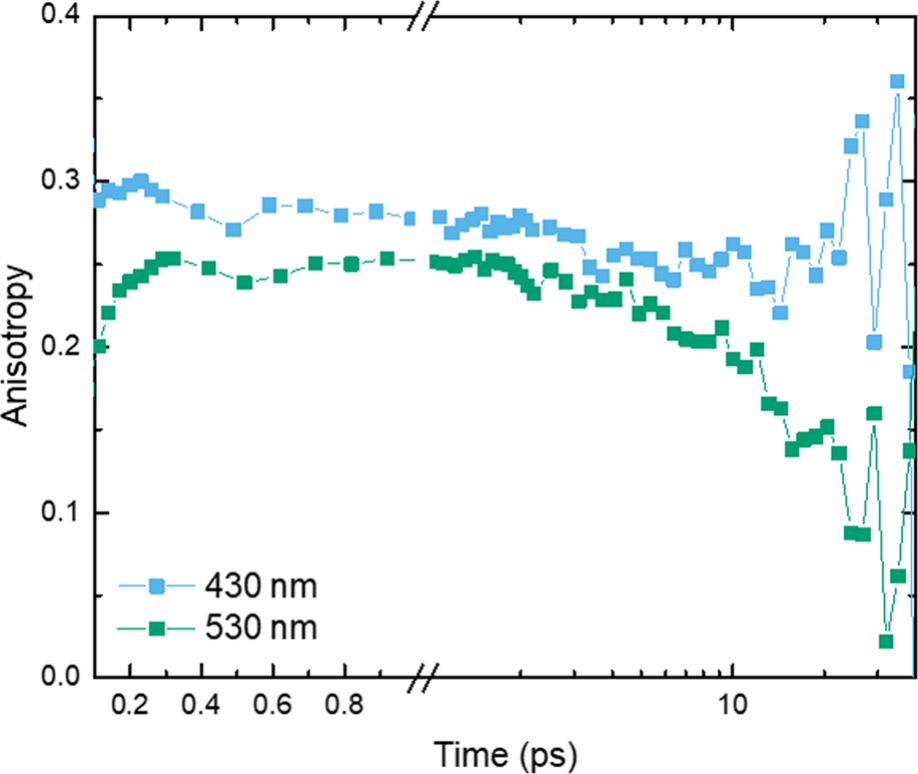
Anisotropy kinetics of complex **1** at selected wavelengths, corrected for the background and chirp. The 430 nm (2.9 eV) anisotropy is representative of the ground state bleach region, while the kinetic at 530 nm (2.3 eV) represents the excited state absorption anisotropy.

Next, the time-resolved anisotropy was calculated from TA data measured at magic angle and perpendicular polarization between pump and probe beams in the spectral range where pump scattering could be neglected (see SI.7 for details).^40−42^ Interestingly, the anisotropy value in the GSB region outside the pump scattering (see Figure SI.39) was calculated as ∼0.3, which is reasonably close to the expected GSB value of 0.4 for a linear oscillator.^41,42^ Such a high anisotropy value is most probably related to the axial symmetry of complex **1**. Furthermore, this high value of anisotropy decays very little until late delay times when the noise starts increasing (represented by the 430 nm kinetic in Figure 5). In the ESA wavelength range, an early anisotropy signal of ∼0.25 decays toward zero on a 10–30 ps time scale (represented by the 530 nm kinetic in Figure 5, for more details see SI.7). The clear difference in anisotropy dynamics in the GSB and ESA regions strongly suggests that the states contributing to the TA signal in these two spectral regions are of different nature.

### Temperature Dependent Dynamics of Complex **1**

**Figure 6:**
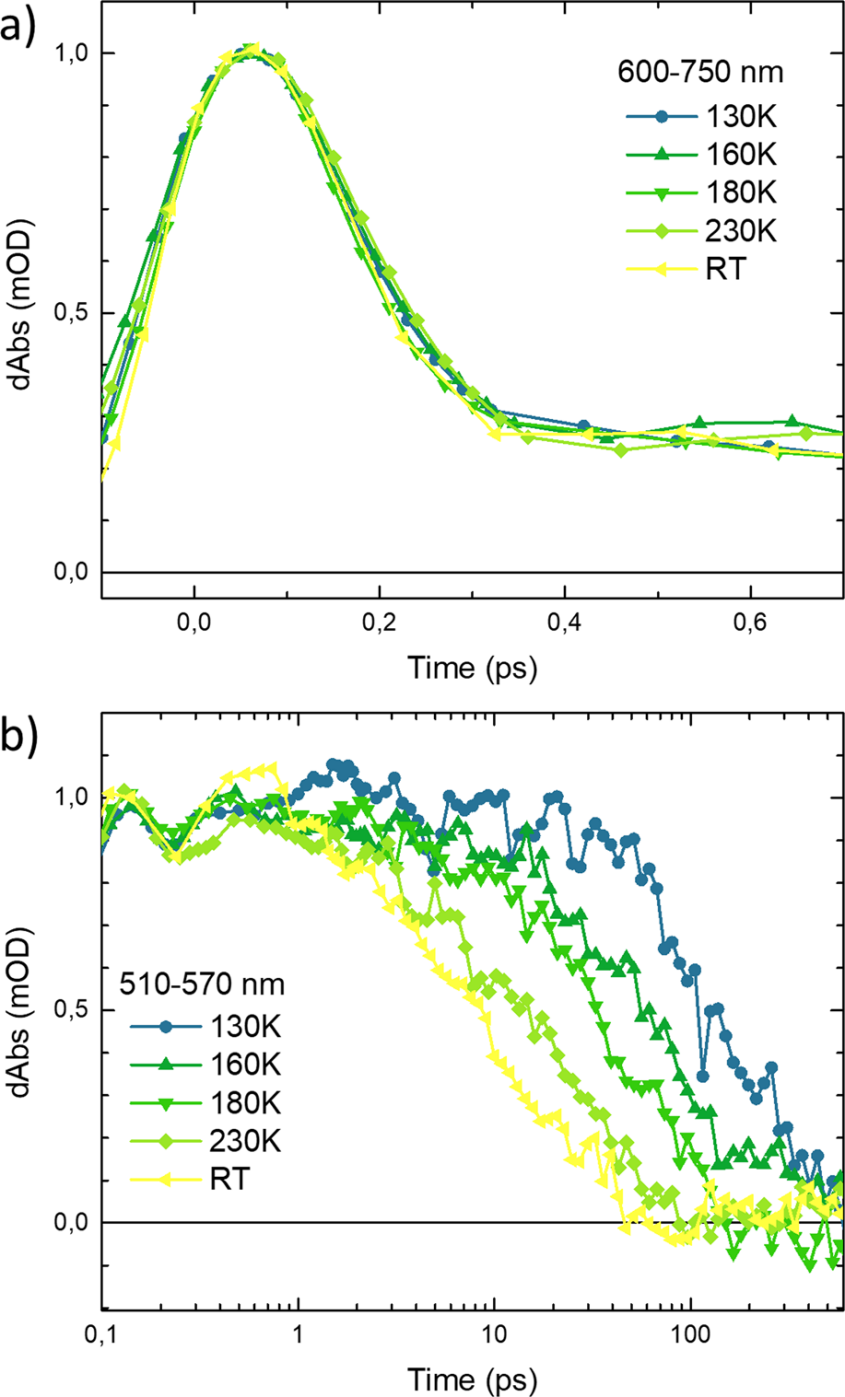
Temperature dependence kinetics of complex **1** to highlight (a) subpicosecond dynamics of the ESA A in the red wavelength range 600–750 nm (1.7–2.1 eV) and (b) later dynamics associated with the ESA B and C in the wavelength range 510–570 nm (2.2–2.4 eV). Kinetics have been corrected for background and chirp. RT: room temperature.

TA was measured for complex **1** in butyronitrile over a broad range of temperatures (130–300 K) to extract further information about the nature of the different deactivation steps. We start by concluding that the fast decay of ESA feature A in the 600–750 nm (1.7–2.1 eV) wavelength region is virtually unaffected by changing the temperature (never exceeding the IRF, Figure 6a). In the 510–570 nm (2.2–2.4 eV) spectral region dominated by the more long-lived ESA components B and C, the lifetime instead increases substantially as the temperature is lowered; see Figure 6b. Due to large scattering from the cryostat <510 nm (>2.4 eV), no attempt was made to further resolve any subtle differences between the ESA B and C components present in this wavelength region. The TA data in the >510 nm (<2.4 eV) wavelength range was fitted globally by a single-exponential decay to capture the main trend for the slow ESA decay (see SI.8). This yielded a ∼20-fold increase in ESA lifetime from ∼10 ps at room temperature to 190 ps at 130 K. The lifetime of this slow ESA decay above the glass transition temperature 139 K was fitted as a function of temperature by a simple form of the Arrhenius expression:
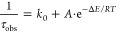


This equation contains one temperature-independent component (*k*_0_) and one activated term (*A*e^–Δ*E*/*RT*^). The result of this fit was a *k*_0_ of 8.8 × 10^9^ s^–1^, a pre-exponential factor, *A*, of 5.0 × 10^12^ s^–1^, and an activation energy, Δ*E*, of 790 cm^–1^ (98 eV); see SI.8 for more details.

## Discussion

### New Photophysics Model for Complex **1**

In the following, we assign the processes contributing to the TA dynamics in complex **1**. A comparison of the three studied complexes is crucial to rationalize the earliest (<100 fs) part of the observed dynamics. All complexes were excited at the red side of their respective ^1^MLCT absorption bands. ISC to the ^3^MLCT state is known to be very fast in Fe-complexes (≪100 fs) and is not resolved in this study.^43^ The initial broad characteristic ESA feature A (Figure 4a) in complex **1** resembles the early ESA observed in complexes **2** and **3** (Figure 4d) remarkably well. In complexes **2** and **3**, this ESA has been assigned to the ^3^MLCT state. For complex **3**, it was also proven that this spectrum indeed belongs to the ^3^MLCT state via triplet emission resolved by both fluorescence up-conversion spectroscopy^44^ and vibrational coherence spectroscopy.^37^ On this basis, we also assign the ESA feature A in complex **1** as a characteristic feature of the ^3^MLCT state.

Next, we note that the decay of the broad initial ESA feature A in <100 fs in complex **1** (Figure 4a) shows significant similarity to the decay of the corresponding early ESA decay in complex **2** (Figure 3a) both in terms of time scale and loss of ESA in the red part of the spectrum without simultaneous recovery in the GSB region. In complex **2**, this process was assigned to conversion of the MLCT to MC manifold.^22^ On the contrary, in complex **3** the initially generated spectral feature assigned to ^3^MLCT remains remarkably unchanged over the entire excited state lifetime. It is therefore natural to assign the early dynamics in complex **1** to efficient conversion of the ^3^MLCT state into the MC manifold on an ∼100 fs time scale. Furthermore, based on the absence of temperature dependence of the fast conversion, we conclude that this process is effectively barrierless.

Such fast population of MC states also fits with the oscillations seen in the kinetic at 525 nm (2.4 eV) (Figure SI.16). These oscillations have the period time 290 fs (see Table SI.3), which has previously been associated with populating MC states.^35^ Oscillations are observed in the early TA for all three complexes but are more prominent in complexes **1** and **2** where they clearly survive for several cycles (see Table SI.3). Furthermore, we assign the TA dynamics on subpicosecond time scale (760 fs DAS, Figure 4c) in complex **1** to the spectral shift of the ESA B feature, which we interpret as vibrational relaxation processes. This can be associated with the large changes in geometry that the complex undergoes when populating a MC state. Note that a similar shift is not observed for the ^3^MLCT state spectral feature in complex **3**.

Starting from ∼0.5 ps, we observe striking differences in the TA dynamics of complexes **1** and **2** where the rich TA dynamics in **1** is very different from a complete vanishing of the positive TA signal in complex **2**. Furthermore, in complex **2** the GS recovery occurs on ∼300 ps in contrast to ∼10 ps in complex **1**. Instead of comparing the dynamics of complex **1** to the ^5^MC state dynamics dominating complex **2**, we can compare it to dynamics of ^3^MC states observed in other iron complexes,^16,45−47^ potentially also including the expanded cage version of complex **1**,^48^ as well as some ruthenium complexes.^49,50^ These ^3^MC states have also displayed ESA features in the visible spectral range with lifetimes ranging from a few picosecond up to 100s of picoseconds. Taken together, the above considerations support the assignment of the ESA features B and C in complex **1** as ^3^MC but not as ^5^MC states. Multiple ^3^MC states of different symmetry and energy has been discussed from computational results for complex **2**(25) and other transition metal complexes.^51^

**Figure 7:**
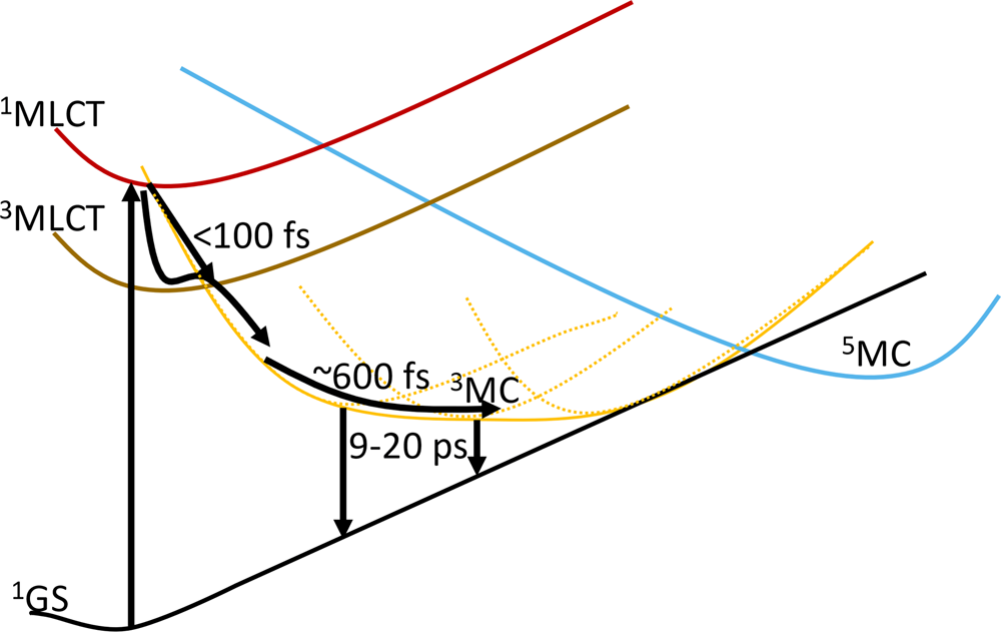
Photophysics model for complex **1** including complex deactivation dynamics via a multidimensional ^3^MC surface with several Jahn–Teller distorted local minima.

To rationalize the appearance of the ESA B and C components, we apply a target analysis in the global fitting of the >300 fs part of our data (where the contributions from ESA A are largely over). Two elementary models have been used to fit the data: (i) a consecutive model in which B converts to C, which further converts to GS, and (ii) a parallel model in which both B and C are populated on a sub-100 fs time scale and then decay independently to the GS, see SI.6 for further details. One additional process with a subpicosecond decay component is included in both models for satisfactory fits and assigned to vibrational relaxation in either B or C states. These models cannot be distinguished based on the goodness of fit, although they provide somewhat different rates and SAS. Obviously, a weighted linear combination of these models will also fit the data equally well. It is, however, important to note that the models are, in fact, fundamentally similar in many ways, including the ultrafast ^3^MLCT → ^3^MC conversion, ^3^MC relaxation, and relatively slow (∼10 ps) GS recovery. Moreover, both models are broadly consistent with an evolution of the excited population on a complex shallow and multidimensional ^3^MC energy landscape with several local Jahn–Teller distorted minima as typical in many different transition metal complexes.^52−58^ The population on this ^3^MC surface thus appears to be mixed between those minima and shows slightly different coupling to the GS. This interpretation of the deactivation pathways in complex **1** with fitted rates is summarized in Figure 7. See also Table 1 for a comparison between the assigned fitted rates in complexes **1**–**3**.

### Comparison to Previous Models

Having presented our new TA results for complexes **1**–**3**, we turn to a discussion about how these findings relate to our understanding of the photophysics of these complexes. In this, we first discuss how our results for the particularly intriguing dynamics of complex **1** relate to the recent discussion about ultrafast branching and deactivation via metal-centered states in complexes with intermediate ligand field strength. This is followed by a broader consideration of how the transition from CT to MC-dominated dynamics in the series of investigated complexes **1**–**3** enriches the general understanding of fast deactivation dynamics against emerging photofunctionality of Fe(II) and related first-row complexes more broadly.

First, we consider how our TA results for complex **1** relate to previously proposed models for the excited state dynamics.^22,35^ Contrary to Liu et al.,^22^ we assign the relatively strong ESA in **1** at later times to the ^3^MC state where we expect absorption of a CT-type, namely, of LMCT character to fill the hole in the highest occupied molecular orbital generated by MLCT excitation. To strengthen this argument, we note here that even for complex **2**, which at later times resides in the ^5^MC state, we observe a strong ESA of a CT character although shifted further in the blue. In the following, we consider the three-state branching model from Kunnus et al.^35^ as it was presented as a further development of the original ^3^MLCT excited state lifetime assignment by Liu et al.^22^ (Figure 1 with dashed lines). This perspective is important to arrive at a more comprehensive understanding of the multifaceted dynamics of this complex, although direct comparisons clearly must be done with significant caution, as there are considerable experimental differences. These include the different time-resolution (significantly improved in our new TA compared to previous TA), and use of different excitation wavelengths and fluencies (new TA excited closer to the absorption cutoff compared to the TA and time-resolved X-ray measurements in Kunnus et al.^35^).

We first note that our ultrafast TA results agree with the finding by Kunnus et al. that there is significant early dynamics that is naturally assigned to ∼100 fs decay of ^3^MLCT to ^3^MC, and that this ^3^MC population gives rise to characteristic oscillations in both X-ray and TA signals that are damped on a ∼ 0.5 ps time scale.^35^ A closer comparison of our TA results and proposed model (Figure 7) with the three-state branching model (Figure 1) nevertheless reveals several differences that need to be considered. While the three-state branching model included ultrafast ^3^MC population as a key element, it suggested that the majority population (60%) from the initial (∼110 fs) branching remains in the ^3^MLCT state, and that all ^3^MC population decays on a ∼1.5 ps time scale. Such substantial GS recovery from the 40% initially populated ^3^MC state should be easily identifiable as a significant fast GS recovery. In our data, we do not see any clear indication of such fast recovery prior to the main (∼10 ps) GS recovery dynamics.

Based on the ultrafast spectral evolution, we have assigned the lifetime of the initially populated ^3^MLCT state to <100 fs, and the subsequent slower TA dynamics to be dominated by the evolution in the ^3^MC manifold. One remaining question is whether our TA data could instead be interpreted more similarly to the MLCT-MC branching from the three-state branching model by assigning some of the ESA features (B or C) surviving on picosecond time scales to a significant remaining ^3^MLCT population. Here, we note that at least ESA feature C is seen experimentally to build up simultaneously with the decay of the ^3^MLCT feature A; i.e., ESA C is not immediately generated under excitation. The alternative assignment would fit the slower part (>0.3 ps) of our dynamics equally well from a pure mathematical modeling perspective (just replacing one or both of the long-lived ^3^MC excited states with a ^3^MLCT states in our consecutive/parallel models, see SI.6). As we still consider the early broad ESA feature A in our TA data to be the most unambiguous signature of ^3^MLCT character, this would constitute a fundamentally different interpretation of the photophysics by introducing two or three distinct ^3^MLCT states with substantially different spectra and lifetimes that we find to be less likely for the following reasons.

In order to discriminate between these two different photophysical models, we consider the characteristic properties of the triplet potential energy landscapes of the studied Fe(II) carbenes^39,59^ and related d^6^ complexes. The ^3^MLCT excited states typically form a set of closely overlapping/nested states. Any relaxation dynamics within the manifold of such ^3^MLCT states should therefore quickly end up in the lowest ^3^MLCT state in basic agreement with Kasha’s rule.^60^ We note that in complex **3** the shape of the ESA spectrum does not change much during thermalization within the manifold of the ^3^MLCT states. In the closely related complex **1**, we do not expect to observe a very different spectroscopic pattern of the ^3^MLCT relaxation dynamics while, in the experiment, we record a clearly different ESA evolution on both subpicosecond and ∼10 ps time scales. The intense development of ESA would agree with population dynamics on the ^3^MC energy landscapes, which contrary to the ^3^MLCT manifold is generally characterized by the presence of several structurally distinct local minima with elongated metal–ligand bonds that are associated with different Jahn–Teller distortions (cf. Figure 7).^52−58^ Overall, the rich ESA evolution observed in complex **1** is consistent with intricate population dynamics involving several distinct ^3^MC states rather than a set of ^3^MLCT states. The overall stabilization of the MLCT states of complex **3** compared to complex **1** (evident from the redshifted steady-state absorption)^23,24,39^ provides a natural explanation for why complex **3** remains in a ^3^MLCT state without undergoing similar ultrafast ^3^MLCT → ^3^MC deactivation as complex **1**.

Next, we consider to what extent the three-state branching model potentially still fits with the target analysis of our data following the initial, not fully resolved, ∼100 fs dynamics. First, we applied the three-state branching model with identical parameters as in Kunnus et al. (i.e., with locked ^3^MLCT lifetime of 9 ps, ^3^MC lifetime of 1.5 ps, and a branching ratio of 60:40)^35^ to our TA data. This model failed to fit our TA data (yielding significant nonrandom residuals, see Figure SI.20). We then considered several different modifications of the model to see what it would take to restore a good fit to our data. The best fit with this model involved a substantial variation of the branching between the ^3^MLCT and the ^3^MC states with >75% of the population going to ^3^MLCT, an additional lifetime component added in the model, together with locking the other lifetimes to the values from Kunnus et al.^35^ (see SI.6). Thus, the model developed in ref (35) cannot adequately reproduce the data in this study.

Finally, we consider the alternative interpretation for the slowest excited state decay process (>10 ps) in complex **1** as GS cooling of an initially hot GS.^35^ In our consecutive model, this would correspond to ESA feature B being the last surviving excited state that decays to a hot GS represented by ESA feature C. In this case, the differential spectral shape of feature C should consist of a combination of negative steady-state absorption and a broadened (i.e., hot), positive ground-state absorption (GSA), but we were unable to make such a model fit our data (see SI.4). Additionally, from analysis of the anisotropy dynamics in different spectral regions we concluded that TA on the red side [>520 nm (2.4 eV)] should be assigned to contribution of states different from the GS. We observe that at later times anisotropy decays to zero in this spectral region (see Figure 5) contrary to the anisotropy value observed in the GSB region. If feature C would correspond to the hot GSA at later times when both ESA A and B already largely decayed, the anisotropy of the red positive TA band should rise toward the same value as in the GSA region.

All in all, we have shown that our TA data provide a new and richer understanding into the specific dynamics for complex **1** on both fast and slow time scales that includes several new insights pertaining in particular to the complex role of the ^3^MC intermediate state for the excited state evolution. Furthermore, we have seen that the series of complexes **1**–**3** with the same metal-carbene core structure provides a remarkable series where subtle stabilization/destabilization effects imposed by different side groups completely change the fundamental photophysics.

The systematic comparison of the ultrafast TA dynamics between these complexes, however, also provides further insight beyond these overall observations. In particular, the subpicosecond TA reveals a striking similarity in the early ESA in all three complexes characterized by virtually identical ESA onsets ∼750 nm (1.7 eV) despite significantly different onsets of their steady-state absorption spectra. This provides a particularly good signature for the initially formed ^3^MLCT state, which is seen to remain remarkably robust in the ^3^MLCT-dominated complex **3**, undergoing substantial change to a more blueshifted ESA in the ^3^MC-dominated complex **1**, and essentially disappearing in the ^5^MC-dominated complex **2**. Although we currently have no quantitative support for a full assignment of all ESA features, we note that the observations for the ESA onset at ∼750 nm (1.7 eV) are qualitatively consistent with an orbital transition picture where the red-most ESA feature is associated with ligand-to-metal charge-transfer (LMCT) excitations from ligand π orbitals into the empty t_2g_ metal orbitals. As the complexes relax toward structurally Jahn–Teller distorted ^3^MC and ^5^MC states, this should change the energy of the empty t_2g_ metal orbitals toward higher energies as the occupied e_g_ metal orbitals decrease in energy, explaining the blueshift of the ESA. Furthermore, LMCT transitions can be both strong and show solvatochromism,^61^ which also resolves the initial argumentation in Liu et al. regarding the assignment of the ESA. Further investigations to explore a broader range of complexes will be interesting to see to what extent these ESA trends can be expanded to include other types of ligand modification motifs.

The set of complexes studied here, clarifying the very subtle transition between the ^3^MLCT–^3^MC–^5^MC balance, also contributes to the broader context of understanding trends for the ultrafast deactivation dynamics in Fe(II) and other Earth-abundant d^6^ complexes with small to moderate ligand field strengths. Ultrafast investigations of many Fe(II) complexes, including, e.g., traditional Fe(II) polypyridyl and cyanide complexes^12−14,16,17,43,45,62−68^ in addition to several recent studies of Fe(II) carbenes and other complexes with stronger ligand field ligand motifs^6,7,30−33,35,36,47,69−72^ have provided a wealth of information about ultrafast deactivation channels. This includes both evidence for ultrafast MLCT → MC cascade dynamics with transient population of the ^3^MC state,^14,15^ oscillations,^35,37,67,73^ and state-to-state crossings,^74^ as well as raising important questions about the ^3^MC–^5^MC crossing point in intermediate ligand field complexes,^16,75^ and GS recovery dynamics in low-spin/high-spin crossover complexes.^63^ Accessing the ultrafast TA dynamics over a range of temperatures provides spectroscopic evidence of the efficiency of the early MLCT → ^3^MC deactivation in complex **1** as a nonactivated process. From the slower dynamics, the subsequent GS recovery is instead seen to be governed by complex multiexponential dynamics with a small but significant activation barrier of 790 cm^–1^ (98 meV) that can be rationalized in terms of relaxation on a multidimensional ^3^MC surface. These new insights into the intricate ^3^MC dynamics are especially important, as such states provide a key to understanding the deactivation dynamics in many Earth-abundant complexes with low to intermediate ligand field strength.

## Conclusions

To conclude, we revisited the photophysics of the three d^6^ Fe carbene prototype complexes **1**–**3** by conducting TA measurements with significantly better temporal resolution and the signal-to-noise ratio of the measurements. For the two complexes **2** and **3**, we confirm the previously published description of the photoinduced processes. On the contrary, for complex **1** we provide a new description that includes the ^3^MLCT state converting to the ^3^MC manifold on a sub-100 fs time scale. Then, in the ^3^MC manifold, the excited state population evolves on a rather shallow MC surface with some local minima, yielding a distribution of decay times 10–20 ps. We associate the change in the ligand side groups with the relative arrangement of the excited states in energy that will decide the interconversion of the excited states. The set of complexes presented here nicely illustrate that with a small variation of the side group to the ligand, the relative position of MLCT and MC states can completely change from favoring the ^3^MLCT, the ^3^MC, or the ^5^MC states.

## Supporting Information Available

Additional transient absorption data and analysis of the data, together with evaluation of artifacts, oscillations, and sample stability (PDF)

## References

[ref1] Wenger O. S. Photoactive Complexes with Earth-Abundant Metals. J. Am. Chem. Soc. 2018, 140 (42), 13522–13533. 10.1021/jacs.8b08822. 30351136

[ref2] Förster C.; Heinze K. Photophysics and Photochemistry with Earth-Abundant Metals – Fundamentals and Concepts. Chem. Soc. Rev. 2020, 49 (4), 1057–1070. 10.1039/C9CS00573K. 32025671

[ref3] Hockin B. M.; Li C.; Robertson N.; Zysman-Colman E. Photoredox Catalysts Based on Earth-Abundant Metal Complexes. Catal. Sci. Technol. 2019, 9 (4), 889–915. 10.1039/C8CY02336K.

[ref4] Bozic-Weber B.; Constable E. C.; Housecroft C. E. Light Harvesting with Earth Abundant d-Block Metals: Development of Sensitizers in Dye-Sensitized Solar Cells (DSCs). Coord. Chem. Rev. 2013, 257 (21–22), 3089–3106. 10.1016/j.ccr.2013.05.019.

[ref5] Housecroft C. E.; Constable E. C. Solar Energy Conversion Using First Row d-Block Metal Coordination Compound Sensitizers and Redox Mediators. Chem. Sci. 2022, 13 (5), 1225–1262. 10.1039/D1SC06828H. 35222908 PMC8809415

[ref6] Lindh L.; Chábera P.; Rosemann N. W.; Uhlig J.; Wärnmark K.; Yartsev A.; Sundström V.; Persson P. Photophysics and Photochemistry of Iron Carbene Complexes for Solar Energy Conversion and Photocatalysis. Catalysts 2020, 10 (3), 315 10.3390/catal10030315.

[ref7] Kaufhold S.; Wärnmark K. Design and Synthesis of Photoactive Iron *N*-Heterocyclic Carbene Complexes. Catalysts 2020, 10 (1), 132 10.3390/catal10010132.

[ref8] Wenger O. S. Is Iron the New Ruthenium?. Chem.—Eur. J. 2019, 25 (24), 6043–6052. 10.1002/chem.201806148. 30615242

[ref9] Duchanois T.; Liu L.; Pastore M.; Monari A.; Cebrián C.; Trolez Y.; Darari M.; Magra K.; Francés-Monerris A.; Domenichini E.; Beley M.; Assfeld X.; Haacke S.; Gros P. NHC-Based Iron Sensitizers for DSSCs. Inorganics 2018, 6 (2), 63 10.3390/inorganics6020063.

[ref10] de Groot L. H. M.; Ilic A.; Schwarz J.; Wärnmark K. Iron Photoredox Catalysis – Past, Present, and Future. J. Am. Chem. Soc. 2023, 145 (17), 9369–9388. 10.1021/jacs.3c01000. 37079887 PMC10161236

[ref11] Abrahamsson M. Solar Energy Conversion Using Iron Polypyridyl Type Photosensitizers – a Viable Route for the Future? InPhotochemistry; Royal Society of Chemistry, 2017; Vol. 44, pp 285–295.

[ref12] Auböck G.; Chergui M. Sub-50-fs Photoinduced Spin Crossover in [Fe(bpy)_3_]^2+^. Nat. Chem. 2015, 7 (8), 629–633. 10.1038/nchem.2305. 26201738

[ref13] Cannizzo A.; Milne C. J.; Consani C.; Gawelda W.; Bressler C.; van Mourik F.; Chergui M. Light-Induced Spin Crossover in Fe(II)-Based Complexes: The Full Photocycle Unraveled by Ultrafast Optical and X-Ray Spectroscopies. Coord. Chem. Rev. 2010, 254 (21–22), 2677–2686. 10.1016/j.ccr.2009.12.007.

[ref14] Zhang W.; Alonso-Mori R.; Bergmann U.; Bressler C.; Chollet M.; Galler A.; Gawelda W.; Hadt R. G.; Hartsock R. W.; Kroll T.; et al. Tracking Excited-State Charge and Spin Dynamics in Iron Coordination Complexes. Nature 2014, 509 (7500), 345–348. 10.1038/nature13252. 24805234 PMC5668134

[ref15] Zhang W.; Gaffney K. J. Mechanistic Studies of Photoinduced Spin Crossover and Electron Transfer in Inorganic Complexes. Acc. Chem. Res. 2015, 48 (4), 1140–1148. 10.1021/ar500407p. 25789406

[ref16] Jamula L. L.; Brown A. M.; Guo D.; McCusker J. K. Synthesis and Characterization of a High-Symmetry Ferrous Polypyridyl Complex: Approaching the ^5^T_2_/^3^T_1_ Crossing Point for Fe^II^. Inorg. Chem. 2014, 53 (1), 15–17. 10.1021/ic402407k. 24341550

[ref17] Juban E. A.; Smeigh A. L.; Monat J. E.; McCusker J. K. Ultrafast Dynamics of Ligand-Field Excited States. Coord. Chem. Rev. 2006, 250 (13–14), 1783–1791. 10.1016/j.ccr.2006.02.010.

[ref18] Zigmantas D.; Polívka T.; Persson P.; Sundström V. Ultrafast Laser Spectroscopy Uncovers Mechanisms of Light Energy Conversion in Photosynthesis and Sustainable Energy Materials. Chemical Physics Reviews 2022, 3 (4), 041303 10.1063/5.0092864.

[ref19] Gütlich P.; Hauser A. Thermal and Light-Induced Spin Crossover in Iron(II) Complexes. Coord. Chem. Rev. 1990, 97 (C), 1–22. 10.1016/0010-8545(90)80076-6.

[ref20] McCusker J. K. Electronic Structure in the Transition Metal Block and Its Implications for Light Harvesting. Science 2019, 363 (6426), 484–488. 10.1126/science.aav9104. 30705184

[ref21] Liu Y.; Persson P.; Sundström V.; Wärnmark K. Fe *N*-Heterocyclic Carbene Complexes as Promising Photosensitizers. Acc. Chem. Res. 2016, 49 (8), 1477–1485. 10.1021/acs.accounts.6b00186. 27455191

[ref22] Liu Y.; Harlang T.; Canton S. E.; Chábera P.; Suárez-Alcántara K.; Fleckhaus A.; Vithanage D. A.; Göransson E.; Corani A.; Lomoth R.; et al. Towards Longer-Lived Metal-to-Ligand Charge Transfer States of Iron(II) Complexes: An *N*-Heterocyclic Carbene Approach. Chem. Commun. 2013, 49 (57), 6412 10.1039/c3cc43833c. 23752944

[ref23] Harlang T. C. B.; Liu Y.; Gordivska O.; Fredin L. A.; Ponseca C. S.; Huang P.; Chábera P.; Kjaer K. S.; Mateos H.; Uhlig J.; et al. Iron Sensitizer Converts Light to Electrons with 92% Yield. Nat. Chem. 2015, 7 (11), 883–889. 10.1038/nchem.2365. 26492008

[ref24] Duchanois T.; Etienne T.; Cebrián C.; Liu L.; Monari A.; Beley M.; Assfeld X.; Haacke S.; Gros P. C. An Iron-Based Photosensitizer with Extended Excited-State Lifetime: Photophysical and Photovoltaic Properties. Eur. J. Inorg. Chem. 2015, 2015 (14), 2469–2477. 10.1002/ejic.201500142.

[ref25] Leshchev D.; Harlang T. C. B.; Fredin L. A.; Khakhulin D.; Liu Y.; Biasin E.; Laursen M. G.; Newby G. E.; Haldrup K.; Nielsen M. M.; et al. Tracking the Picosecond Deactivation Dynamics of a Photoexcited Iron Carbene Complex by Time-Resolved X-Ray Scattering. Chem. Sci. 2018, 9 (2), 405–414. 10.1039/C7SC02815F. 29629111 PMC5868308

[ref26] Chábera P.; Kjaer K. S.; Prakash O.; Honarfar A.; Liu Y.; Fredin L. A.; Harlang T. C. B.; Lidin S.; Uhlig J.; Sundström V.; et al. Fe^II^ Hexa *N*-Heterocyclic Carbene Complex with a 528 ps Metal-to-Ligand Charge-Transfer Excited-State Lifetime. J. Phys. Chem. Lett. 2018, 9 (3), 459–463. 10.1021/acs.jpclett.7b02962. 29298063

[ref27] Zimmer P.; Burkhardt L.; Friedrich A.; Steube J.; Neuba A.; Schepper R.; Müller P.; Flörke U.; Huber M.; Lochbrunner S.; Bauer M. The Connection between NHC Ligand Count and Photophysical Properties in Fe(II) Photosensitizers: An Experimental Study. Inorg. Chem. 2018, 57 (1), 360–373. 10.1021/acs.inorgchem.7b02624. 29236487

[ref28] Magra K.; Domenichini E.; Francés-Monerris A.; Cebrián C.; Beley M.; Darari M.; Pastore M.; Monari A.; Assfeld X.; Haacke S.; et al. Impact of the Fac/Mer Isomerism on the Excited-State Dynamics of Pyridyl-Carbene Fe(II) Complexes. Inorg. Chem. 2019, 58 (8), 5069–5081. 10.1021/acs.inorgchem.9b00138. 30950264

[ref29] Francés-Monerris A.; Magra K.; Darari M.; Cebrián C.; Beley M.; Domenichini E.; Haacke S.; Pastore M.; Assfeld X.; Gros P. C.; et al. Synthesis and Computational Study of a Pyridylcarbene Fe(II) Complex: Unexpected Effects of Fac/Mer Isomerism in Metal-to-Ligand Triplet Potential Energy Surfaces. Inorg. Chem. 2018, 57 (16), 10431–10441. 10.1021/acs.inorgchem.8b01695. 30063338

[ref30] Chábera P.; Liu Y.; Prakash O.; Thyrhaug E.; El Nahhas A.; Honarfar A.; Essén S.; Fredin L. A.; Harlang T. C. B.; Kjær K. S.; et al. A Low-Spin Fe(III) Complex with 100-ps Ligand-to-Metal Charge Transfer Photoluminescence. Nature 2017, 543 (7647), 695–699. 10.1038/nature21430. 28358064

[ref31] Kjær K. S.; Kaul N.; Prakash O.; Chábera P.; Rosemann N. W.; Honarfar A.; Gordivska O.; Fredin L. A.; Bergquist K.-E.; Häggström L.; et al. Luminescence and Reactivity of a Charge-Transfer Excited Iron Complex with Nanosecond Lifetime. Science 2019, 363 (6424), 249–253. 10.1126/science.aau7160. 30498167

[ref32] Braun J. D.; Lozada I. B.; Kolodziej C.; Burda C.; Newman K. M. E.; van Lierop J.; Davis R. L.; Herbert D. E. Iron(II) Coordination Complexes with Panchromatic Absorption and Nanosecond Charge-Transfer Excited State Lifetimes. Nat. Chem. 2019, 11 (12), 1144–1150. 10.1038/s41557-019-0357-z. 31740761

[ref33] Braun J. D.; Lozada I. B.; Herbert D. E. In Pursuit of Panchromatic Absorption in Metal Coordination Complexes: Experimental Delineation of the HOMO Inversion Model Using Pseudo-Octahedral Complexes of Diarylamido Ligands. Inorg. Chem. 2020, 59 (23), 17746–17757. 10.1021/acs.inorgchem.0c02973. 33225695

[ref34] Sinha N.; Wenger O. S. Photoactive Metal-to-Ligand Charge Transfer Excited States in 3d^6^ Complexes with Cr 0, Mn I, Fe II, and Co III. J. Am. Chem. Soc. 2023, 145 (9), 4903–4920. 10.1021/jacs.2c13432. 36808978 PMC9999427

[ref35] Kunnus K.; Vacher M.; Harlang T. C. B.; Kjær K. S.; Haldrup K.; Biasin E.; van Driel T. B.; Pápai M.; Chabera P.; Liu Y.; et al. Vibrational Wavepacket Dynamics in Fe Carbene Photosensitizer Determined with Femtosecond X-Ray Emission and Scattering. Nat. Commun. 2020, 11 (1), 634 10.1038/s41467-020-14468-w. 32005815 PMC6994595

[ref36] Tatsuno H.; Kjær K. S.; Kunnus K.; Harlang T. C. B.; Timm C.; Guo M.; Chàbera P.; Fredin L. A.; Hartsock R. W.; Reinhard M. E.; et al. Hot Branching Dynamics in a Light-Harvesting Iron Carbene Complex Revealed by Ultrafast X-ray Emission Spectroscopy. Angew. Chem., Int. Ed. 2020, 59 (1), 364–372. 10.1002/anie.201908065. 31602726

[ref37] Hainer F.; Alagna N.; Reddy Marri A.; Penfold T. J.; Gros P. C.; Haacke S.; Buckup T. Vibrational Coherence Spectroscopy Identifies Ultrafast Branching in an Iron(II) Sensitizer. J. Phys. Chem. Lett. 2021, 12 (35), 8560–8565. 10.1021/acs.jpclett.1c01580. 34468159

[ref38] Müller C.; Pascher T.; Eriksson A.; Chabera P.; Uhlig J. KiMoPack: A Python Package for Kinetic Modeling of the Chemical Mechanism. J. Phys. Chem. A 2022, 126 (25), 4087–4099. 10.1021/acs.jpca.2c00907. 35700393 PMC9251768

[ref39] Fredin L. A.; Wärnmark K.; Sundström V.; Persson P. Molecular and Interfacial Calculations of Iron(II) Light Harvesters. ChemSusChem 2016, 9 (7), 667–675. 10.1002/cssc.201600062. 27010851

[ref40] Malone R. A.; Kelley D. F. Interligand Electron Transfer and Transition State Dynamics in Ru(II)Trisbipyridine. J. Chem. Phys. 1991, 95 (12), 8970–8976. 10.1063/1.461228.

[ref41] Wallin S.; Davidsson J.; Modin J.; Hammarström L. Femtosecond Transient Absorption Anisotropy Study on [Ru(bpy)_3_]^2+^ and [Ru(bpy)(py)_4_]^2+^. Ultrafast Interligand Randomization of the MLCT State. J. Phys. Chem. A 2005, 109 (21), 4697–4704. 10.1021/jp0509212. 16833810

[ref42] van Stokkum I. H. M.; Larsen D. S.; van Grondelle R. Global and Target Analysis of Time-Resolved Spectra. Biochimica et Biophysica Acta (BBA) - Bioenergetics 2004, 1657 (2–3), 82–104. 10.1016/j.bbabio.2004.04.011. 15238266

[ref43] Gawelda W.; Cannizzo A.; Pham V.; van Mourik F.; Bressler C.; Chergui M. Ultrafast Nonadiabatic Dynamics of [Fe^II^(bpy)_3_]^2+^ in Solution. J. Am. Chem. Soc. 2007, 129 (26), 8199–8206. 10.1021/ja070454x. 17559211

[ref44] Liu L.; Agathangelou D.; Roland T.; Crégut O.; Duchanois T.; Beley M.; Léonard J.; Gros P.; Haacke S. High Sensitivity Fluorescence Up-Conversion Spectroscopy of ^3^MLCT Emission of Metal-Organic Complexes. EPJ. Web Conf. 2019, 205, 09009 10.1051/epjconf/201920509009.

[ref45] Kjær K. S.; Kunnus K.; Harlang T. C. B.; Van Driel T. B.; Ledbetter K.; Hartsock R. W.; Reinhard M. E.; Koroidov S.; Li L.; Laursen M. G.; et al. Solvent Control of Charge Transfer Excited State Relaxation Pathways in [Fe(2,2′-Bipyridine)(CN)_4_]^2–^. Phys. Chem. Chem. Phys. 2018, 20 (6), 4238–4249. 10.1039/C7CP07838B. 29364300

[ref46] Mengel A. K. C.; Förster C.; Breivogel A.; Mack K.; Ochsmann J. R.; Laquai F.; Ksenofontov V.; Heinze K. A Heteroleptic Push-Pull Substituted Iron(II) Bis(Tridentate) Complex with Low-Energy Charge-Transfer States. Chem.—Eur. J. 2015, 21 (2), 704–714. 10.1002/chem.201404955. 25393132

[ref47] Schmid L.; Chábera P.; Rüter I.; Prescimone A.; Meyer F.; Yartsev A.; Persson P.; Wenger O. S. Borylation in the Second Coordination Sphere of Fe^II^ Cyanido Complexes and Its Impact on Their Electronic Structures and Excited-State Dynamics. Inorg. Chem. 2022, 61 (40), 15853–15863. 10.1021/acs.inorgchem.2c01667. 36167335 PMC9554916

[ref48] Darari M.; Francés-Monerris A.; Marekha B.; Doudouh A.; Wenger E.; Monari A.; Haacke S.; Gros P. C. Towards Iron(II) Complexes with Octahedral Geometry: Synthesis, Structure and Photophysical Properties. Molecules 2020, 25 (24), 5991 10.3390/molecules25245991. 33348914 PMC7767130

[ref49] Sun Q.; Mosquera-Vazquez S.; Lawson Daku L. M.; Guénée L.; Goodwin H. A.; Vauthey E.; Hauser A. Experimental Evidence of Ultrafast Quenching of the ^3^MLCT Luminescence in Ruthenium(II) Tris-Bipyridyl Complexes via a ^3^dd State. J. Am. Chem. Soc. 2013, 135 (37), 13660–13663. 10.1021/ja407225t. 24000998

[ref50] Cadranel A.; Pieslinger G. E.; Tongying P.; Kuno M. K.; Baraldo L. M.; Hodak J. H. Spectroscopic Signatures of Ligand Field States in {Ru II (Imine)} Complexes. Dalton Transactions 2016, 45 (13), 5464–5475. 10.1039/C5DT04332H. 26841245

[ref51] Soupart A.; Alary F.; Heully J.-L.; Elliott P. I. P.; Dixon I. M. Recent Progress in Ligand Photorelease Reaction Mechanisms: Theoretical Insights Focusing on Ru(II) ^3^MC States. Coord. Chem. Rev. 2020, 408, 213184 10.1016/j.ccr.2020.213184.

[ref52] Österman T.; Abrahamsson M.; Becker H.-C.; Hammarström L.; Persson P. Influence of Triplet State Multidimensionality on Excited State Lifetimes of Bis-Tridentate Ru^II^ Complexes: A Computational Study. J. Phys. Chem. A 2012, 116 (3), 1041–1050. 10.1021/jp207044a. 22148266

[ref53] Jacquemin D.; Escudero D. The Short Device Lifetimes of Blue PhOLEDs: Insights into the Photostability of Blue Ir(III) Complexes. Chem. Sci. 2017, 8 (11), 7844–7850. 10.1039/C7SC03905K. 29163921 PMC5674210

[ref54] Rojas Pérez Y.; Slep L. D.; Etchenique R. Cis – Trans Interconversion in Ruthenium(II) Bipyridine Complexes. Inorg. Chem. 2019, 58 (17), 11606–11613. 10.1021/acs.inorgchem.9b01485. 31418260

[ref55] Escudero D. Mer-Ir(ppy)_3_ to Fac-Ir(ppy)_3_ Photoisomerization. ChemPhotoChem. 2019, 3 (9), 697–701. 10.1002/cptc.201900029.

[ref56] Feng L.; Wang Y.; Jia J. Triplet Ground-State-Bridged Photochemical Process: Understanding the Photoinduced Chiral Inversion at the Metal Center of [Ru(phen)_2_(L-ser)]^+^ and Its Bipy Analogues. Inorg. Chem. 2017, 56 (23), 14467–14476. 10.1021/acs.inorgchem.7b02030. 29130683

[ref57] Göttle A. J.; Dixon I. M.; Alary F.; Heully J.-L.; Boggio-Pasqua M. Adiabatic Versus Nonadiabatic Photoisomerization in Photochromic Ruthenium Sulfoxide Complexes: A Mechanistic Picture from Density Functional Theory Calculations. J. Am. Chem. Soc. 2011, 133 (24), 9172–9174. 10.1021/ja201625b. 21604806

[ref58] Arroliga-Rocha S.; Escudero D. Facial and Meridional Isomers of Tris(Bidentate) Ir(III) Complexes: Unravelling Their Different Excited State Reactivity. Inorg. Chem. 2018, 57 (19), 12106–12112. 10.1021/acs.inorgchem.8b01675. 30222324

[ref59] Fredin L. A.; Pápai M.; Rozsályi E.; Vankó G.; Wärnmark K.; Sundström V.; Persson P. Exceptional Excited-State Lifetime of an Iron(II)–*N*-Heterocyclic Carbene Complex Explained. J. Phys. Chem. Lett. 2014, 5 (12), 2066–2071. 10.1021/jz500829w. 26270494

[ref60] Kasha M. Characterization of Electronic Transitions in Complex Molecules. Discuss. Faraday Soc. 1950, 9 (c), 14–19. 10.1039/df9500900014.

[ref61] Housecroft C.; Sharpe A. G. Inorganic Chemistry, 5th ed.; Pearson Education: New York, 2018.

[ref62] Monat J. E.; McCusker J. K. Femtosecond Excited-State Dynamics of an Iron(II) Polypyridyl Solar Cell Sensitizer Model. J. Am. Chem. Soc. 2000, 122 (17), 4092–4097. 10.1021/ja992436o.

[ref63] Carey M. C.; Adelman S. L.; McCusker J. K. Insights into the Excited State Dynamics of Fe(II) Polypyridyl Complexes from Variable-Temperature Ultrafast Spectroscopy. Chem. Sci. 2019, 10 (1), 134–144. 10.1039/C8SC04025G. 30746076 PMC6335846

[ref64] McCusker J. K.; Walda K. N.; Dunn R. C.; Simon J. D.; Magde D.; Hendrickson D. N. Subpicosecond ^1^MLCT → ^5^T_2_ Intersystem Crossing of Low-Spin Polypyridyl Ferrous Complexes. J. Am. Chem. Soc. 1993, 115 (4), 298–307. 10.1021/ja00054a043.

[ref65] Chergui M. Ultrafast Photophysics of Transition Metal Complexes. Acc. Chem. Res. 2015, 48 (3), 801–808. 10.1021/ar500358q. 25646968

[ref66] Oppermann M.; Zinna F.; Lacour J.; Chergui M. Chiral Control of Spin-Crossover Dynamics in Fe(II) Complexes. Nat. Chem. 2022, 14 (7), 739–745. 10.1038/s41557-022-00933-0. 35618767

[ref67] Consani C.; Prémont-Schwarz M.; ElNahhas A.; Bressler C.; van Mourik F.; Cannizzo A.; Chergui M. Vibrational Coherences and Relaxation in the High-Spin State of Aqueous [Fe^II^(bpy)_3_]^2+^. Angew. Chem., Int. Ed. 2009, 48 (39), 7184–7187. 10.1002/anie.200902728. 19718731

[ref68] Vankó G.; Bordage A.; Pápai M.; Haldrup K.; Glatzel P.; March A. M.; Doumy G.; Britz A.; Galler A.; Assefa T.; et al. Detailed Characterization of a Nanosecond-Lived Excited State: X-Ray and Theoretical Investigation of the Quintet State in Photoexcited [Fe(terpy)_2_]^2+^. J. Phys. Chem. C 2015, 119 (11), 5888–5902. 10.1021/acs.jpcc.5b00557. PMC4368081 25838847

[ref69] Pápai M.; Penfold T. J.; Mo̷ller K. B. Effect of *tert*-Butyl Functionalization on the Photoexcited Decay of a Fe(II)-*N*-Heterocyclic Carbene Complex. J. Phys. Chem. C 2016, 120 (31), 17234–17241. 10.1021/acs.jpcc.6b05023.

[ref70] Pápai M.; Vankó G.; Rozgonyi T.; Penfold T. J. High-Efficiency Iron Photosensitizer Explained with Quantum Wavepacket Dynamics. J. Phys. Chem. Lett. 2016, 7 (11), 2009–2014. 10.1021/acs.jpclett.6b00711. 27187868

[ref71] Pápai M.; Abedi M.; Levi G.; Biasin E.; Nielsen M. M.; Mo̷ller K. B. Theoretical Evidence of Solvent-Mediated Excited-State Dynamics in a Functionalized Iron Sensitizer. J. Phys. Chem. C 2019, 123 (4), 2056–2065. 10.1021/acs.jpcc.8b10768.

[ref72] Larsen C. B.; Braun J. D.; Lozada I. B.; Kunnus K.; Biasin E.; Kolodziej C.; Burda C.; Cordones A. A.; Gaffney K. J.; Herbert D. E. Reduction of Electron Repulsion in Highly Covalent Fe-Amido Complexes Counteracts the Impact of a Weak Ligand Field on Excited-State Ordering. J. Am. Chem. Soc. 2021, 143, 20645–20656. 10.1021/jacs.1c06429. 34851636

[ref73] Paulus B. C.; Adelman S. L.; Jamula L. L.; McCusker J. K. Leveraging Excited-State Coherence for Synthetic Control of Ultrafast Dynamics. Nature 2020, 582 (7811), 214–218. 10.1038/s41586-020-2353-2. 32528090

[ref74] Kjær K. S.; Van Driel T. B.; Harlang T. C. B.; Kunnus K.; Biasin E.; Ledbetter K.; Hartsock R. W.; Reinhard M. E.; Koroidov S.; Li L.; Laursen M. G.; et al. Finding Intersections between Electronic Excited State Potential Energy Surfaces with Simultaneous Ultrafast X-Ray Scattering and Spectroscopy. Chem. Sci. 2019, 10 (22), 5749–5760. 10.1039/C8SC04023K. 31293761 PMC6568243

[ref75] Britz A.; Gawelda W.; Assefa T. A.; Jamula L. L.; Yarranton J. T.; Galler A.; Khakhulin D.; Diez M.; Harder M.; Doumy G.; et al. Using Ultrafast X-Ray Spectroscopy To Address Questions in Ligand-Field Theory: The Excited State Spin and Structure of [Fe(dcpp)_2_]^2+^. Inorg. Chem. 2019, 58 (14), 9341–9350. 10.1021/acs.inorgchem.9b01063. 31241335

