# Supplementary material for: Multifaceted Deactivation Dynamics of Fe(II) N-Heterocyclic Carbene Photosensitizers
Source: J Phys Chem A. 2023 Nov 24;127(48):10210–22. doi: 10.1021/acs.jpca.3c06983 (PMC10711794; doi:10.1021/acs.jpca.3c06983)
Supplement: Supplementary file 1 — jp3c06983_si_001.pdf [file jp3c06983_si_001.pdf]

Multifaceted Deactivation Dynamics of Fe(II) N-heterocyclic Carbene Photosensitizers

Linnea Lindh[a,b], Torbjörn Pascher[a], Samuel Persson[c], Yogesh Goriya[c], Kenneth Wärnmark[c], Jens Uhlig[a], Pavel Chábera[a], Petter Persson[b]* and Arkady Yartsev[a]*

[a] L. Lindh, Dr. T. Pascher, Dr. P. Chábera, Prof. J. Uhlig, Prof. A. Yartsev
Department of Chemical Physics, Lund University, Box 124, SE-22100 Lund, Sweden
E-mail: arkady.yartsev@chemphys.lu.se

[b] L. Lindh, Prof. P. Persson
Theoretical Chemistry Department, Chemistry Department, Lund University, Box 124, SE-22100 Lund, Sweden
E-mail: petter.persson@teokem.lu.se

[c] S. Persson, Dr. Y. Goriya, Dr. Prof. K. Wärnmark
Centre for Analysis and Synthesis, Department of Chemistry, Lund University, Box 124, SE-22100 Lund, Sweden

Table of Contents

1. Artefact evaluation 1
2. UV-TA data 4
3. Vis-TA data 5
4. GS cooling fits 9
5. Oscillations 10
6. Target analysis 11
7. Anisotropy 32
8. Temperature dependent TA fits 34
9. Stability 37
10. References 38

1. Artefact evaluation

We have utilized thin cuvettes (100 um path length) with ultrathin windows (200 um). With that, we have drastically reduced the contribution of the cuvette and solvent ("solvent response") to the solute response both in amplitude and in time. The solvent response (i.e. the transient absorption (TA) signal of the thin cuvette with solvent) is shown in Figure SI.1. Note that the strongest signals have a peak amplitude of ~0.1 mOD. The weakest TA signals displayed in this manuscript is on the order of ~0.5 mOD at the early peak even though attenuation of the excitation due to absorption of the solute is not taken into account for these measurements. In the red part of the TA spectrum (550-800 nm) where resolving the ultrafast decay component is crucial for this study, the solvent response is nearly twice weaker than in the blue, see Figure SI.2. Therefore, we state that the ultrafast dynamics of the red excited state absorption (ESA) in complex **1** is due to processes in the solute, not artefacts caused by the solvent or cuvette.

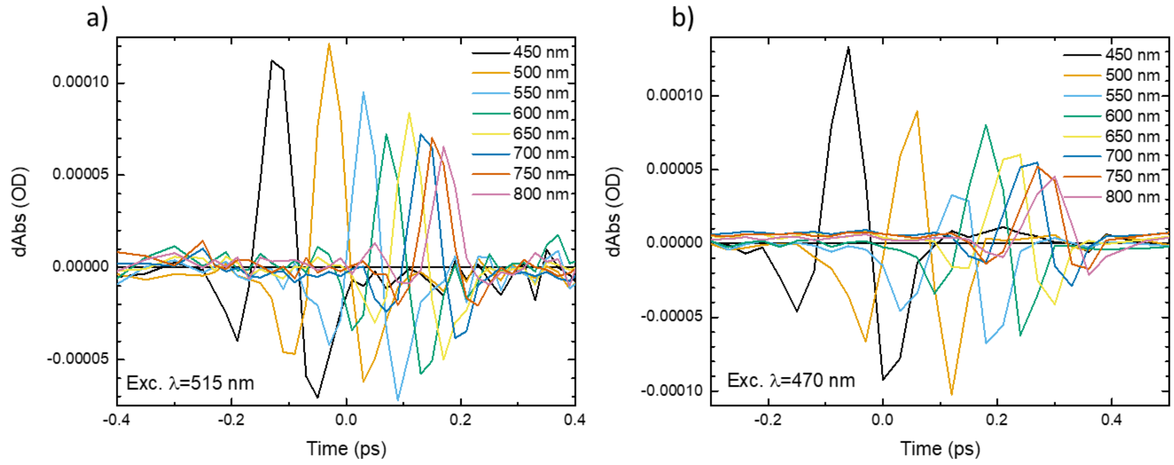

*Figure SI.1. Solvent response dynamics measured at identical conditions as the measured TA data of a) the acidic buffer in the thin cuvette used in the measurements of complex **3** and b) acetonitrile in the thin cuvette used in the measurements of complexes **1** and **2**.*

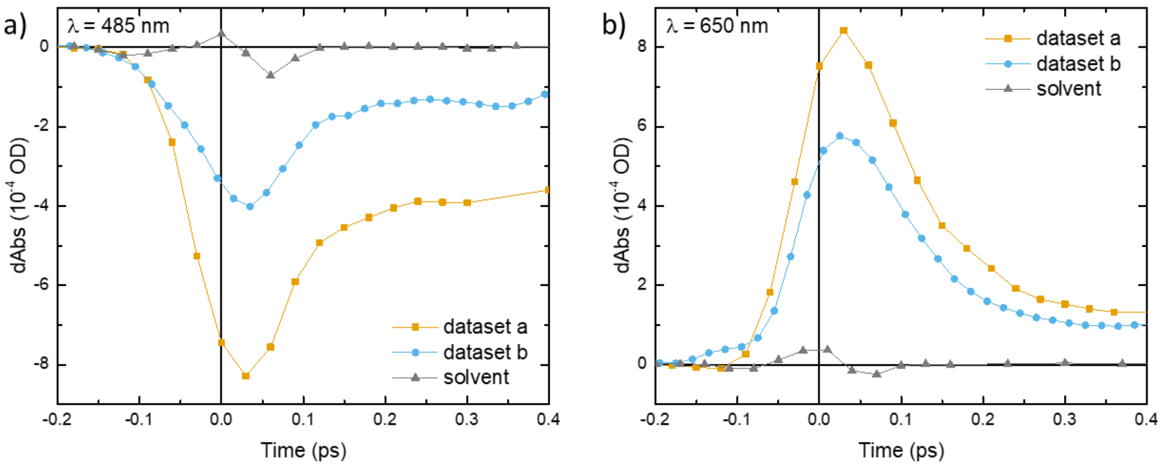

*Figure SI.2. Solvent response compared with the TA signal for complex **1** in acetonitrile at a) 485 nm and b) 650 nm. The two graphs show two different datasets where the concentration of **1** was slightly different. The solvent response was scaled to account for similar pump light absorption as in the low concentration sample.*

All TA data shown was measured with a perpendicular polarisation between pump and probe beams with a Glan-Thompson polarizer in the probe set to block scattering of the pump beam. As a consequence of this, we avoid pump scatter at the excitation wavelength (470 nm, 495 nm resp. 515 nm), but instead in the time-resolved spectra we see a contribution of the pump spectrum in the pump-probe pulse overlap. We associate this signal to the probe-induced Kerr effect for the pump beam in the sample (see Figure SI.3), which however does not affect the conclusions we make regarding the ESA evolution.

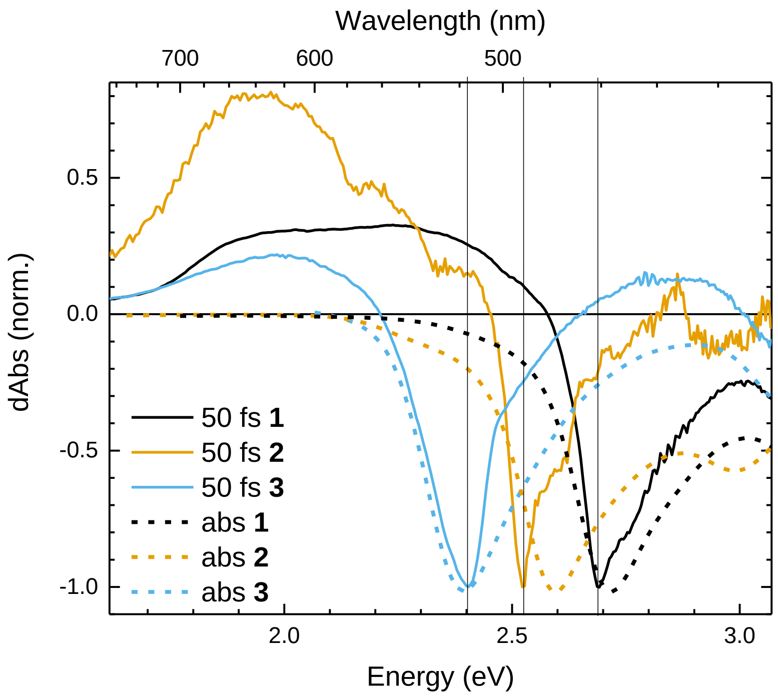

Figure SI.3. Normalized TA spectra at 50 fs delay time for all investigated complexes showing the distorted ground state bleach (GSB) spectra by the pump spectrum at the wavelength indicated by vertical lines. This is assigned to the Kerr effect. The contribution of this signal has been cut in the data shown in the main manuscript. Steady state absorption of each complex is shown by the dashed lines.

No decay associated spectra (DAS) for the component faster than the instrument response function (IRF) is shown in the main manuscript, as the shape of any component on these short timescales could be substantially influenced by the uncertainty in the IRF. In Figure SI.4, we show a comparison between the fastest DAS and the TA spectra measured at 50 fs delay in the ESA wavelength range as this is the range where we have established the contribution of this component.

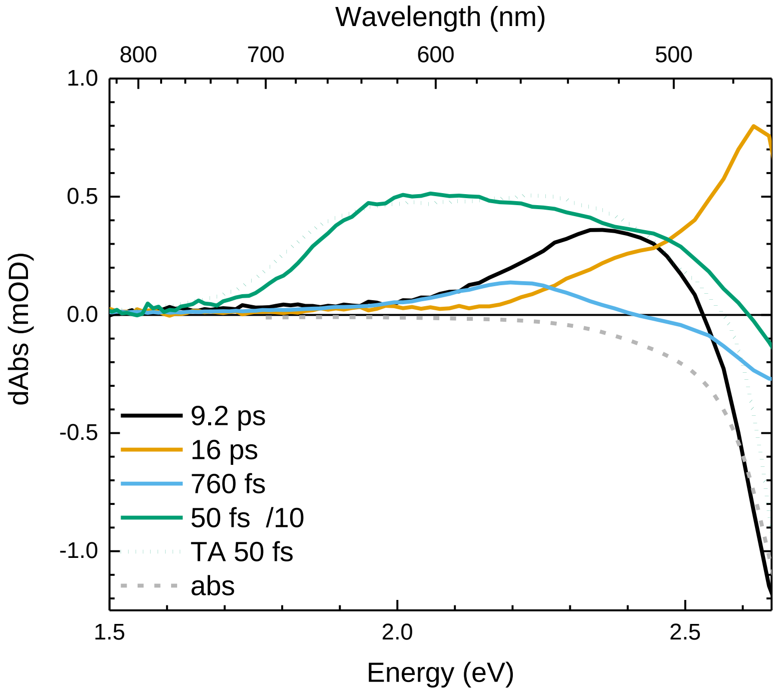

*Figure SI.4. Fitted decay associated spectra of complex **1**. The decay associated spectra with time component 50 fs is compared to the TA spectra measured at 50 fs delay time.*

2. UV-TA data

Here we present the measured TA data of complex **1** and **2** in the UV spectral range (280-380 nm). The TA data was fitted by global analysis using available software.[1] To avoid the early time artifact contribution, the data was cut before 1 ps and the instrument response function (IRF) was set as 90 fs. For TA data in the UV range of complex **3**, we refer the reader to the publication by Duchanois et al..[2]

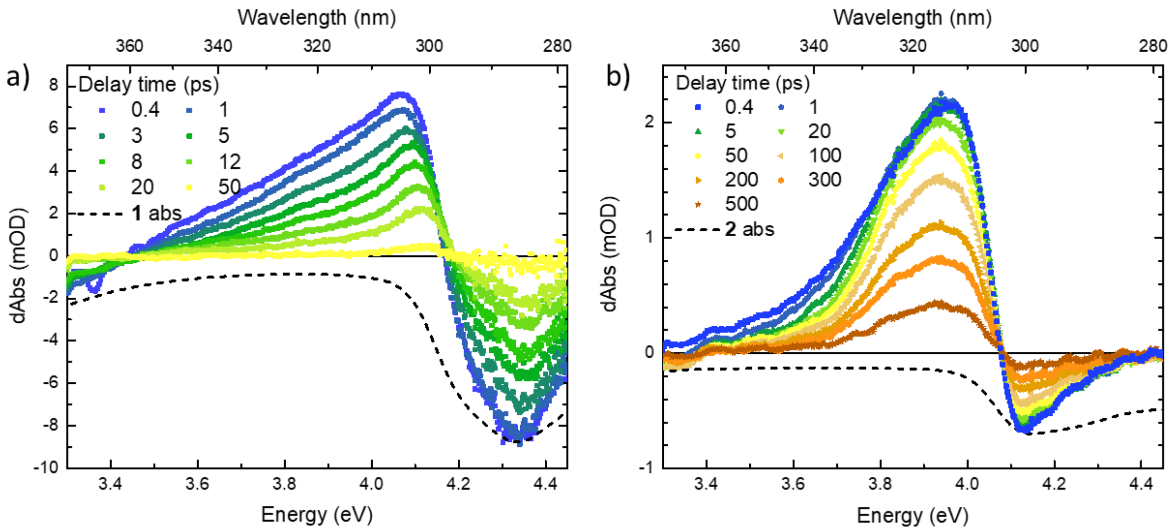

*Figure SI.5. TA spectra of a) **1** and b) **2** in the UV wavelength range. Spectra are chirp- and background corrected. The (inverted) linear absorption spectra of each molecule are included for comparison.*

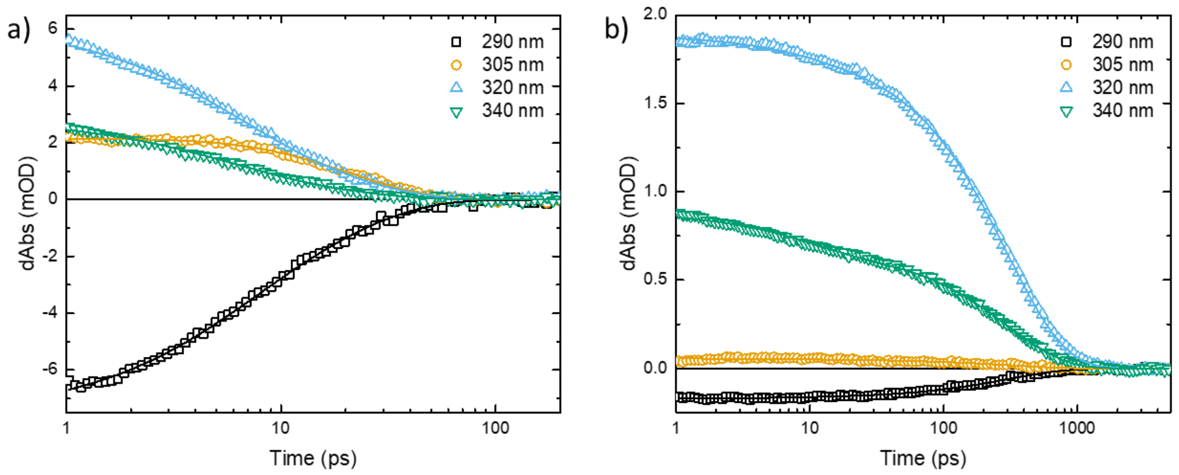

*Figure SI.6. TA kinetics of a) **1** and b) **2** in the UV wavelength range. TA data is chirp- and background corrected. Fits are represented by the solid lines.*

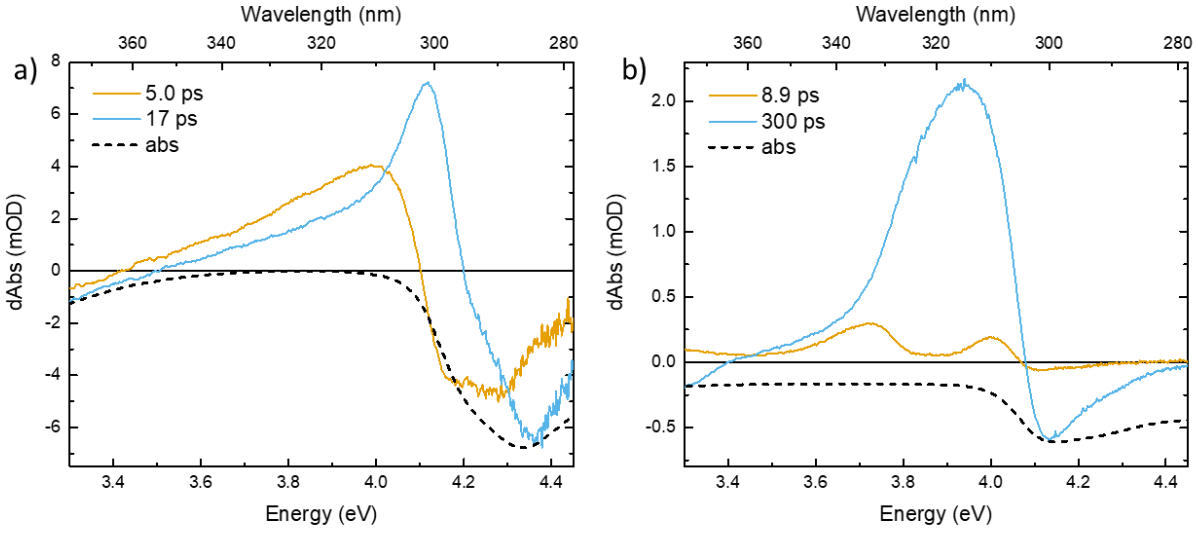

Figure SI.7. Decay associated spectra of TA data of a) ***1*** *and b)* ***2*** *in the UV wavelength range. The (inverted) linear absorption spectra of each molecule are included for comparison.*

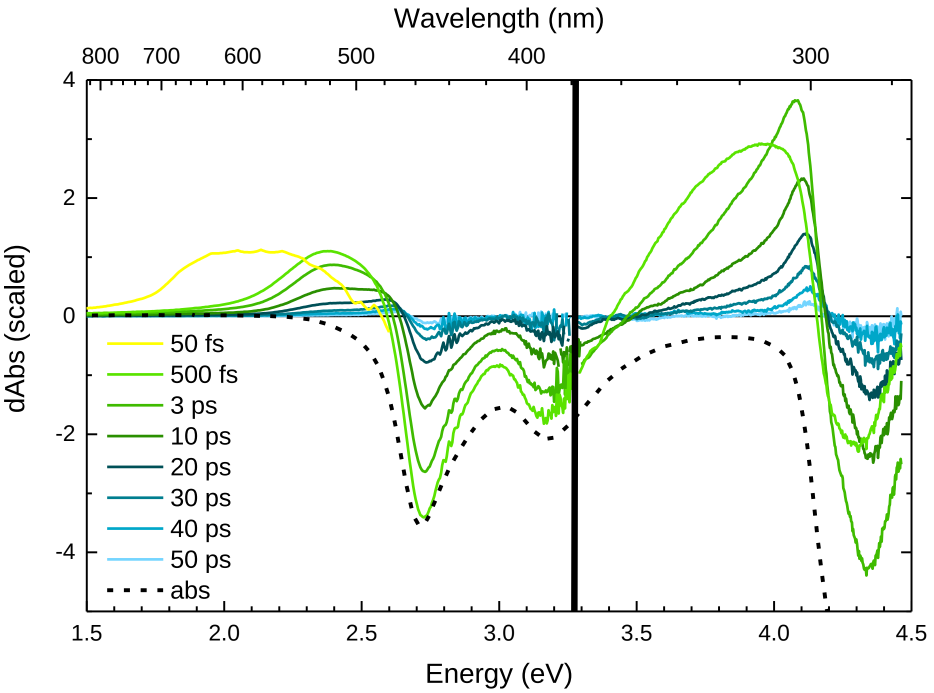

Figure SI.8. TA spectra of ***1*** *in the UV to NIR wavelength range. The relative scale of the UV and vis-NIR TA data is arbitrary, set by the authors. The black line indicates the transition between the two datasets, the dotted black line - the inverted steady-state absorption. TA spectrum at 50 fs delay time is only shown in the red wavelength range.*

3. Vis-TA data

To further understand what processes return population to the ground state in both molecules, single kinetics from the GSB bands were fitted by a sum of exponential functions. The fitting was conducted from ~100 fs or later to exclude rise components. For **1**, TA dynamics in the GSB region

requires a multiexponential fit to be described well, resulting in the components 0.5-1.3 ps, 6-10 ps and 10-24 ps (see Figure SI.9 and Table SI.1). The two longer components are dominating in different wavelength regions: 6-10 ps more towards blue (400-420 nm) and 10-24 ps more towards red (430-480 nm). Overall, the single kinetic fitting agrees with the DAS where the ~9 ps component has an amplitude in the whole GSB region, but the 16 ps component vanishes ~410 nm to then reappear again at shorter wavelengths. For **3**, the picture is simpler. The ground state recovery of **3** can be fitted with only one decay component, that is 18-23 ps (see Figure SI.10 and Table SI.2). A second component would be needed to fit the whole kinetic from 100 fs, but this process corresponds to a rise associated with the 190 fs component from global analysis.

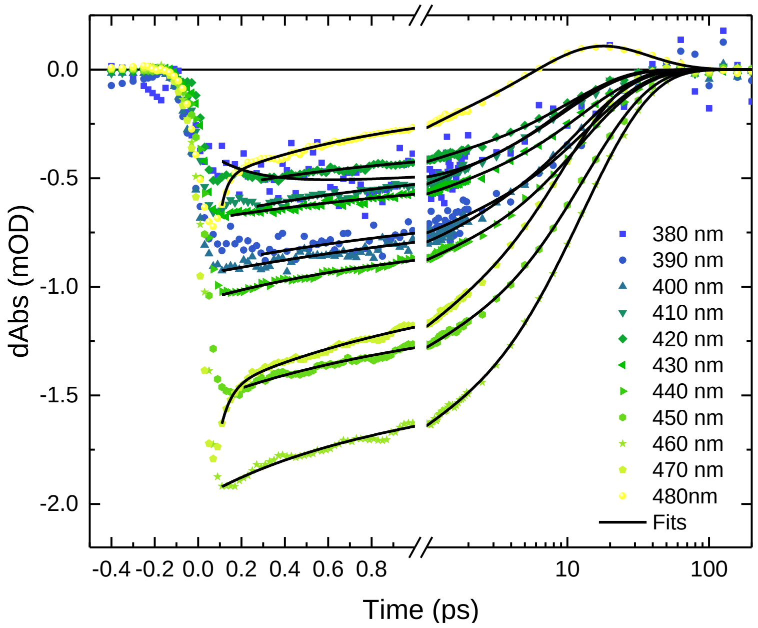

*Figure SI.9. GSB kinetics of **1** in the visible wavelength range. The black lines represent the fits that are also shown in the Table below.*

*Table SI.1. Fit components for the single kinetic in the GSB region of **1**.*

Wavelength (nm)	Comp (fs)	Comp (ps)	Comp (ps)
380		6	10
390	1000		14
400	1300	10	
410	890	9,5	
420	710	9,8	
430	1000		12
440	730	9,1	16
450	670-710	(6,6)	14-15
460	530	6,5	16
470*	720-740	9,3-9,5	(4,5)

480*	600	6,8 (rise)	24

*A decay component shorter than IRF is also needed for the fit here. This could be due to the Kerr effect, as these wavelengths are close to the excitation wavelength 470 nm. It could also be the rise of ESA features.

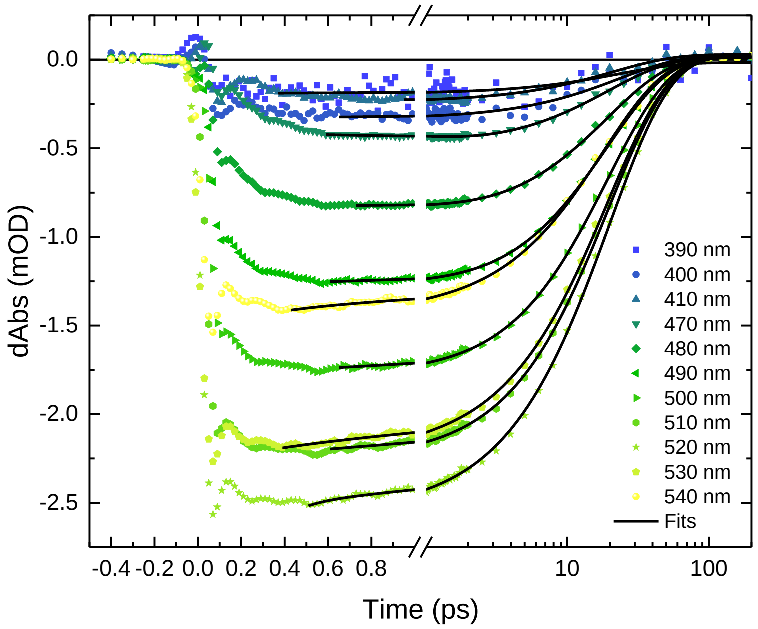

*Figure SI.10. GSB kinetics of **3** in the visible wavelength range. The black lines represent the fits that are also shown in the Table below.*

*Table SI.2. Fit components for the single kinetic in the GSB region of **3**.*

**Wavelength (nm)**	**Comp (ps)**	**Comp (ps)**
390		23
400		23
410		21
470		20
480		19
490		19
500		19
510		20
520	0.1	20
530	0.3	19
540	0.2	18

In Figure SI.11 we show for clarity the ESA wavelength range of the earliest measured TA spectra of complex **1**. The ESA B feature is first clearly seen at 100 fs delay time, when ESA feature A has decayed to some extent. The ESA B feature then shifts from 540 nm (at 100 fs) to 520 nm (at 1 ps).

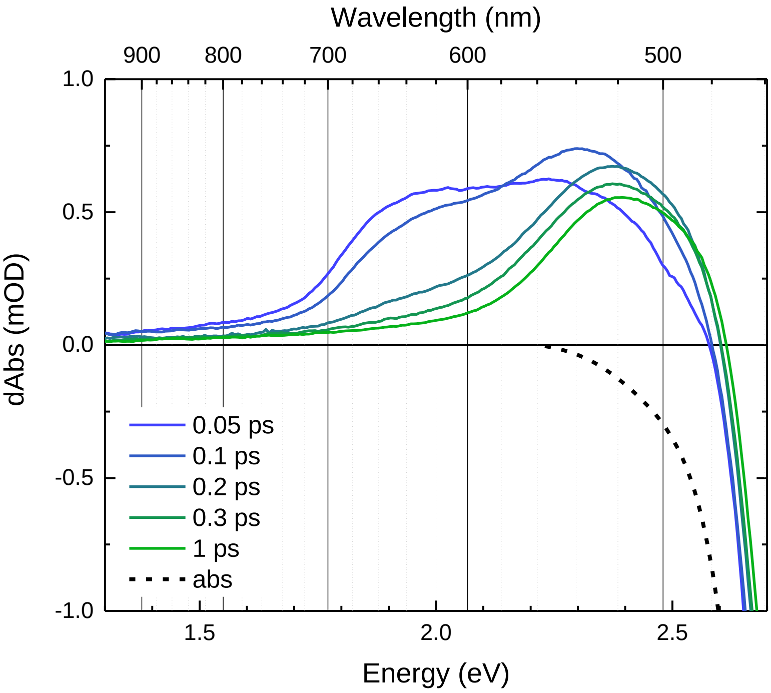

*Figure SI.11. Early TA spectral evolution of complex **1**. The plot is displaying the ESA wavelength range in order to follow the shift of spectral feature B. Spectra are chirp- and background corrected. The inverted steady-state absorption is indicated by the dashed black line.*

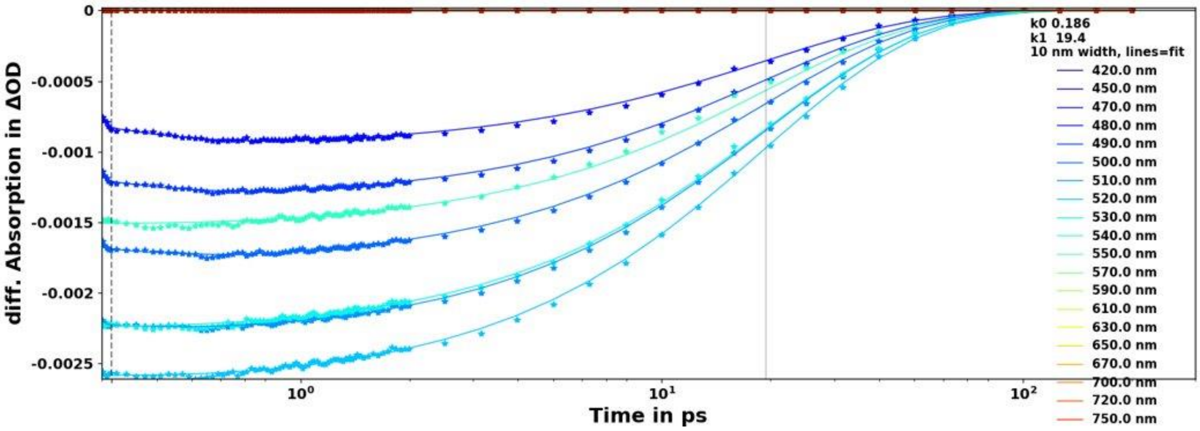

Figure SI.12. Global fit with two components in the GSB region (480-540 nm) of 3. Data was chirp- and background corrected, fit performed from 300 fs.

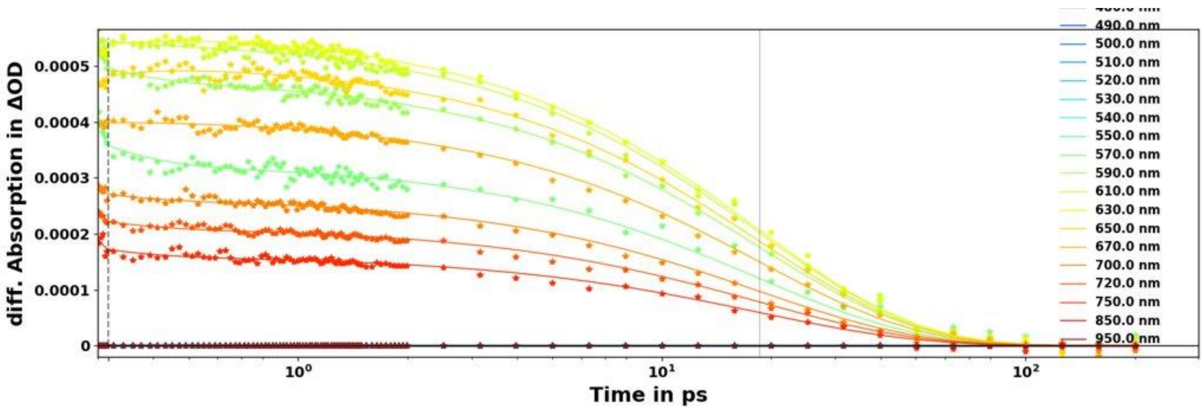

Figure SI.13. Global fit with two components in the ESA region (570-800 nm) of 3. Data was chirp- and background corrected, fit performed from 300 fs.

4. GS cooling fits

The spectral shape of the feature that correspond to GS cooling in the TA data should consist of a combination of negative steady-state absorption and a broadened (i.e. hot), positive ground state absorption. Here we present modelling of the TA spectra of **1** at late (≥30 ps) delay times, where this component should be prominent whereas less than 3% of the faster components should remain. As a first approximation, the lowest energy peak of the GSB band was fitted by a gaussian function. This negative gaussian function was summed up with a positive broader gaussian with the same area, to approximate the ground state absorption. The TA spectrum around the red GSB edge at different spectral ranges and delay times were fitted by this sum, see Figures SI.14-15. Different fit results can be found depending on what wavelength range is included in the fit, see Figure SI.14. None of the fits reproduce the full spectral shape satisfactorily.

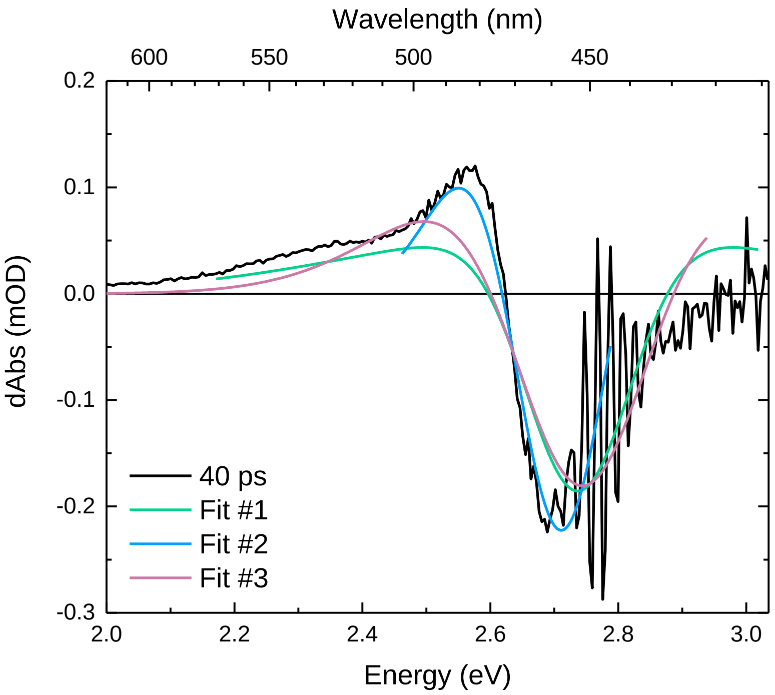

*Figure SI.14. TA spectra of **1** at 40 ps delay time, fitted with the GS cooling model. Here three different cases are compared, where the data has been cut at different places.*

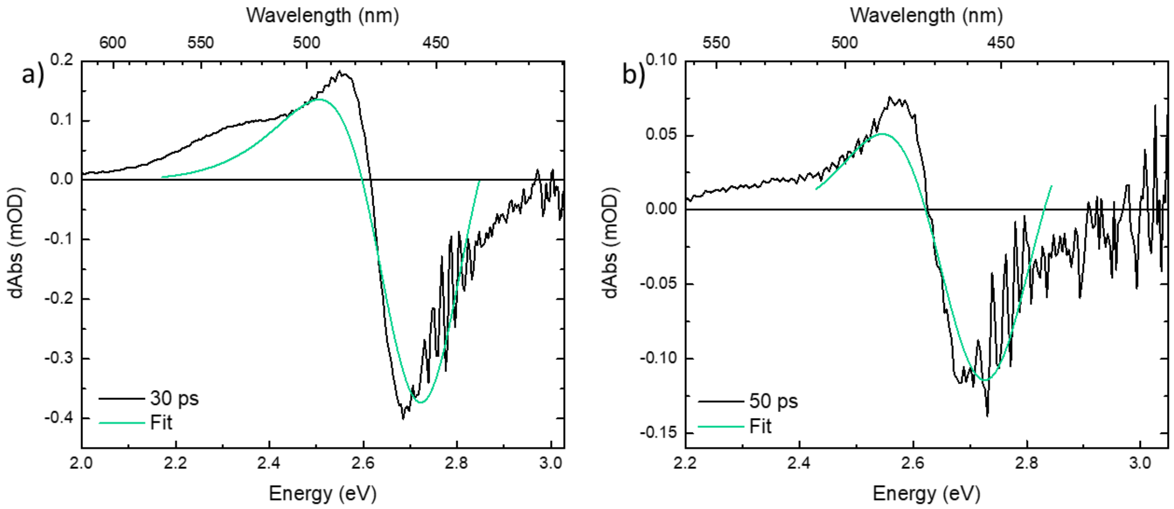

*Figure SI.15. TA spectra of **1** at a) 30 ps delay time and b) 50 ps delay time, fitted with the GS cooling model.*

5. Oscillations

The TA datasets of all complexes feature oscillations, mainly in the ESA wavelength range. Here we show a fit of the oscillations at the kinetics where they are the most prominent. Oscillations are identified by looking for oscillatory features in the residuals of the fit. The number of oscillatory components added was determined until the residuals were random (see Figures SI.16-18). In Table SI.3, the parameters of the fitted oscillations are collected.

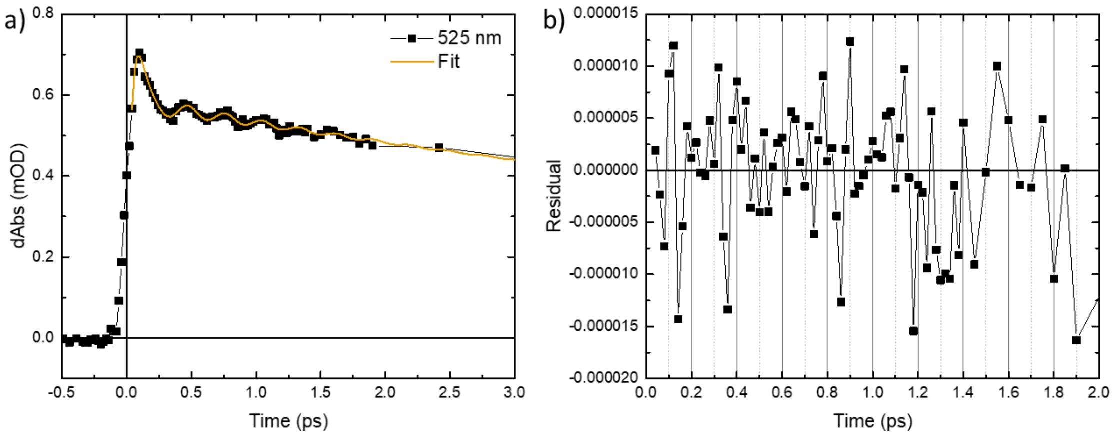

*Figure SI.16. a) TA kinetic at 525 nm of **1**, selected for having strong oscillations, with fit. b) Residuals after the fit.*

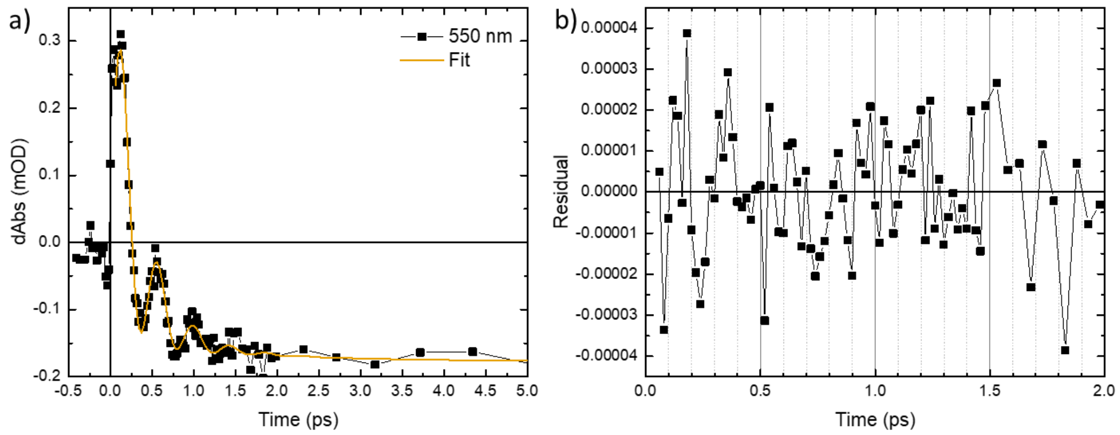

*Figure SI.17. a) TA kinetic at 550 nm of **2**, selected for having strong oscillations, with fit. b) Residuals after the fit.*

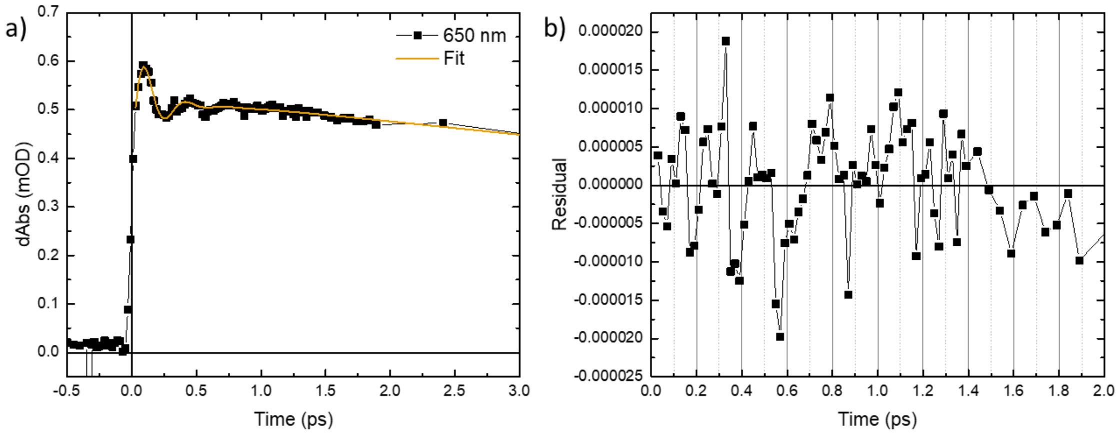

*Figure SI.18. a) TA kinetic at 650 nm of **3**, selected for having strong oscillations, with fit. b) Residuals after the fit.*

Table SI.3. Table concluding the oscillation fits.

Complex	Amplitude	Period time (fs)	Damping time (fs)	Phase (rad)
**1**	2.5E-5 / 7.9E-4	290 / 370	940 / 60	2.1 / 4.0
**2**	2.5E-4	430	380	3.8
**3**	1.7E-4	320	140	4.1

6. Target analysis

The different models considered in the target analysis are depicted here, together with their names and naming of the fitted states and decay rates. “br” stands for branching and is the faction of population that would go into the indicated state, defined so that the total population in the system is always 1. The TA data is fitted starting from 300 fs, to ensure that we only quantify processes that are slower than IRF in our experiment. The evaluation of the applicability of a model is based on the quality of the fit, randomness of the residuals and interpretability of the species associated spectra (SAS).

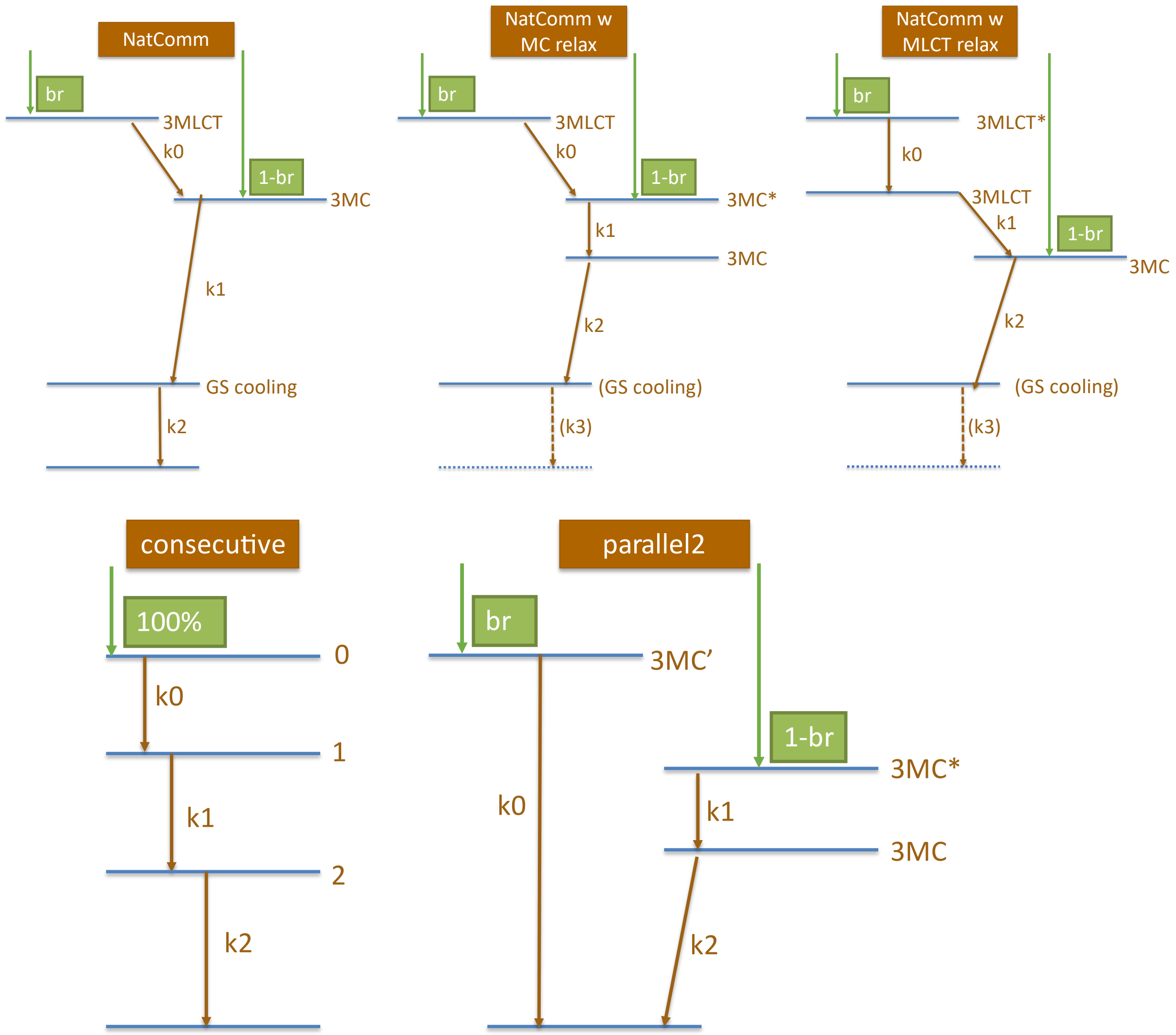

Figure SI.19. The different models analysed by target analysis.

To further quantify the disagreement of the model in Kunnus et al.[4] with our slow timescale data we applied target analysis using their model as the target. Since the branching between the ^{3}MLCT and the ^{3}MC states proposed in their paper is largely over at 300 fs, we introduce the branching ratio in the analysis as a varied parameter. First, we applied the model "NatComm" (see Figure SI.19) without any change with locked ^{3}MLCT lifetime of 9 ps, ^{3}MC lifetime of 1.5 ps and branching ratio of 60:40.[4] This model failed to fit the data due to non-random residuals and we conclude that our data disagree with the previously proposed model (see Figure SI.20). Next, we consider what modifications of the model can be made to tune it to fit the data. First, with freely varied decay times the fit naturally results in a substantial decrease of errors but the lifetimes changed to 610 fs, 13 ps and 20 ps. Furthermore, in the ESA wavelength range the resulting SAS exhibits a negative feature, which is an unphysical result as no species could have a contribution of a negative sign in this spectral region (see Figure SI.22). We then further adapted the model by adding one cooling/relaxation step in either the ^{3}MLCT or the ^{3}MC state (see Figure SI.19). For all attempts it was however still hard to interpret the fitted SAS in a physically meaningful way (see Figures SI.20-31). The best possible fitting required drastic variation of the branching between the ^{3}MLCT and the ^{3}MC with >75% of the population going to ^{3}MLCT, together with locking the lifetimes to the values from Kunnus et al.[4] (see Figure SI.27).

Based on the assignment discussed in the main manuscript that complex **1** first undergoes ultrafast MLCT->MC conversion (where target analysis would not be reliable due to the IRF response), two models for the subsequent dynamics were found to yield results that met all evaluation criteria. First, a purely consecutive model ("consecutive" Figure SI.19) was tested and found to fit the data with three decay components of 630 fs, 9 ps and 17 ps. This consecutive model only produces meaningful SAS when the rates are ordered from fastest to slowest process (see Figure SI.32-34). Furthermore, the similarity of the 630 fs and 9 ps SAS strongly suggests that both these decay rates are associated with the same state. The SAS belonging to the 17 ps lifetime instead shows signatures of having a separate origin. Summarizing the connection of the spectral features with the different steps in this consecutive model yields the following description of the dynamics as illustrated in the Figure SI.19 "consecutive". The initially populated 3MLCT state characterized by broad ESA in the red quickly (within IRF) converts into a hot ^{3}MC state with the spectral characteristics of feature B. This state undergoes sub-ps cooling (630 fs), and then decays on 9 ps to a second ^{3}MC state (3MC'), before this second 3MC state finally decays back to the 1GS on a 17 ps timescale.

We were also able to fit the TA with a parallel decay scheme containing two independent states between which the population is initially split, and with one of the states featuring a relaxation/cooling process such that this model also contained three separate rates (see Figure SI.19 "parallel2"). This model yielded time components 610 fs, 13 ps and 20 ps, see associated SAS in Figure SI.35-37. It is noteworthy that the similarity of these rates with those from the consecutive model discussed above suggest that the TA data clearly require these characteristic time components to be present even if different detailed deactivation schemes can be used to fit the data with similar accuracy. This also meant that testing revealed that there were some residual ambiguities both in the initial branching ratio and such that the target analysis could not distinguish whether the 610 fs relaxation/cooling should be associated with the 13 ps or 20 ps state meaning that the 610 fs SAS changes shape according to the state that follows in the kinetic model (see Figure SI.36 and SI.37). The branching ratio between the different states is coupled to the relative amplitudes of the decaying components and thus could not be evaluated based on this type of analysis. To summarize, the key aspect of this model is that it involves the simultaneous initial population of two ^{3}MC states (yielding ESA B resp. C) which decay independently to the GS with lifetimes of 13 ps and 20 ps, respectively, after some initial ^{3}MC cooling taking place on a 610 fs timescale.

Results from NatComm model

Fit Results:

Model Used: External function

The minimum error is:2.37594860e-06

The minimum R2-value is:9.98528703e-01

The rates converted to times with unit ps

k0 9.0

k1 1.5

k2 15.102349

br 0.6

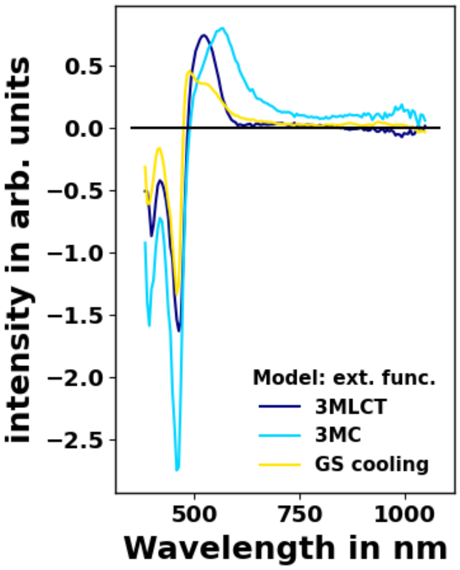

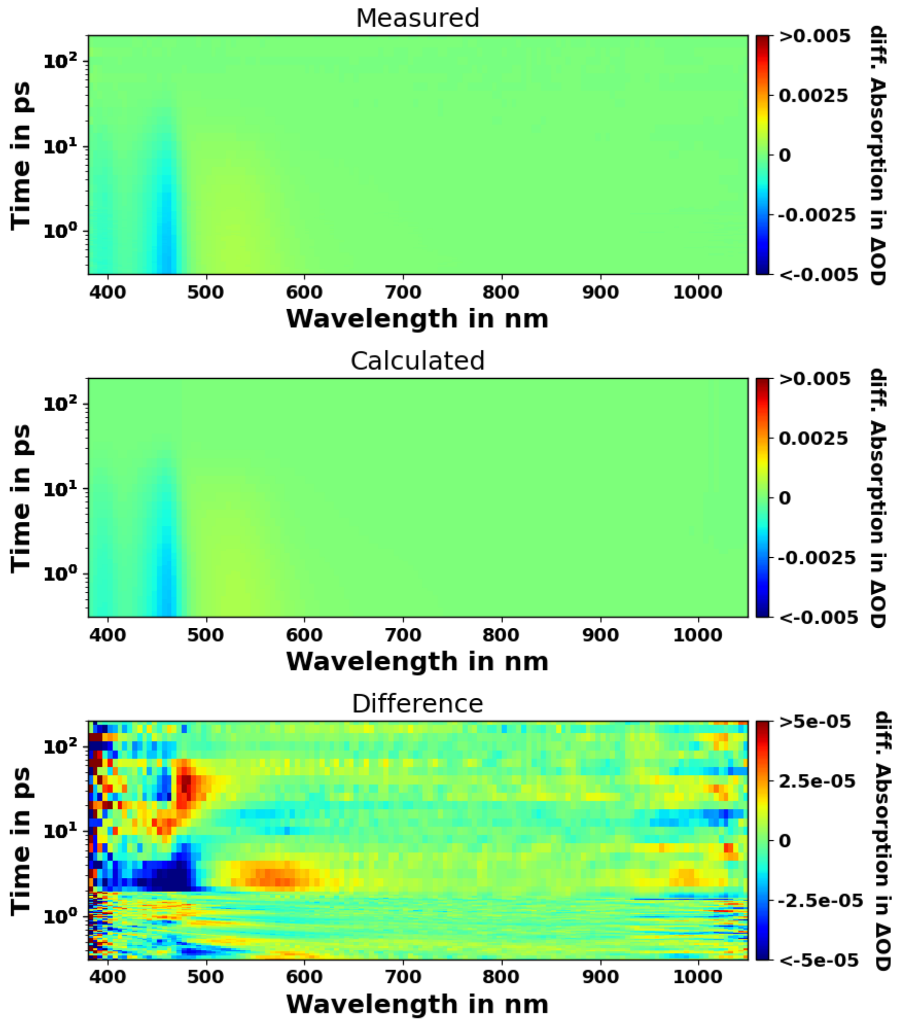

Figure SI.20. The model from the Nature Comm. paper, with fixed MC and MLCT lifetimes as well as the selected branching of 60% according to the publication. This fit results in clear non-random residuals and was therefore ruled out.

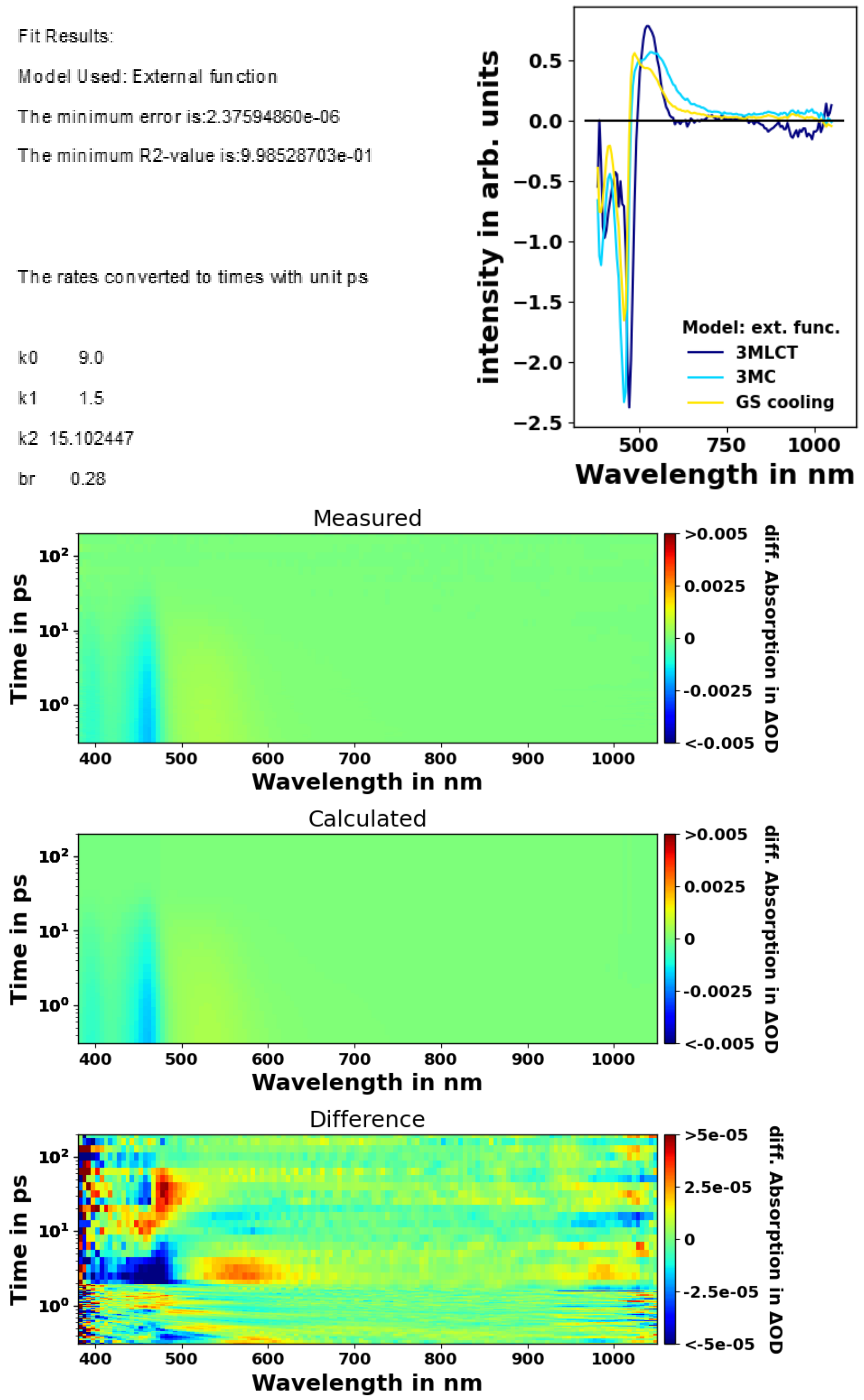

Figure SI.21. Again, the same model with fixed lifetimes from the Nature Comm. paper. Here the branching ratio was varied to try to match the extinction of the GSB in all SAS. This resulted in a branching ratio of 28% to 3MLCT. Even if the branching ratio is changed, there are still non-random residuals which ruled out this model.

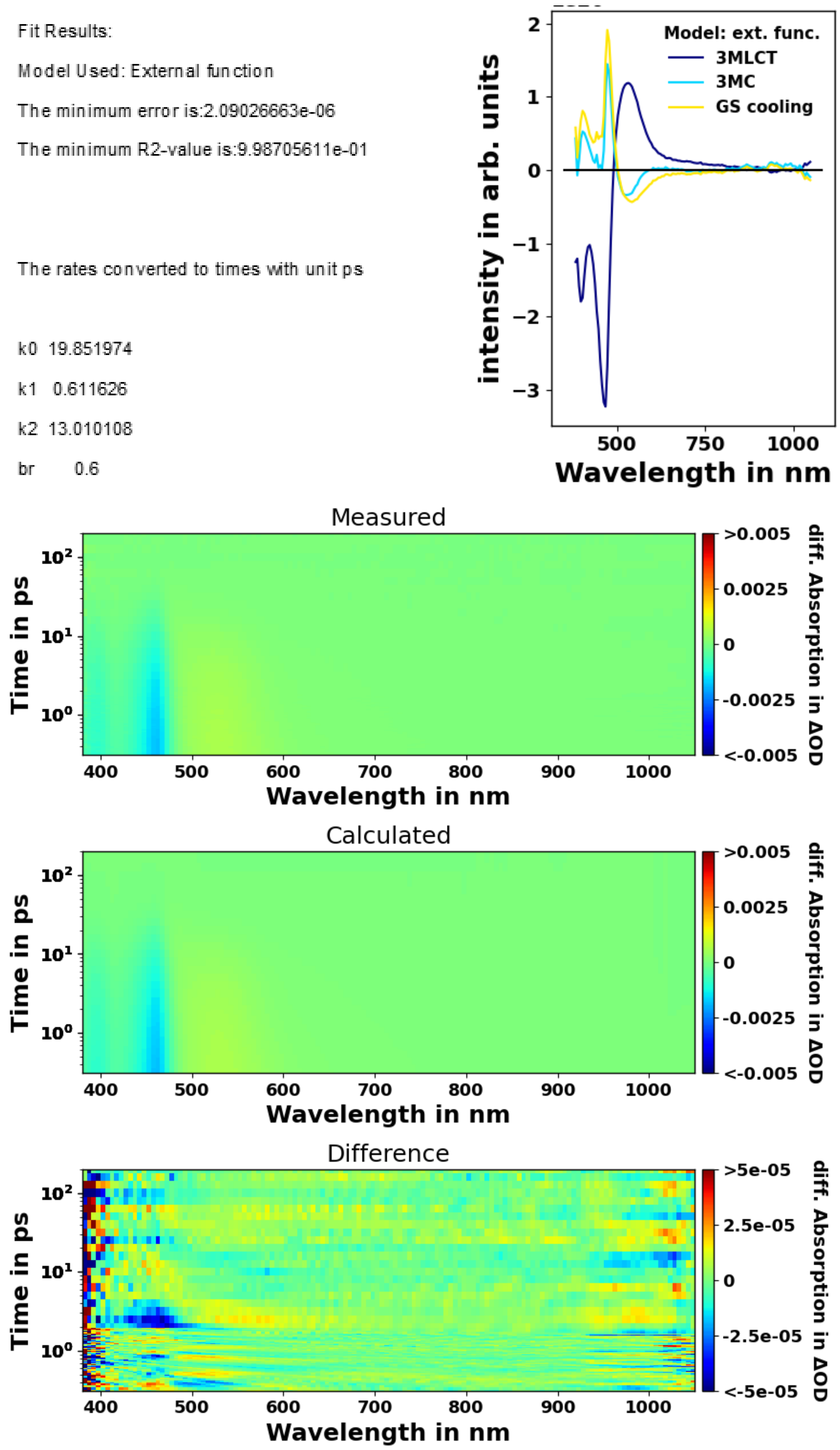

Figure SI.22. The model from Nat Comm but with freely fitted lifetimes. This results in lifetimes very different to those stated in the paper, and unphysical SAS with negative features outside the GSB spectral region. Therefore, this model was ruled out.

Results for the NatComm w. MC relax model

Fit Results:

Model Used: External function

The minimum error is:2.04841135e-06

The minimum R2-value is:9.98731529e-01

The rates converted to times with unit ps

k0 9.0

k1 0.847017

k2 1.5

k3 18.923493

br 0.6

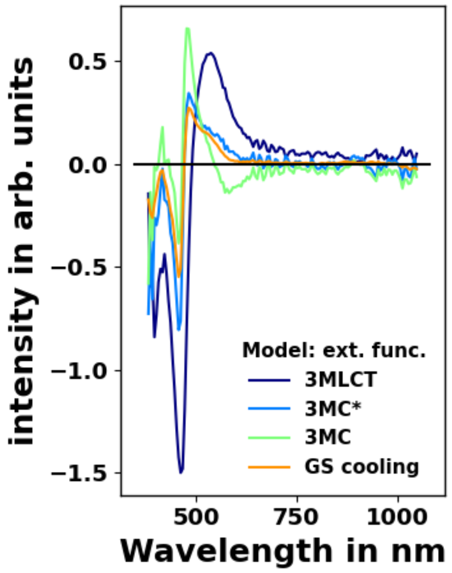

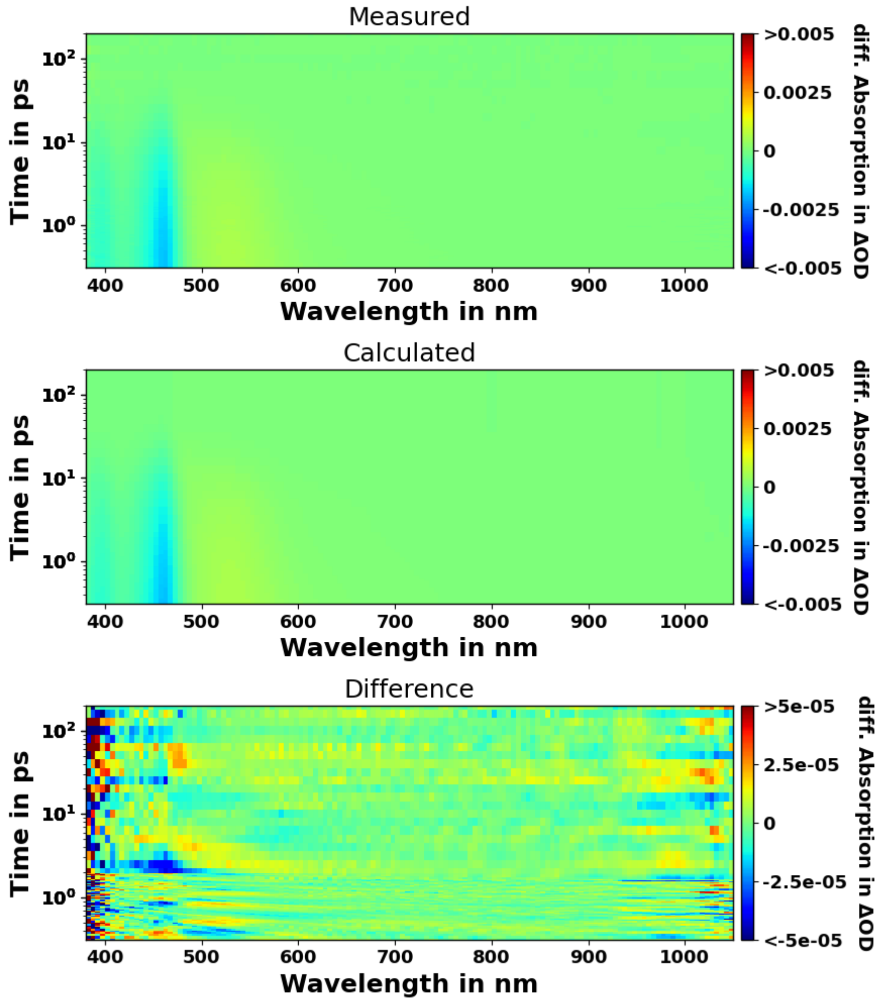

Figure SI.23. Initially the lifetime of the MLCT and MC states are locked according to the Nature Comm. paper. Also the specified branching of 60% was selected. The relaxation of the 3MC state and GS cooling are freely fitted. The model is ruled out based on the negative feature in the 3MC SAS in the ESA wavelength range. The branching ratio could not be varied to remove the negative SAS features in the ESA wavelength range.

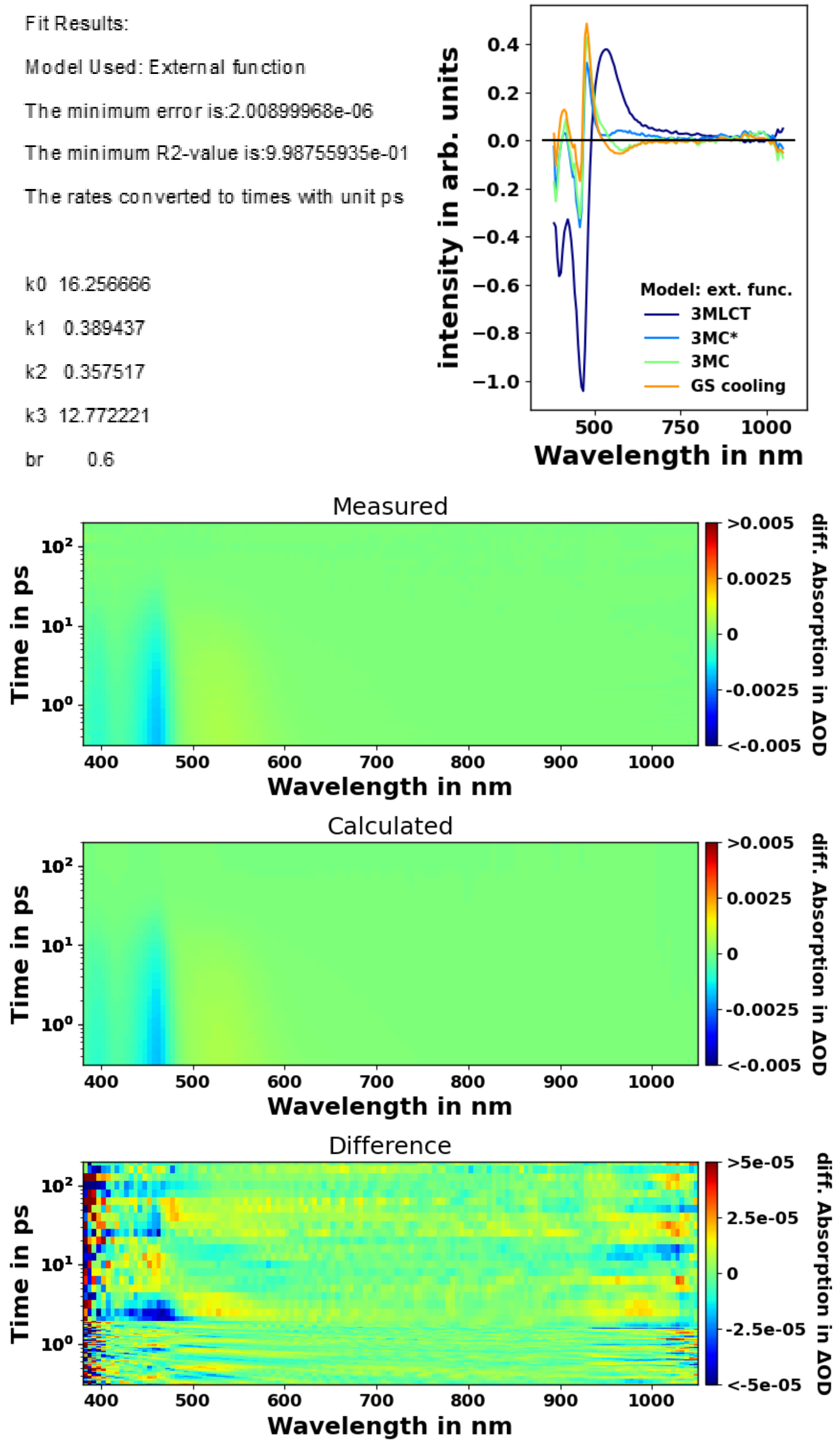

Figure SI.24. The same model but all lifetimes are freely fitted, but with the branching ratio set to 60% according to the Nature Comm. paper. Also here some of the SAS show unphysical negative components in the ESA spectral region, that is why this model was ruled out.

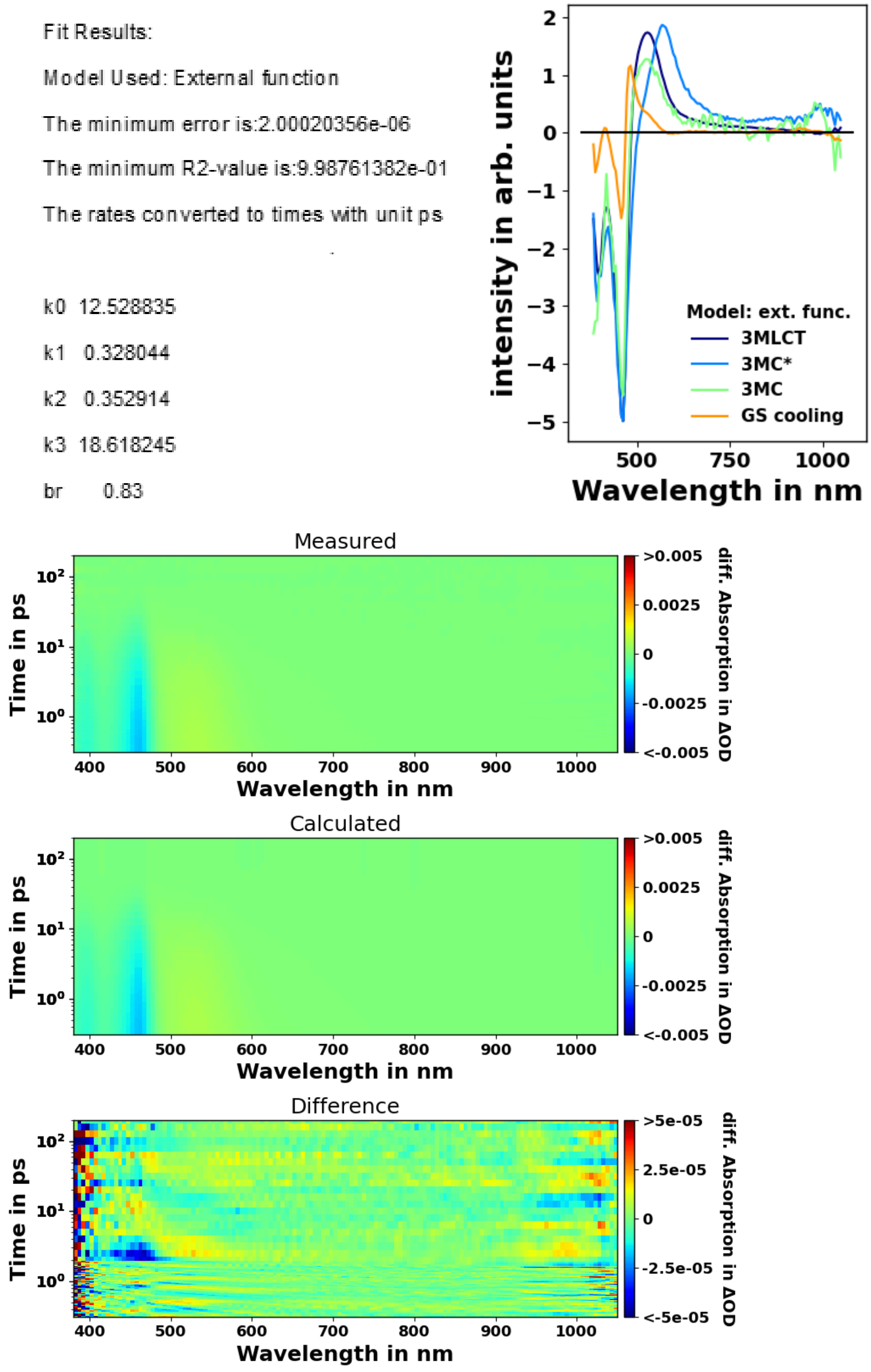

Figure SI.25. By changing the branching ratio, it is possible to find a parameter space where the lifetimes are freely fitted, yielding SAS with no negative features in the ESA spectral range. However, SAS of the hot 3MC state and the 3MC state should be similar. This is not the case, instead the 3MLCT and 3MC SAS are nearly identical. Since this does not match with the interpretation of the states, this model was ruled out as well.*

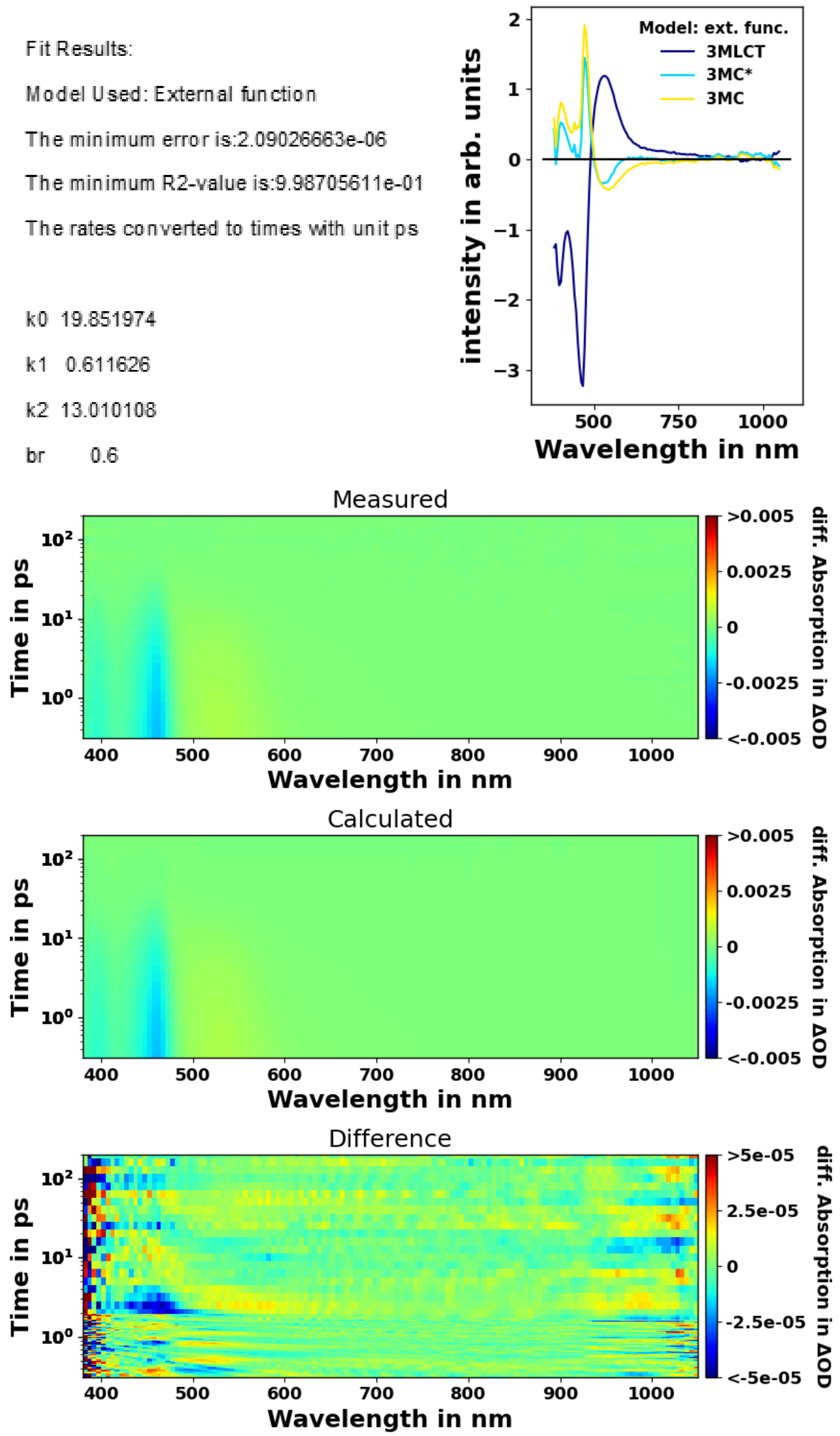

Figure SI.26. The NatComm model with MC relaxation but without the step of GS cooling. Here the rates were fitted freely, and the branching ratio was set to 60% according to the Nature Comm. paper. This model also shows SAS with unphysical negative features in the ESA spectral range. Varying the branching ratio did not resolve this problem, that is why this model was ruled out.

Results for the NatComm w. MLCT relax model

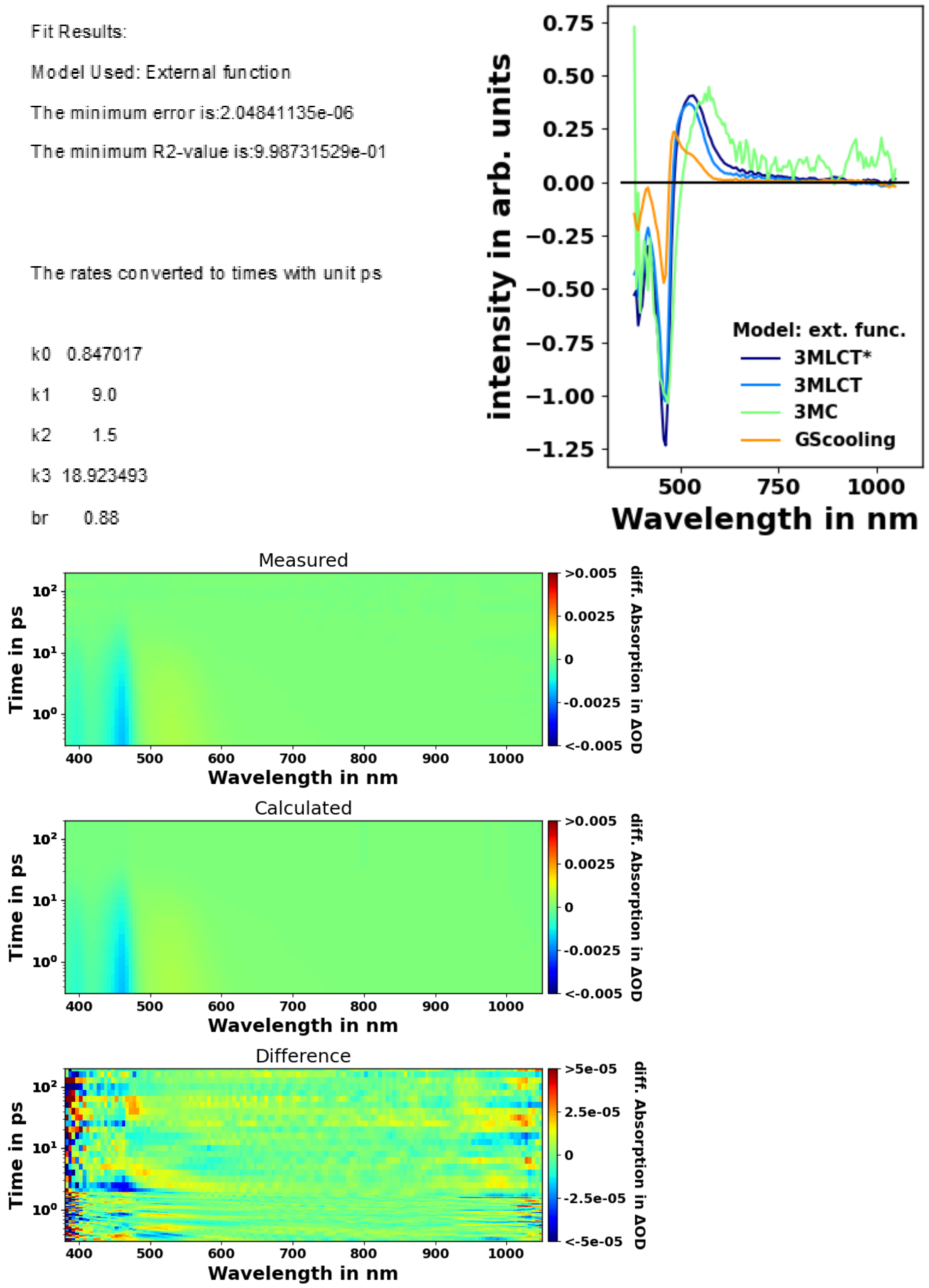

Figure SI.27. The model from Nature Comm. but with a relaxation step in the 3MLCT state. Here the lifetimes of the 3MLCT and 3MC states were locked according to the values in the paper, however cooling of the 3MLCT state and GS are freely fitted. The branching ratio is set to 88%, in order to yield equal extinction of GSB in all SAS. The SAS of 3MLCT and 3MLCT are similar which is reasonable. The 3MC SAS is also very similar in shape to the 3MLCT SAS but red-shifted. The ESA spectra associated with each state are of course not known, but it seems unlikely that they would be that similar. The GS cooling SAS still assumes a shape that we cannot explain by a broadened GSA together summed with the GSB signal.*

The errors are similar to the other best models, even though some components were locked. Here however, there is one more component in the fit compared to the other best results that can compensate for the locked components. All-in-all, the need for still locking components and adding one more compared to other models together with the SAS shapes rules this model out.

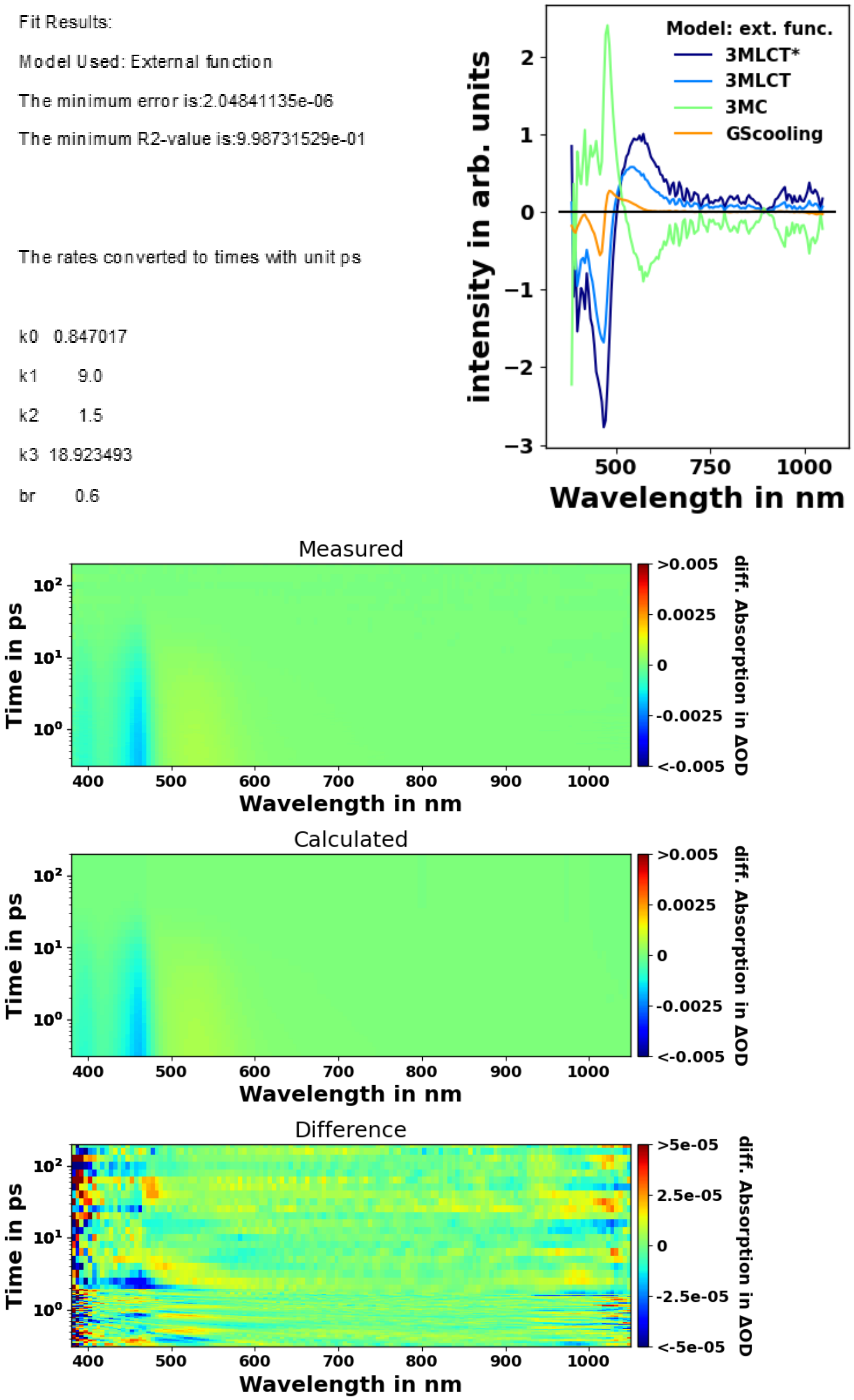

Figure SI.28. The exact same model, but with the 60% branching as specified in the Nature Comm. paper. With this branching the SAS yields unphysical negative features in the ESA spectral range. The model must therefore be ruled out. Setting the branching ratio to more than 75% was found as the limit for avoiding the unphysical negative SAS.

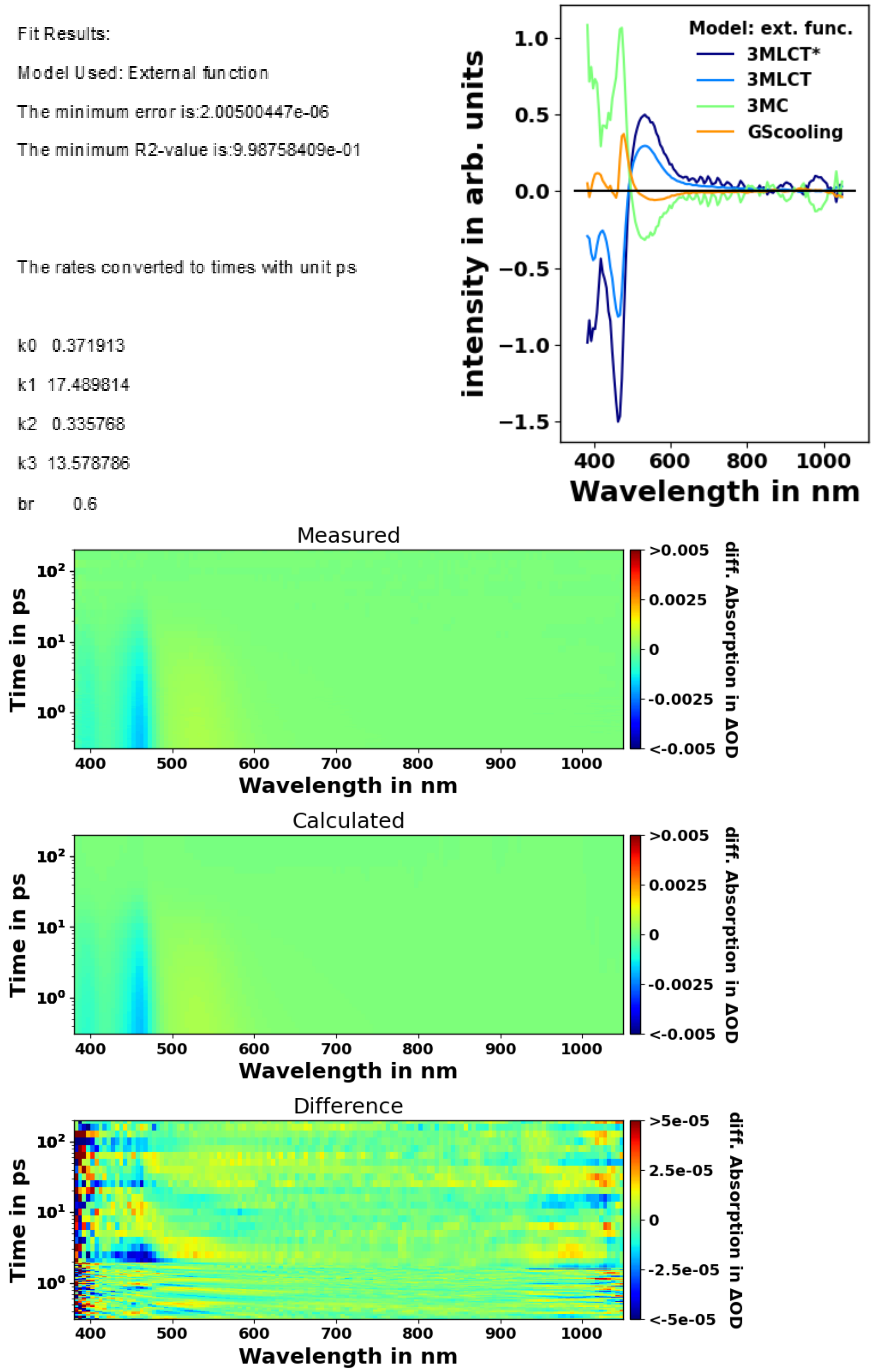

Figure SI.29. If all decay components are fitted freely in the model, they result in very different values to what was previously published. Also, the SAS yields unphysical negative features in the ESA spectral range. This model was therefore ruled out.

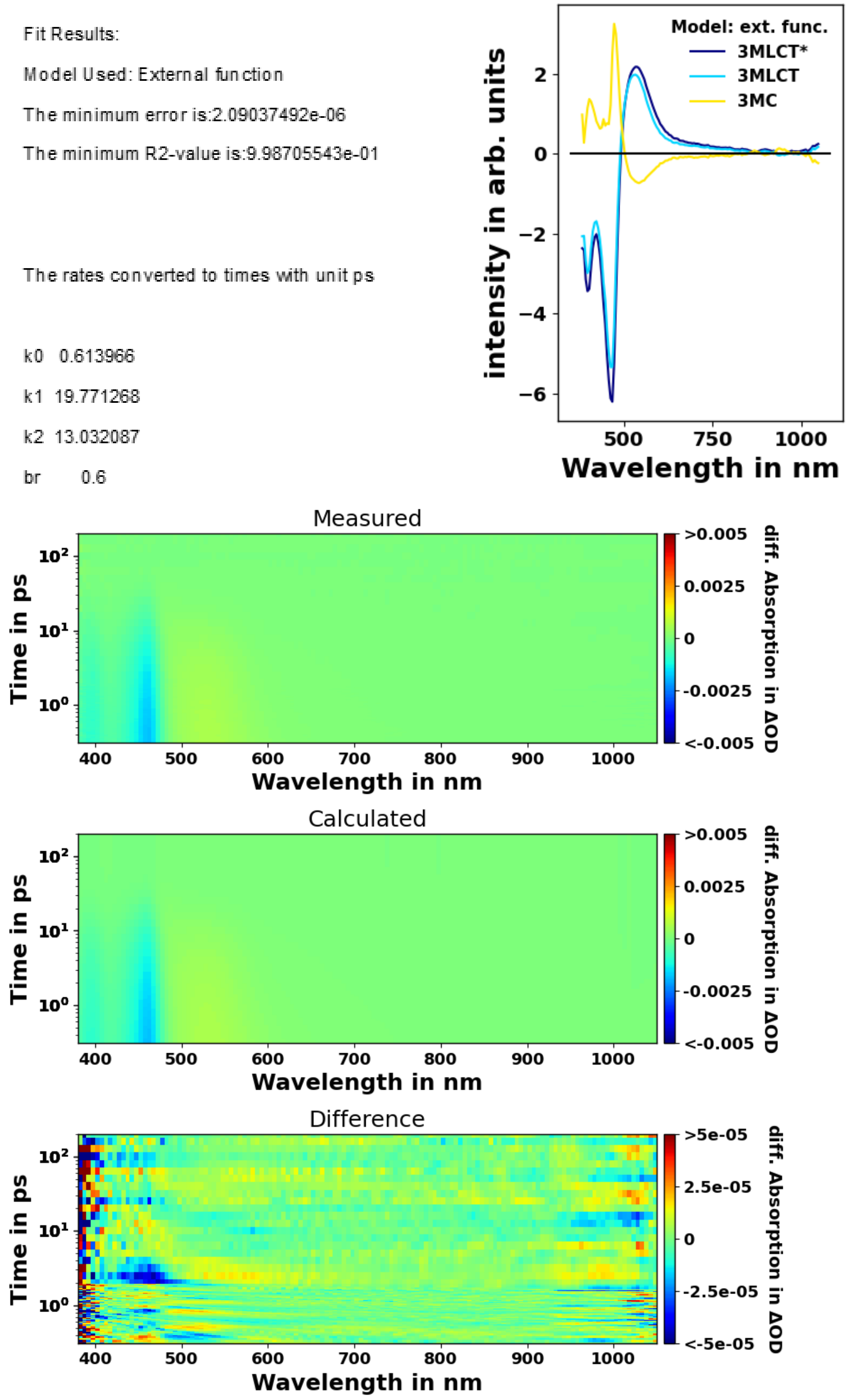

Figure SI.30. The same model, NatComm w. MLCT relax but without the GS cooling. Here all decay components are freely fitted with the specified branching of 60%. Again, the decay components assume very different lifetimes to those published, and the 3MC SAS shows unphysical negative features in the ESA spectral range. This model was therefore ruled out.

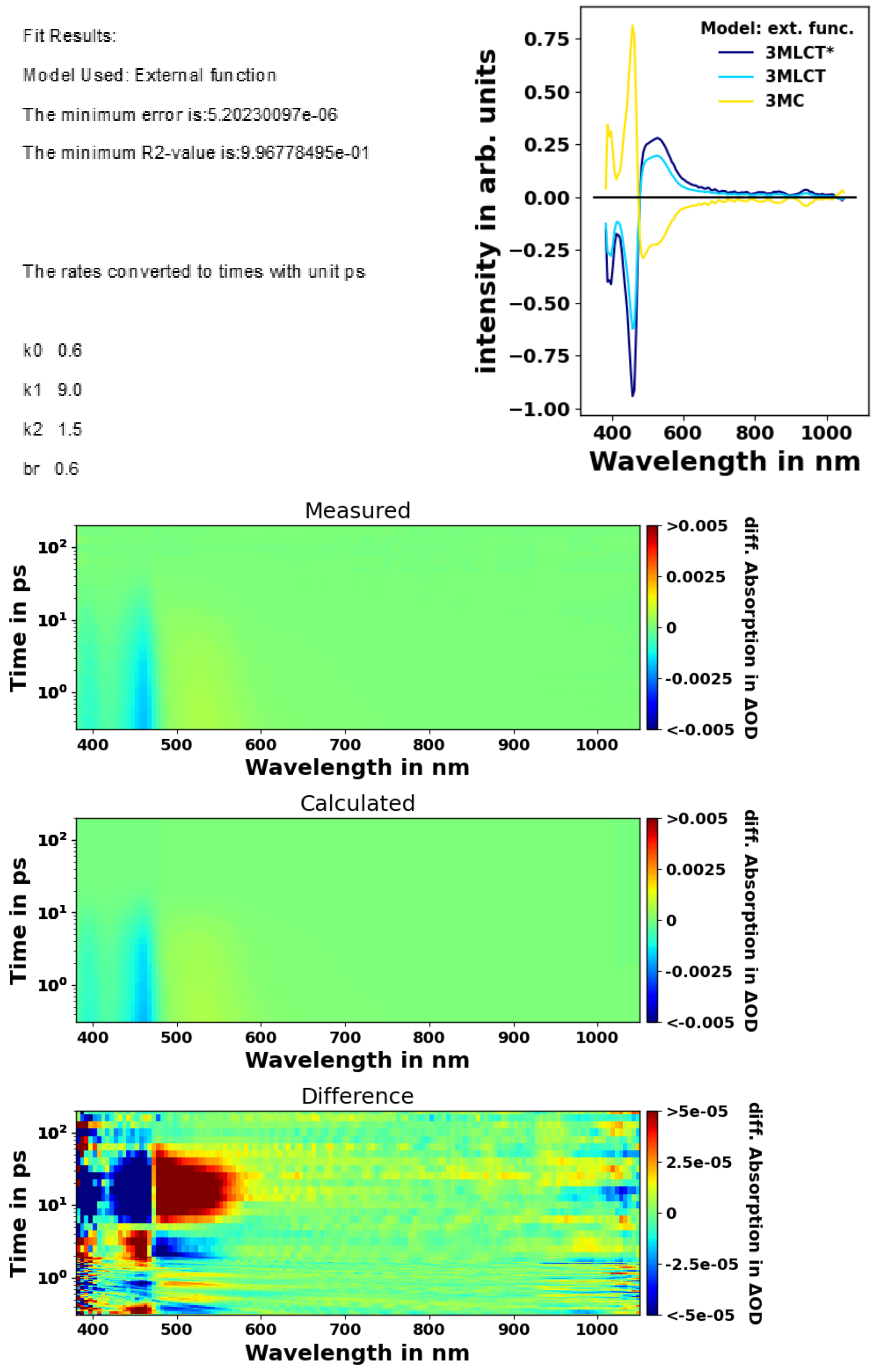

Figure SI.31. The same model, NatComm with MLCT relaxation but without the GS cooling. Here all decay components were locked to previously published values, and the branching ratio was set to the previously specified 60%. For this fit the residuals are large, and the 3MC SAS shows unphysical negative features in the ESA spectral range. This model was therefore ruled out.

Results for the Consecutive model

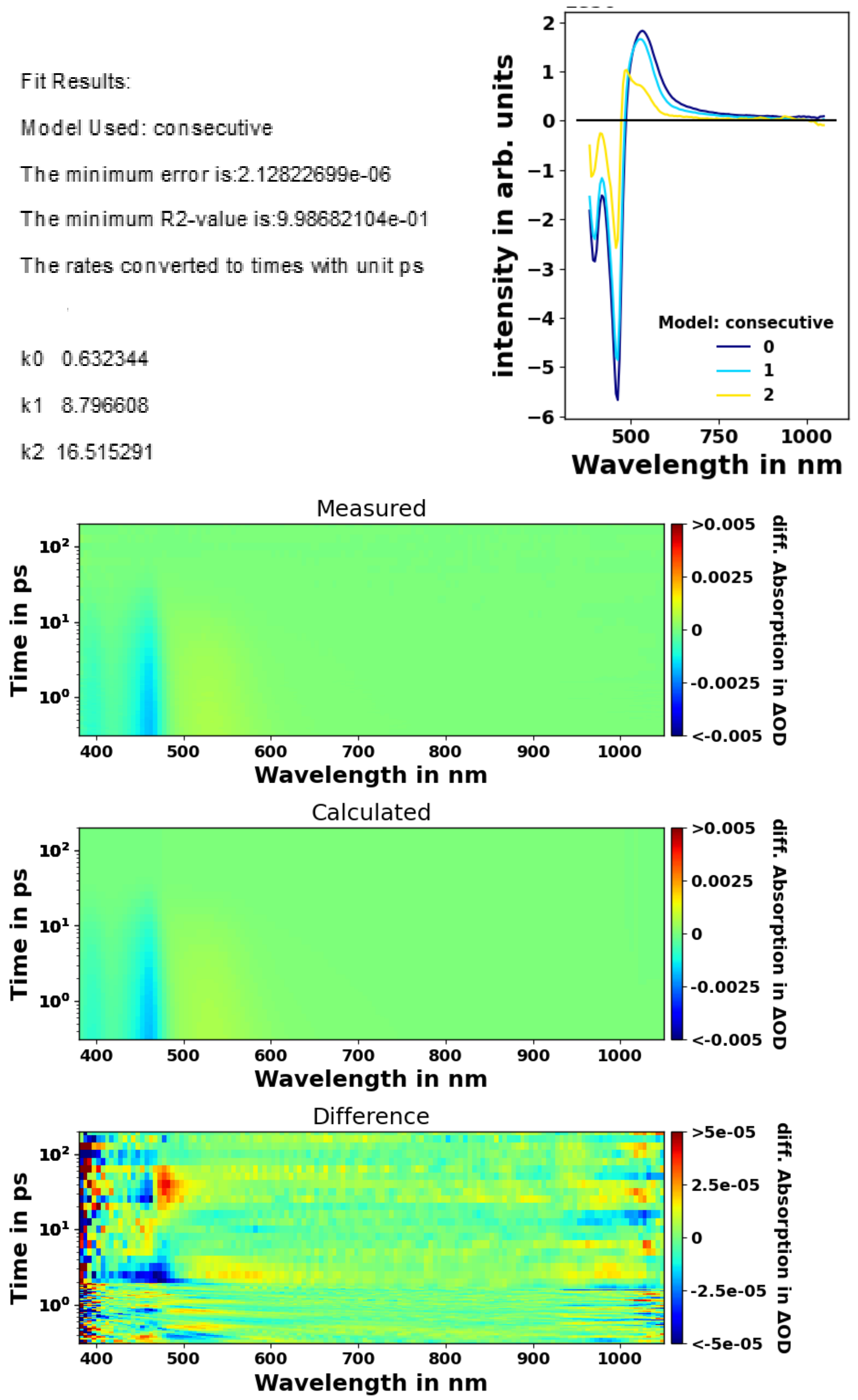

Figure SI.32. A purely consecutive model with 3 components, here in order from shortest to longest. This is the only order that works without yielding unphysical SAS with negative features in the ESA spectral range. This is one of the fits yielding lowest residuals and acceptable SAS. Nevertheless, there are fits with branching with less residuals, but they also have more fitted parameters.

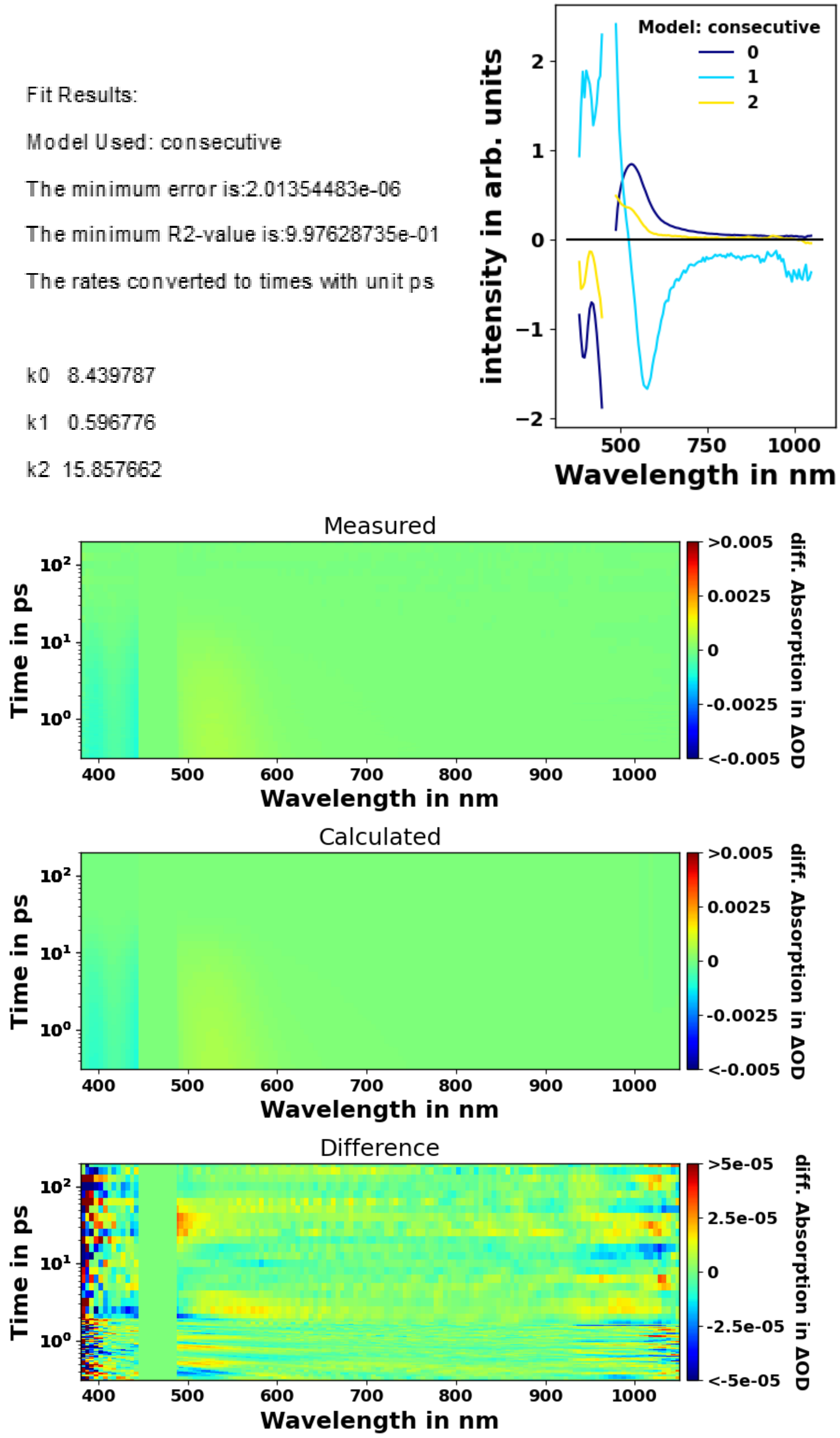

Figure SI.33. Again, a consecutive model with the similar 3 components, however in another order. This resulted in one unphysical SAS showing negative features in the ESA spectral range. Therefore, the model was ruled out.

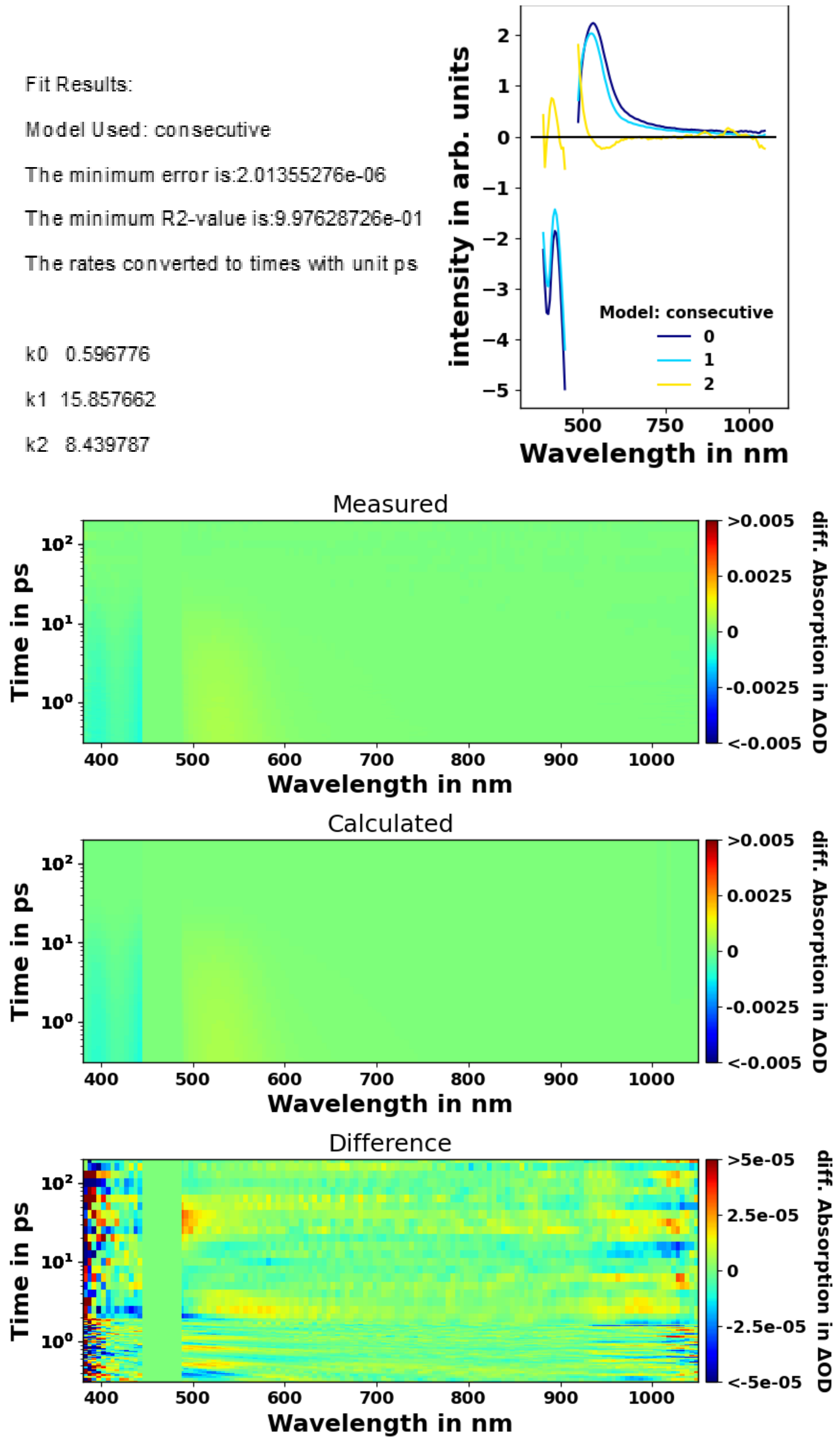

Figure SI.34. A consecutive model with the similar 3 components, however in another order. This resulted in one unphysical SAS showing negative features in the ESA spectral range. Therefore, the model was ruled out.

Results from the Parallel2 model

Fit Results:

Model Used: External function

The minimum error is:2.09037468e-06

The minimum R2-value is:9.98705544e-01

In Rates

The rates converted to times with unit ps

k0 13.032343

k1 0.613977

k2 19.76989

br 0.6

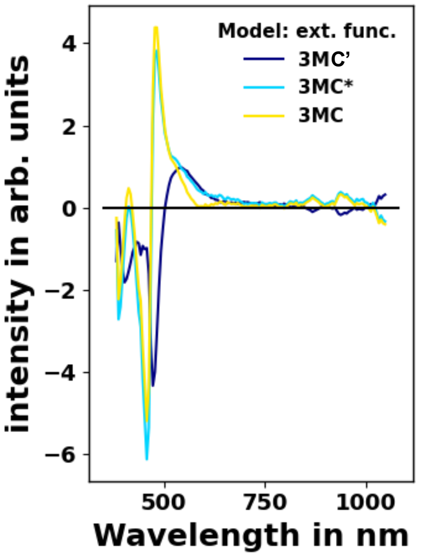

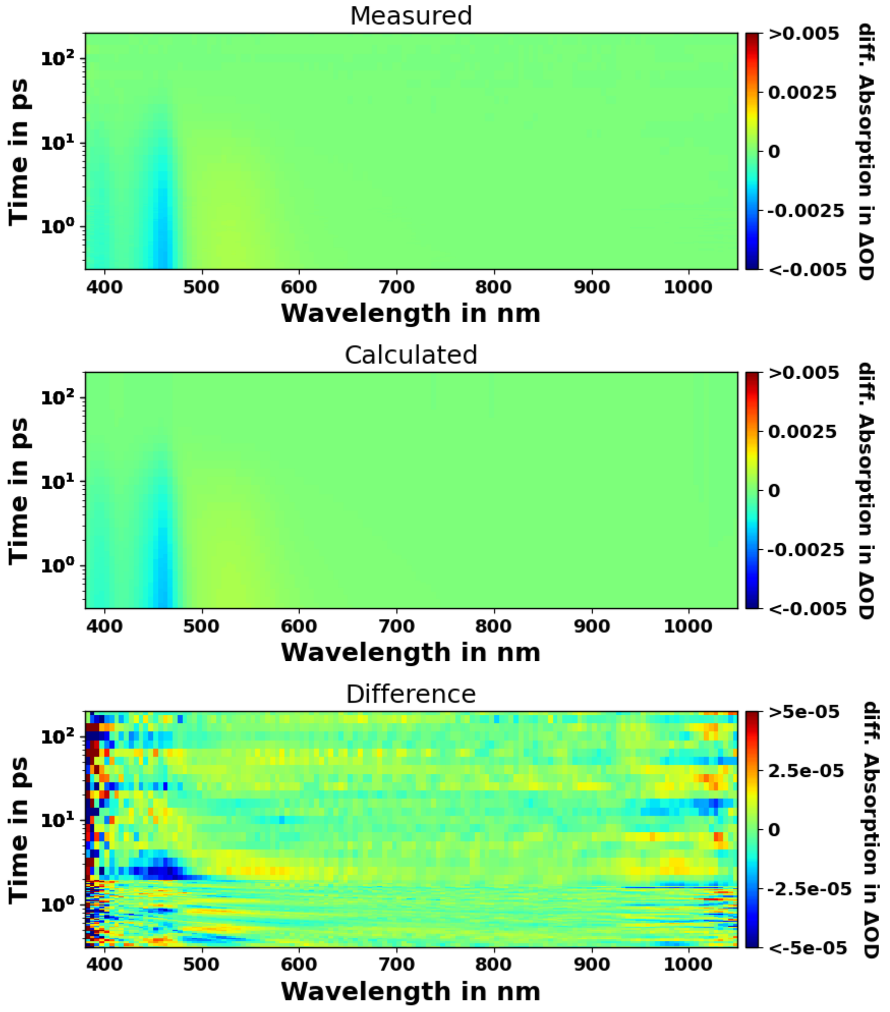

Figure SI.35. In this model, two states 3MC and 3MC' decay in parallel, and one of the states (3MC) has a relaxation step before depopulation. Here the branching ratio was set to the specified 60% based on the Nature Comm. paper. This model is one of the best when it comes to residuals, and also yields SAS that can be interpreted in line with the model.

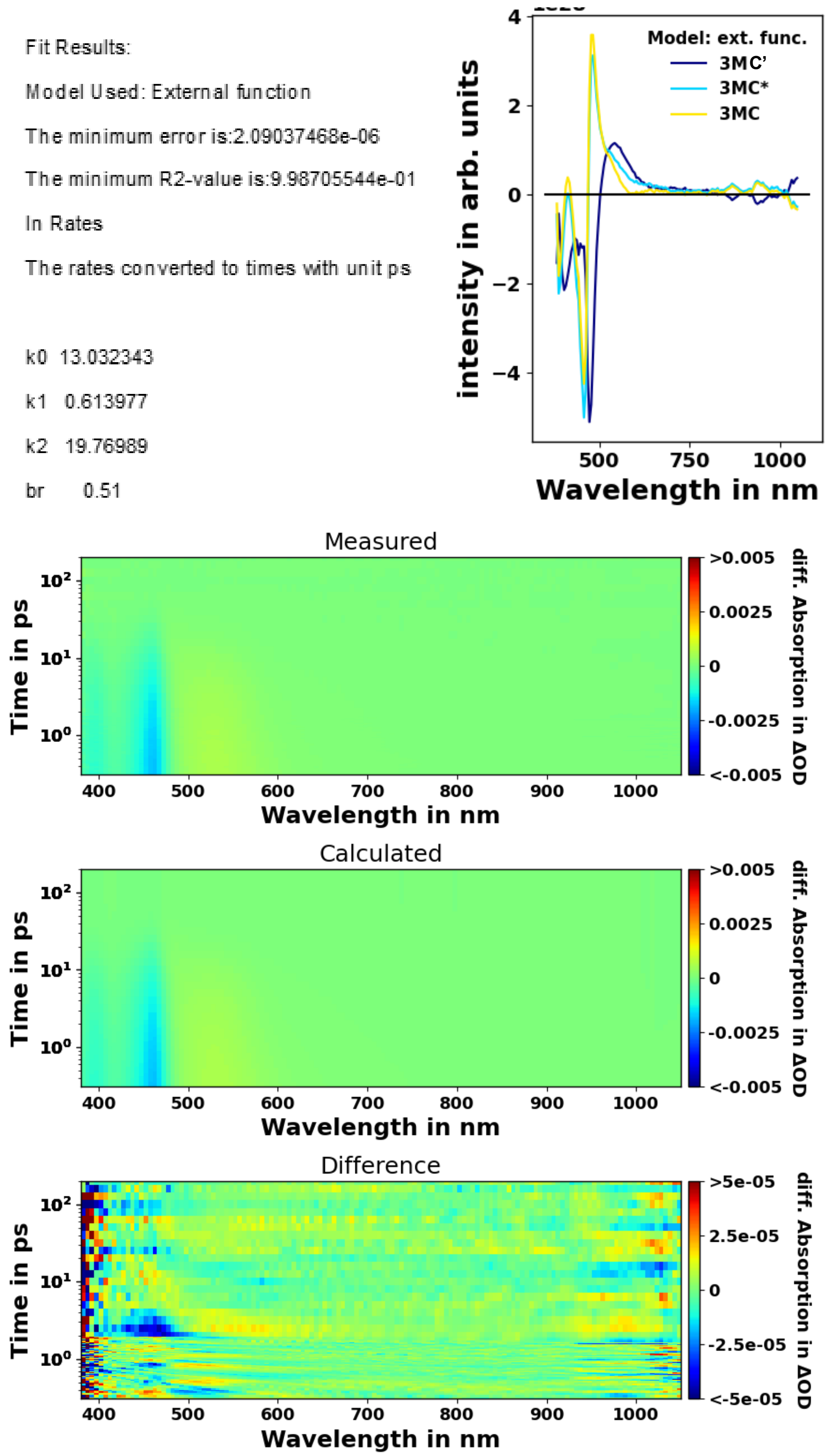

Figure SI.36. The same model, but with the branching ratio set to 51% based on yielding similar GSB extinction in all SAS. Since the SAS do not show the exact same GSB feature, it is not possible to scale the branching ratio by matching the GSB extinctions therefore we cannot make any conclusions about it from our data.

Fit Results:

Model Used: External function

The minimum error is:2.09037471e-06

The minimum R2-value is:9.98705544e-01

The rates converted to times with unit ps

k0 19.770496

k1 0.613949

k2 13.031487

br 0.42

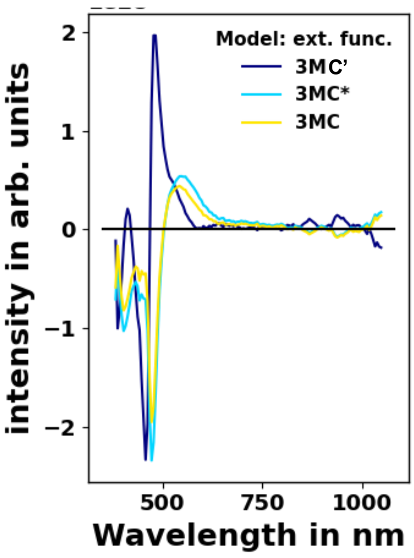

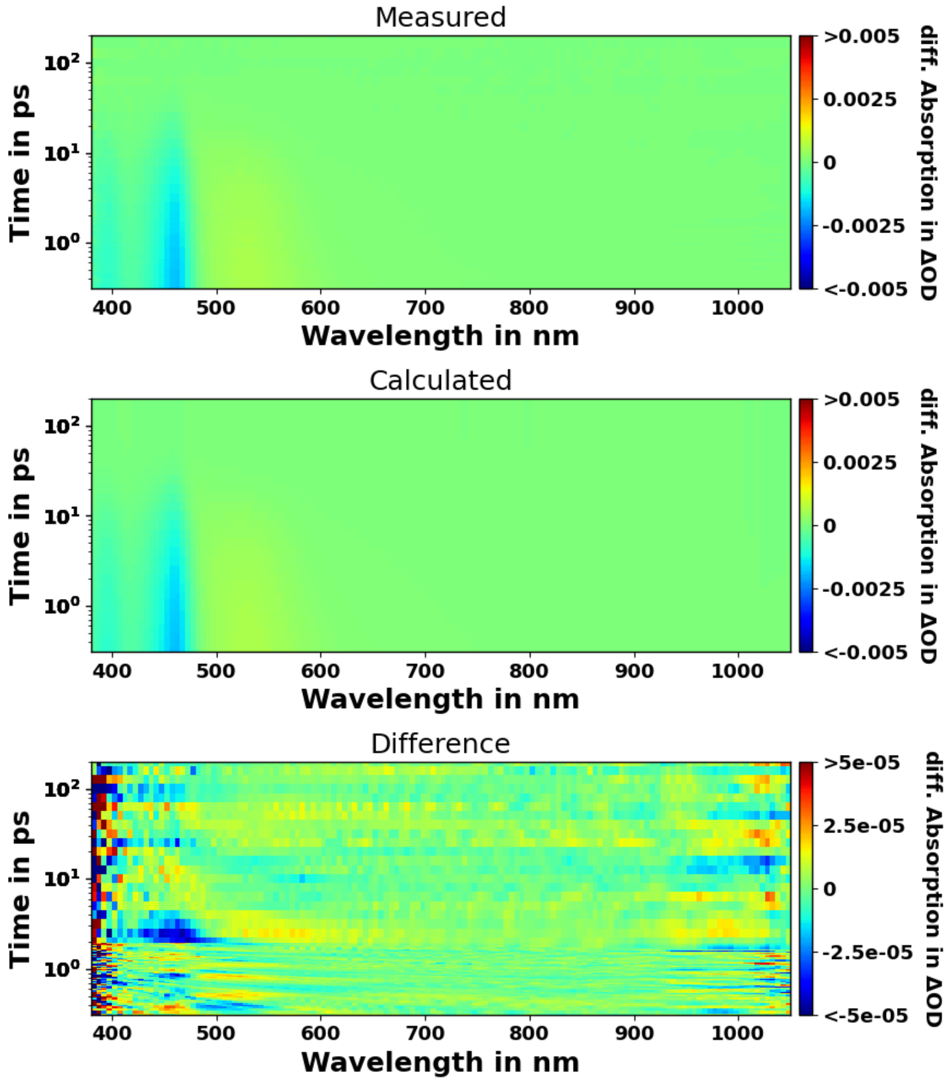

Figure SI.37. The same model, but with the other state having the relaxation step. This fit is equally valid in terms of residuals and interpretable SAS. Here the branching ratio was set to 42% based on yielding similar GSB extinction in all SAS. Since the SAS do not show the exact same GSB feature, it is not possible to scale the branching ratio by matching the GSB extinctions therefore we cannot make any conclusions about it from our data.

7. Anisotropy

The anisotropy signal was calculated by subtracting the perpendicular dataset from the magic angle dataset, and then dividing with the magic angle dataset. Special attention was paid to the single kinetics, and to double check that the background- and chirp corrections were good enough, see the data plotted in Figure SI.38. Since still the relative time zero between MA and L kinetics could differ on <100 fs precision, anisotropy kinetics were only evaluated after this time. Furthermore, when the TA signals approach low values at >30 ps delays the signal-to-noise of the anisotropy signal degrades. Therefore, the anisotropy kinetics cannot be evaluated at much later delay times than ~30 ps. To be in a reliable time-range with high enough signal-to-noise and minimum effect from potential errors in the chirp-correction, anisotropy spectra were plotted from 300 fs to 20 ps see Figure SI.39. In the anisotropy signal of complex **1**, we have seen that the ground state bleach anisotropy is not changing much within this time window. This means that the observed change of anisotropy in other spectral regions is not due to reorientation dynamics of the molecule and solvent, which also agrees with the longer reorientation time expected for rather bulky transition metal complexes (see for example Malone et al. and Wallin et al.).[5,6] Since all complexes share a similar molecular structure, we expect that also **2** and **3** will not show reorientation dynamics on the sub-20 ps timescale. Therefore, we consider it safe to look at perpendicular polarisation TA data for the population dynamics on sub-20 ps time scale in all complexes.

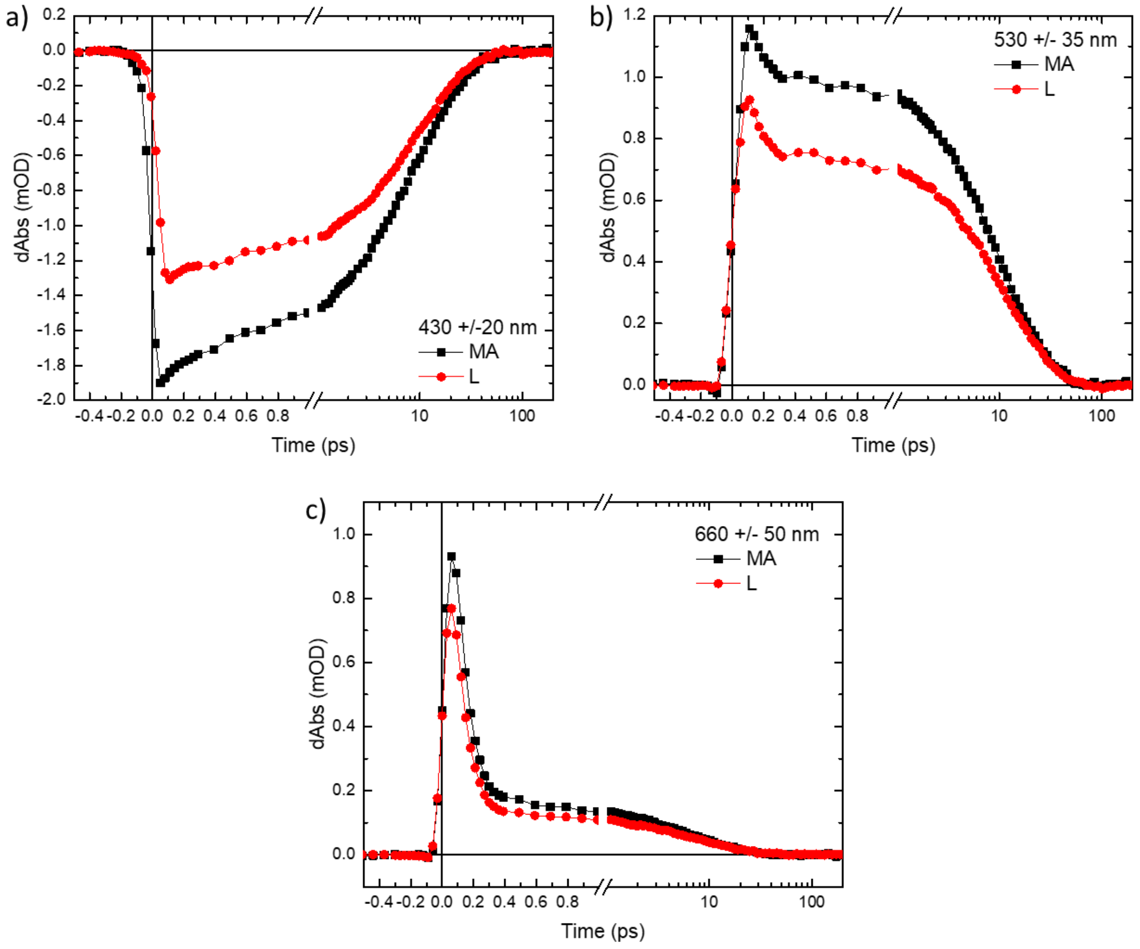

Figure SI.38. TA kinetics measured at magic angle (MA) or perpendicular (L) polarisation between the pump and the probe beam used for constructing the anisotropy kinetics. a) The 430 nm kinetic, b) the 530 nm kinetic and c) the 660 nm kinetic. Data was corrected for chirp and background.

In Figure SI.39, selected anisotropy spectra of **1** are shown together with the TA spectrum at 10 ps. Interestingly, in the part of GSB not contaminated by scattering pump set at magic angle the anisotropy is nearly the same at all delay times. This confirms that the change of anisotropy in other wavelength ranges should be related to state-to-state transitions rather than to reorientation dynamics.[5,6] It is important to note that a hot GSA (considered as a potential assignment of the ESA C feature) should exhibit anisotropy close to the rest of the GSA range as the dipole moment orientation of the "hot" and thermalized GS must be the same. In the case of GS cooling, all competing ESA should largely vanish at this stage of energy relaxation and the conversion of the ^{3}MLCT state into a "hot" GS should lead to an increase of anisotropy contrary to the observation. In the anisotropy kinetic at 530 nm, we fit instead a decay of 29 ps clearly different to the fitted biexponential 230 fs and 13 ps decay at 660 nm see Figure SI.40. All these considerations together argue that the 16 ps ESA C component cannot be dominated by a GS cooling process, but rather reflects an excited state decaying to GS.

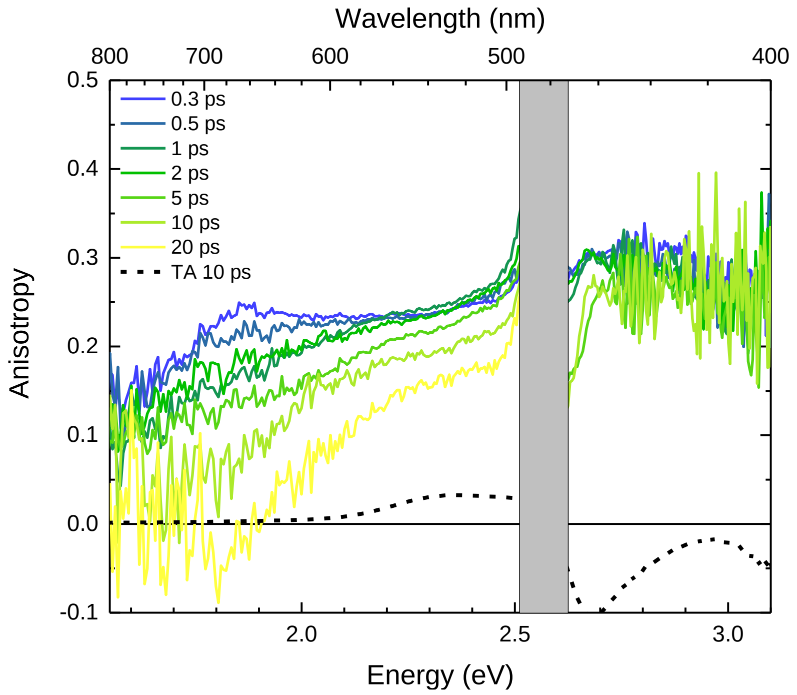

*Figure SI.39. Anisotropy spectra of **1** at selected delay times, cut to remove excitation scatter and corrected for background and chirp. In dashed line the TA spectrum at 10 ps is shown for comparison.*

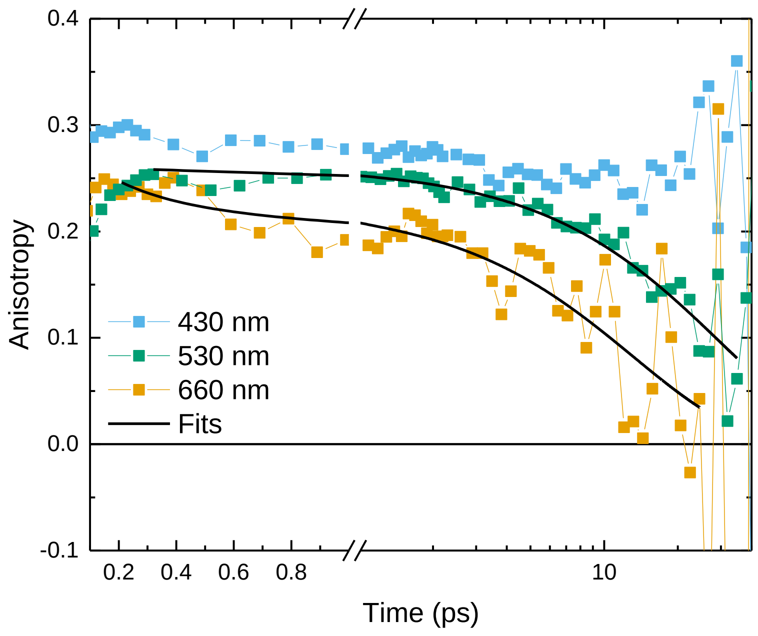

*Figure SI.40. Anisotropy kinetics of **1** at selected wavelengths, corrected for background and chirp. The 430 nm (2.9 eV) anisotropy is representative of the ground state bleach region, while the kinetics at 530 nm (2.3 eV) and 660 nm (1.9 eV) represent different excited state absorption anisotropies with associated fits discussed in the text shown as solid black lines.*

8. Temperature dependent TA fits

In this section we show the temperature dependent TA data fits by global analysis using the available software.[1] To avoid the early time artifact contribution, the data was cut away before 500 fs and the instrument response function (IRF) was set as 90 fs. The data was fitted in the ESA wavelength region by a single exponential function to get the major excited state lifetime required for the Arrhenius analysis, see Figures SI.41-46. The GSB region was not included in the fit due to the large pump scattering in this region.

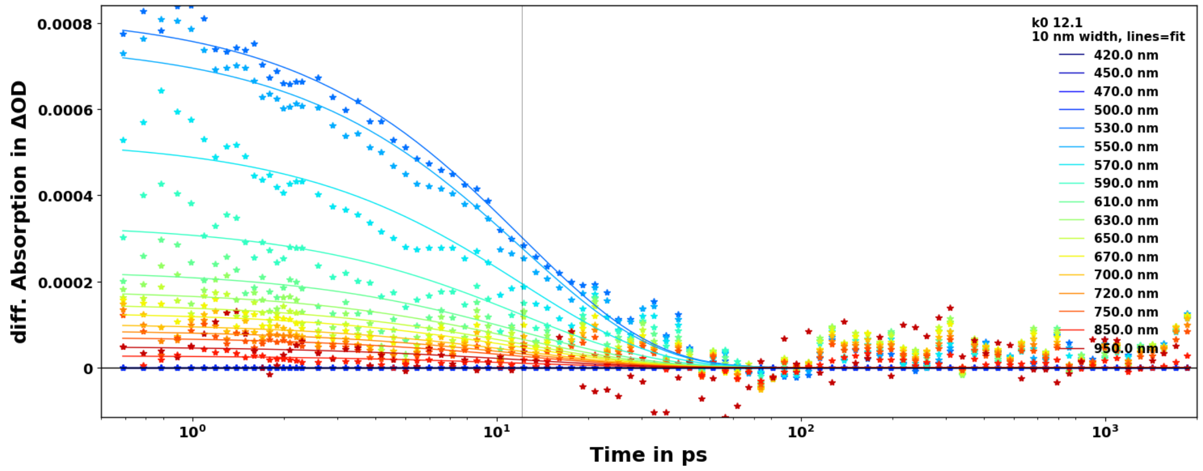

*Figure SI.41. Single exponential fit of the TA data of **1** at room temperature, shown are the fitted kinetics.*

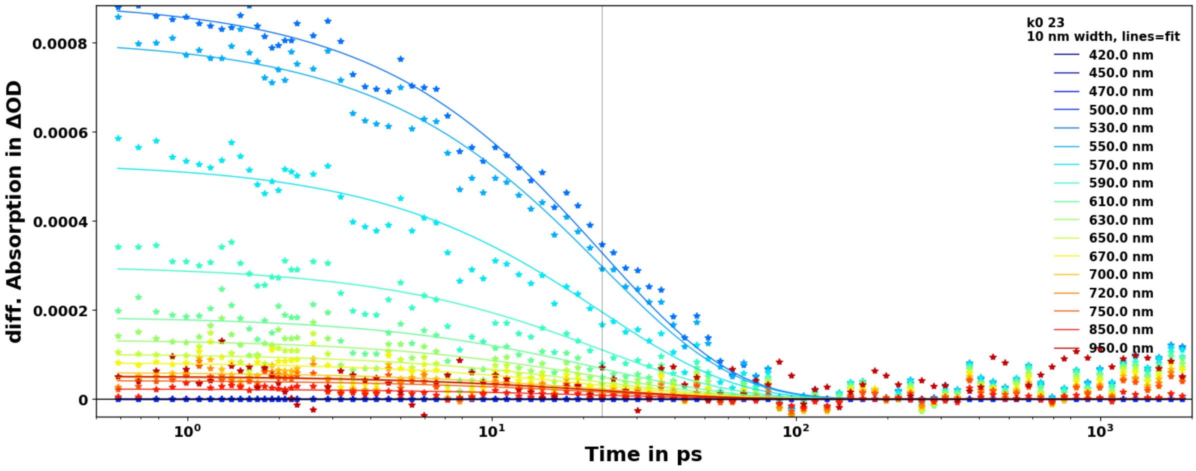

*Figure SI.42. Single exponential fit of the TA data of **1** at 230 K, shown are the fitted kinetics.*

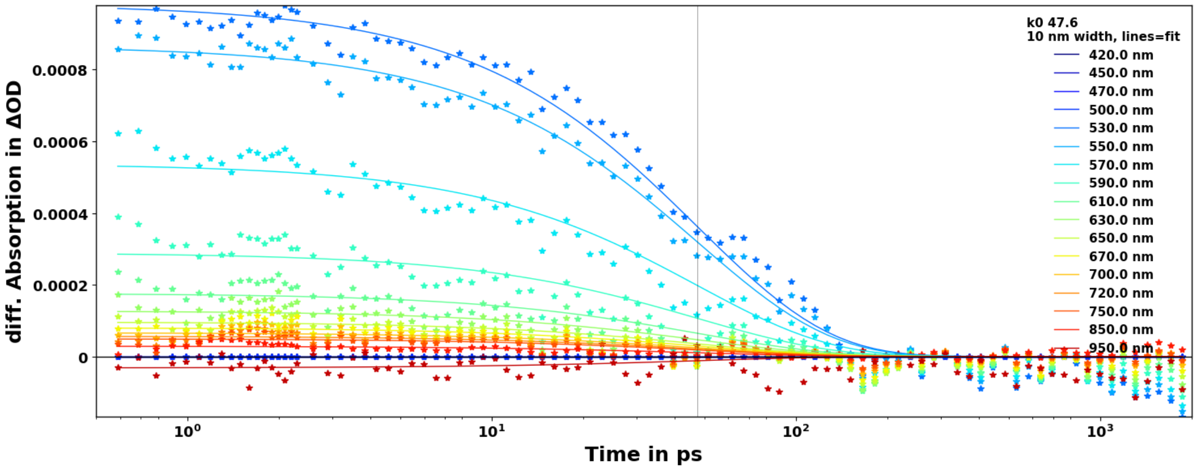

*Figure SI.43. Single exponential fit of the TA data of **1** at 180 K, shown are the fitted kinetics.*

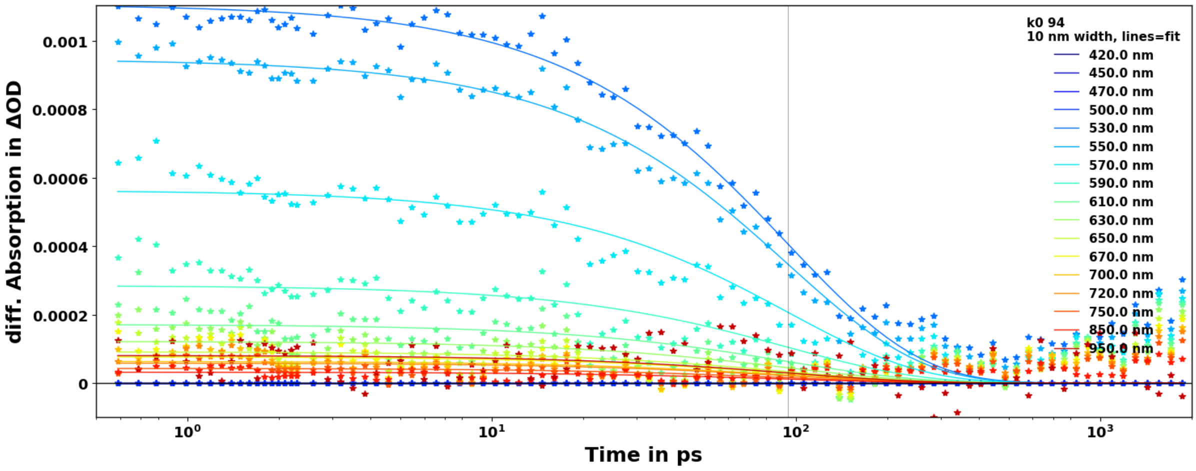

*Figure SI.44. Single exponential fit of the TA data of **1** at 160 K, shown are the fitted kinetics.*

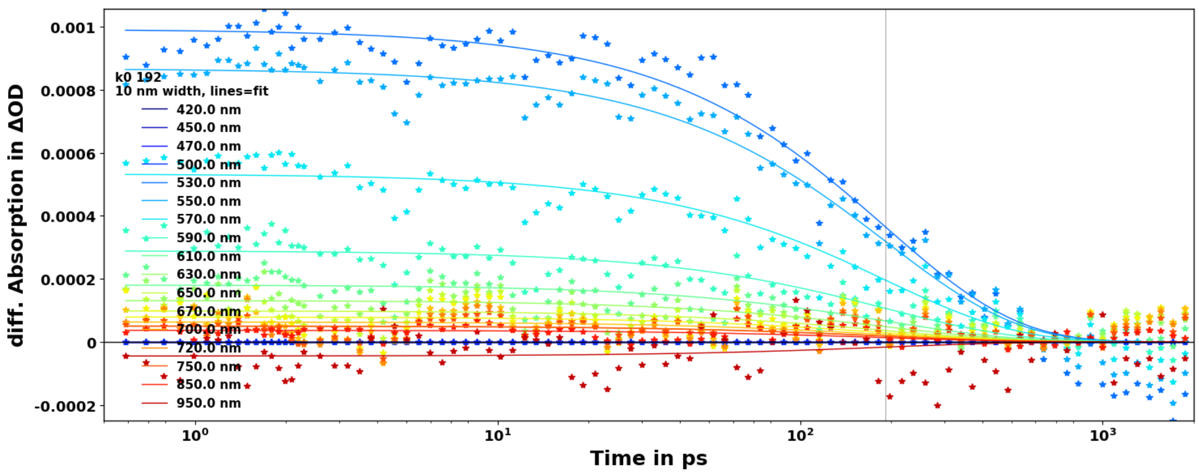

*Figure SI.45. Single exponential fit of the TA data of **1** at 130 K, shown are the fitted kinetics.*

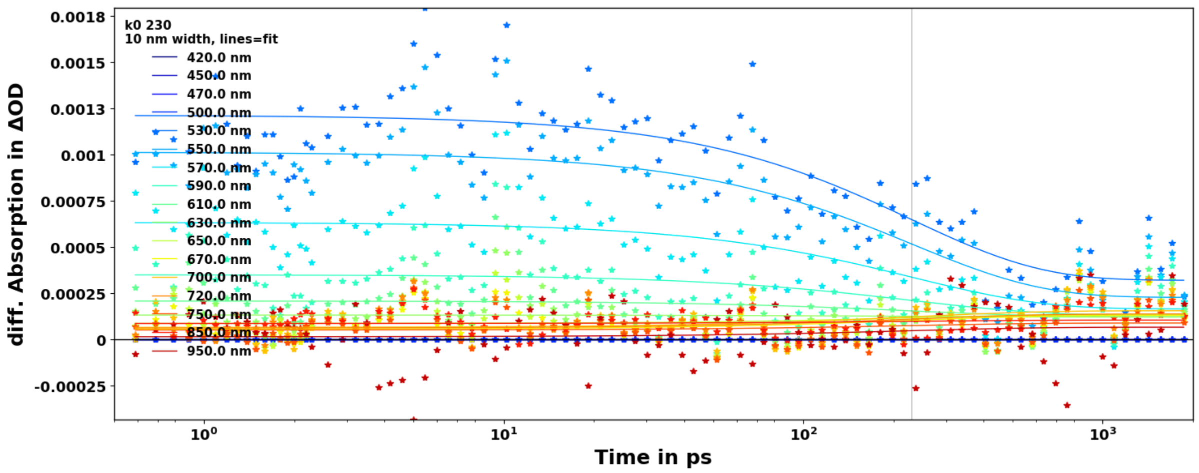

*Figure SI.46. Single exponential fit of the TA data of **1** at 120 K, shown are the fitted kinetics. Due to the bad signal-to-noise at this low temperature, a non-decaying component had to be added to account for the background offset).*

The Arrhenius fit to the temperature dependent excited state lifetime is shown in Figure SI.47. The fit was conducted for temperature points above the glass transition temperature (139 K). The fit function used was as follows:

$$\frac{1}{\tau_{obs}} = k_0 + A \cdot e^{-\frac{\Delta E}{RT}}$$

where τ_{obs} is the excited state lifetime, k_0 is the temperature-independent deactivation rate, A is the pre-exponential factor, ΔE is the energy barrier, R is the Boltzmann constant and T is the temperature.

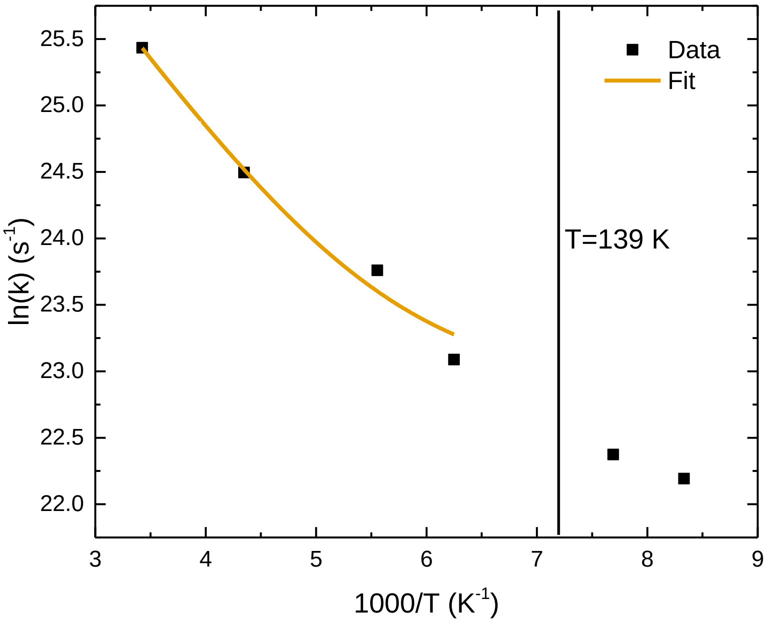

Figure SI.47. Temperature dependence of the major excited state lifetime in ***1****, with fitted Arrhenius expression. The glass transition temperature of butyronitrile (139 K) is marked by a vertical line.*

9. Stability

In Figure SI.48, we show the absorption spectrum for the samples used for TA measurements. This is a way for securing the stability of the sample, why the absorption after the measurement is also checked. As is evident from Figure SI.48, the absorption spectra are similar in all cases, except for the concentration. It is common that the concentration can increase during the measurement, due to solvent evaporation. This proves that the sample quality was good in all measurements, and even if case some part of the sample had degraded this could not have been excited by the excitation light (470 nm) as no new absorption was observed in the excitation wavelength region.

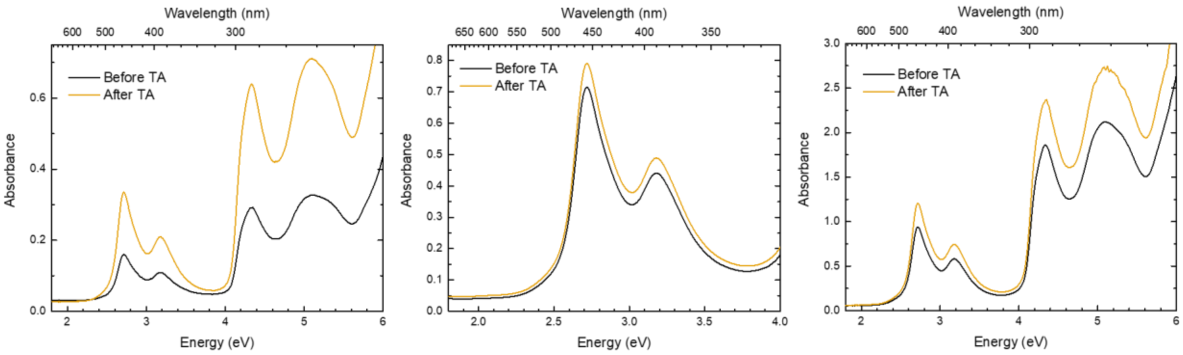

Figure SI.48. Absorption spectra of samples used for three different TA measurements before and after TA.

As a further line in assuring the quality of the measured samples, we also compare the quantified TA data measured by us and in Liu et al. and Kunnus et al.. In all three papers, the major decay component found was 9 ps. The decay associated spectrum associated with this component also looks similar in all three papers, with a peak ~530 nm (2.3 eV). Furthermore, all papers have

reported indications for a broad spectrum decaying on <100 fs timescale. The 16 ps decay component described here was also found in the paper by Kunnus et al., but not by Liu et al.. The reason for this is that the component is only substantially influencing the data in a narrow wavelength span around the red part of the ground state bleach region. This spectral region was first probed by Kunnus et al. since they used excitation wavelength 400 nm, and then by us since we used perpendicular polarization between pump and probe beams. The quantitatively same data found for complex **1** in all papers further assure the quality of the measured samples.

10. References

[1] Eriksson, A., Chabera, P. & Uhlig, J. KiMoPack: A python Package for Kinetic Modeling of the Chemical Mechanism. (2022) doi:10.1021/acs.jpca.2c00907.

[2] Duchanois, T. *et al.* An Iron-Based Photosensitizer with Extended Excited-State Lifetime: Photophysical and Photovoltaic Properties. *Eur J Inorg Chem* **2015**, 2469–2477 (2015).

[3] Liu, Y. *et al.* Towards longer-lived metal-to-ligand charge transfer states of iron(II) complexes: an N-heterocyclic carbene approach. *Chemical Communications* **49**, 6412 (2013).

[4] Kunnus, K. *et al.* Vibrational wavepacket dynamics in Fe carbene photosensitizer determined with femtosecond X-ray emission and scattering. *Nat Commun* 1–11 (2020) doi:10.1038/s41467-020-14468-w.

[5] Malone, R. A. & Kelley, D. F. Interligand electron transfer and transition state dynamics in Ru(II)trisbipyridine. *J Chem Phys* **95**, 8970–8976 (1991).

[6] Wallin, S., Davidsson, J., Modin, J. & Hammarström, L. Ultrafast Interligand Randomization of the MLCT State. *Journal of Physical Chemistry A* **109**, 4697–4704 (2005).
